# Reproductive tract and follicular fluid microbiota and probiotic interventions in IVF/ICSI outcomes: a systematic review

**DOI:** 10.1186/s12884-026-09523-1

**Published:** 2026-07-04

**Authors:** Guangyu Ma, Rujun Zeng, Rui Cai, Bixi He, Jinbiao Han, Rui Gao, Lang Qin

**Affiliations:** 1 Reproductive Medical Center, Department of Obstetrics and Gynecology, West China Second University Hospital, Sichuan University, Chengdu, China; 2 Key Laboratory of Birth Defects and Related Diseases of Women and Children, Ministry of Education, West China Second University Hospital, Sichuan University, Chengdu, China; 3 Department of Obstetrics and Gynecology, West China Second University Hospital, Sichuan University, Chengdu, China; 4 Meishan City Maternal and Child Health Hospital, Meishan, China

**Keywords:** Follicular fluid, In vitro fertilization, Intracytoplasmic sperm injection, Pregnancy outcomes, Probiotics, Reproductive tract microbiota

## Abstract

**Background:**

To investigate the associations of reproductive tract and follicular fluid microbiota composition, microbial diversity, and probiotic interventions with pregnancy outcomes after in vitro fertilization (IVF) or intracytoplasmic sperm injection (ICSI).

**Methods:**

PubMed, Embase, Web of Science, and the Cochrane Library were searched from January 2000 to August 2025, with manual screening of reference lists. Original human studies on women undergoing IVF/ICSI reporting reproductive tract or follicular fluid microbiota, pregnancy outcomes, or probiotic interventions were included, encompassing randomized controlled trials and observational studies. Two reviewers independently screened studies, extracted data, and assessed quality using RoB 2.0, ROBINS-I, and JBI tools. Due to heterogeneity in study designs, sampling sites, and microbiota methods, a qualitative synthesis was performed.

**Results:**

Among 2546 identified records, 40 studies met the inclusion criteria. Most studies consistently reported that a *Lactobacillus*-dominant reproductive tract microbiota, particularly *Lactobacillus crispatus*, was associated with higher implantation, clinical pregnancy, and live birth rates, whereas non-*Lactobacillus* or pathogen-enriched microbiota profiles were linked to poorer reproductive outcomes. Microbial diversity alone showed inconsistent associations with IVF/ICSI outcomes. Evidence regarding the influence of follicular fluid microbiota on pregnancy outcomes remains limited. Longer-term or individualized probiotic regimens appeared to increase *Lactobacillus* abundance and were associated with improved reproductive outcomes, whereas short-term interventions demonstrated minimal benefit.

**Conclusion:**

Species-level and site-specific reproductive tract microbiota profiles appear to better predict assisted reproductive technology outcomes than diversity measures alone. Personalized or longer-duration probiotic strategies may hold therapeutic potential; however, current evidence remains limited and heterogeneous. Well-designed longitudinal and site-specific trials are required to clarify the effectiveness of microbiota-targeted interventions in IVF/ICSI.

**Supplementary Information:**

The online version contains supplementary material available at https://doi.org/10.1186/s12884-026-09523-1.

## Introduction

Infertility affects 10–15% of couples of reproductive age worldwide [1]. Assisted reproductive technologies (ART), including in vitro fertilization (IVF) and intracytoplasmic sperm injection (ICSI), have significantly improved outcomes for patients with conditions such as tubal obstruction, male infertility, or recurrent implantation failure (RIF) [2–4]. Despite the availability of high-quality embryos, implantation failure and early pregnancy loss remain common, highlighting the importance of maternal factors, particularly the uterine environment and endometrial receptivity [5–7]. Data from over 900,000 ART cycles across 39 European countries show a clinical pregnancy rate of 34.6% per embryo transfer and a cumulative live birth rate of 30.8% per oocyte retrieval [8]. These rates, while promising, remain suboptimal, emphasizing the need to better understand the maternal biological factors influencing reproductive outcomes.

Recent studies have highlighted the role of the maternal reproductive tract microbiota as a key determinant of embryo implantation and pregnancy maintenance [9–11]. A healthy microbial community in the reproductive tract is typically dominated by *Lactobacillus* species, which maintain a low vaginal pH and inhibit pathogen growth through the production of lactic acid, hydrogen peroxide, and bacteriocins [12, 13]. These *Lactobacillus* spp. also interact with vaginal epithelial cells to modulate local immune responses, thereby maintaining immune homeostasis [14–16]. In contrast, depletion of *Lactobacillus* has been linked to increased proinflammatory cytokine levels, impaired endometrial receptivity, and reduced implantation potential [3, 17–19]. High-throughput sequencing studies suggest that a *Lactobacillus*-dominated microbiota is associated with higher clinical pregnancy, implantation, and live birth rates, although results remain inconsistent [20–25]. Several randomized controlled trials (RCTs) have explored probiotic supplementation to improve reproductive tract microbiota and enhance ART success, but findings are inconclusive [26–29].

In addition to the reproductive tract, recent research has identified microbial communities within the follicular microenvironment. Follicular fluid, which provides a critical environment for oocyte maturation, contains hormones, nutrients, cytokines, and signaling molecules essential for gamete development [30]. Once thought to be sterile, follicular fluid has now been shown to harbor distinct microbial signatures, even in the absence of clinical infection, as revealed by high-throughput sequencing [31, 32]. Genera such as *Actinomyces*, *Bifidobacterium*, *Propionibacterium*, and *Streptococcus* have been detected, some of which are associated with adverse pregnancy outcomes [33, 34]. These microorganisms, along with their inflammatory mediators, may disrupt oocyte maturation and embryonic development, potentially compromising IVF/ICSI success rates [33, 35].

Collectively, these findings underscore the pivotal role of both reproductive tract and follicular fluid microbiota in reproductive success. This systematic review aims to synthesize current evidence on microbial composition and diversity and their associations with IVF/ICSI outcomes, as well as evaluate the potential of probiotic interventions. A better understanding of microbiome-mediated mechanisms may inform strategies to optimize ART outcomes and guide personalized clinical interventions.

## Methods

### Protocol and registration

This systematic review was conducted in strict accordance with the Preferred Reporting Items for Systematic Reviews and Meta-Analyses (PRISMA) 2020 statement [36]. The study protocol was prospectively registered in the International Prospective Register of Systematic Reviews (PROSPERO) on October 26, 2025 (registration number: CRD420251176969) [37]. As the review was based solely on published data, ethical approval was not required.

### Search strategy

A systematic literature search was performed in PubMed, Embase, Web of Science, and the Cochrane Library to identify studies examining the female reproductive tract and follicular fluid microbiota in relation to IVF/ICSI pregnancy outcomes, including microbial diversity and probiotic interventions. The search covered publications from January 2000 to August 2025, was limited to English-language articles, and included peer-reviewed original research without restrictions on publication status. Reviews, commentaries, case reports, expert opinions, book chapters, critical appraisals, and conference abstracts were excluded.

The search strategy combined controlled vocabulary terms and free-text keywords, covering four conceptual domains: 1) Reproductive tract and follicular fluid (e.g., “reproductive tract,” “vaginal,” “cervix,” “uterus,” “endometrial,” “follicular fluid,” “ovarian follicle,” “oocyte”); 2) Microbiota and probiotics (e.g., “microbiota,” “microbiome,” “bacteria,” “flora,” “probiotic,” “*Lactobacillus*,” “*Bifidobacterium*”); 3) ART (e.g., “in vitro fertilization,” “IVF,” “intracytoplasmic sperm injection,” “ICSI,” “assisted reproductive technology,” “ART”); 4) Pregnancy outcomes (e.g., “pregnancy outcome,” “outcome,” “pregnancy,” “pregnancy rate,” “implantation failure,” “embryo implantation,” “live birth,” “miscarriage,” “early pregnancy loss”). Boolean operators were applied to ensure comprehensive retrieval: OR was used within each conceptual domain and AND between domains. Database-specific adaptations were applied, using MeSH in PubMed, Emtree in Embase, and free-text keywords in Web of Science and the Cochrane Library. Reference lists of included studies were manually screened to identify additional relevant publications. Detailed search strategies are provided in Supplementary Table 1.

### Inclusion and exclusion criteria

The inclusion and exclusion criteria were defined according to the PICO (Population, Intervention/Exposure, Comparison, Outcome) framework.

Inclusion criteria: 1) Studies involving women of reproductive age undergoing IVF or ICSI treatment; 2) Studies investigating the composition or diversity of the reproductive tract (vaginal, cervical, or endometrial) or follicular fluid microbiota, or assessing the effect of probiotic interventions on IVF/ICSI pregnancy outcomes; 3) Studies reporting at least one pregnancy-related outcome, such as embryo implantation rate, clinical pregnancy rate, live birth rate, or miscarriage rate; 4) Original peer-reviewed human studies, including RCTs, cohort studies, and case–control studies.

Exclusion criteria: 1) Animal experiments or in vitro studies; 2) Studies involving populations not undergoing IVF/ICSI; 3) Studies lacking explicit microbiota data or pregnancy outcome information; 4) Reviews, commentaries, case reports, conference abstracts, and non-English publications.

### Study selection

After de-duplication using reference management software, two reviewers (RC and RZ) independently screened titles and abstracts to exclude studies that did not meet the inclusion criteria. The remaining articles were then assessed in full text to determine eligibility for inclusion. Any discrepancies between reviewers were resolved through discussion or, if necessary, by adjudication by a third reviewer (GM). The study selection and inclusion process was conducted in accordance with the PRISMA 2020 flow diagram, with the number of studies included and excluded at each stage, as well as reasons for exclusion, systematically recorded.

### Data extraction

Two reviewers (BH and JH) independently extracted data using a predesigned standardized data extraction form. The following data were collected: 1) Study characteristics: first author, year, country, and study design; 2) Participant characteristics: sample size, mean age, and IVF/ICSI protocol details; 3) Microbiota assessment: sampling sites and detection methods (e.g., 16S rRNA sequencing, qPCR, or culture); 4) Microbiological outcomes: alpha and beta diversity, dominant genera/species, and relative abundance of *Lactobacillus*; 5) Probiotic interventions (if applicable): strain, administration route, dosage, and duration; 6) Pregnancy outcomes: embryo implantation rate, clinical pregnancy rate, live birth rate, and other reported reproductive outcomes. All extracted data were independently cross-checked by two reviewers (RG and LQ) to ensure accuracy, completeness, and consistency. Any discrepancies were resolved through discussion or, if necessary, consultation with a third reviewer.

Given the inherently low microbial biomass of endometrial and follicular fluid samples, we systematically assessed contamination control measures across included studies, including negative controls, extraction blanks, sterile sampling procedures, and computational decontamination pipelines. Studies lacking these safeguards were considered at higher risk of contamination in qualitative synthesis and evidence grading, improving the interpretability of the review findings (Supplementary Table 2).

### Quality assessment

The methodological quality of included studies was independently assessed by two reviewers (RG and GM), with disagreements resolved by discussion or adjudication by a third reviewer (JH). Validated, design-specific tools were applied: randomized controlled trials were assessed using the Cochrane Risk of Bias 2.0 (RoB 2.0) tool; non-randomized interventional studies using ROBINS-I; and observational studies (prospective and retrospective cohort, case–control, and cross-sectional) using the Joanna Briggs Institute (JBI) critical appraisal tools.

Risk of bias was evaluated at the domain level according to each tool’s guidance, and an overall judgment was assigned as Low, Moderate, High, or Unclear. To enable comparability across study designs, RoB 2.0 ratings of “Some concerns” were categorized as Moderate, ROBINS-I Serious or Critical ratings as High, and JBI-based assessments were summarized according to predefined thresholds based on the proportion of affirmative responses.

### Data synthesis

Due to variations in study design, microbiota assessment methods, and definitions of pregnancy outcomes among the included studies, a qualitative synthesis was performed in this review. Study findings were categorized according to microbiota type, differences in abundance, and pregnancy outcomes, and descriptive analyses were used to explore potential associations. In addition, the effects and possible mechanisms of probiotic interventions were identified and summarized.

## Results

### Study selection

**Fig. 1:**
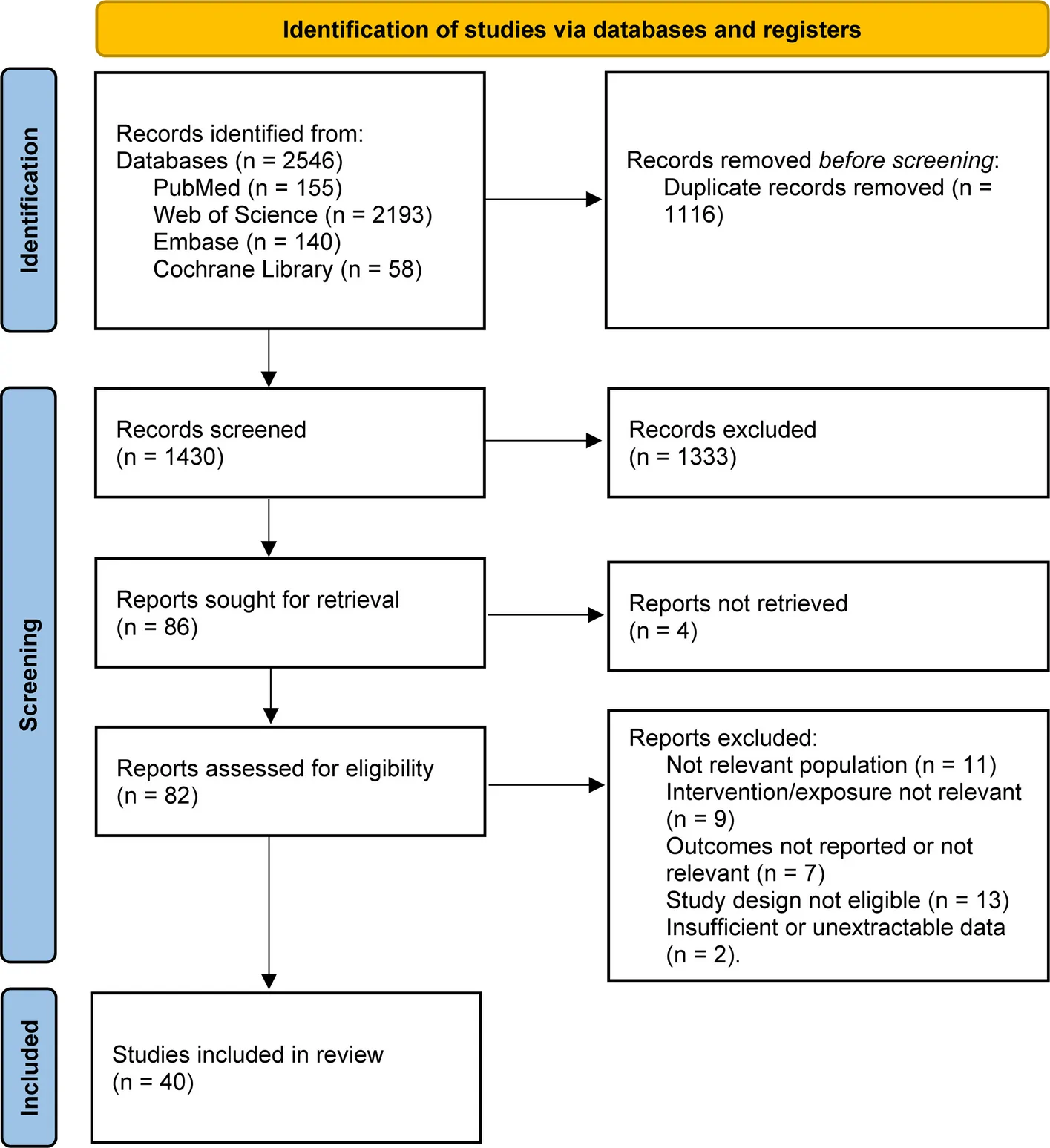
PRISMA flow diagram illustrating the literature search, screening, and study selection process for this systematic review. *PRISMA*, Preferred Reporting Items for Systematic reviews and Meta-Analyses

A total of 2,546 records were identified through database searches (PubMed, *n =* 155; Web of Science, *n =* 2,193; Embase, *n =* 140; Cochrane Library, *n =* 58). After removal of 1,116 duplicates, 1,430 unique records underwent title and abstract screening, of which 1,333 were excluded. Eighty-six full-text articles were assessed for eligibility, and 40 studies were included in the final qualitative synthesis. The study selection process is summarized in the PRISMA 2020 flow diagram (Fig. 1).

### Study characteristics

Of the 40 studies included (Table 1), two were RCTs [38, 39], 28 were prospective cohort studies, including pilot and observational cohorts [3, 10, 11, 20, 25, 32, 34, 40–60], five were retrospective cohort studies [61–65], three were cross-sectional studies [66–68], two were case–control studies [24, 69]. Probiotic interventions were investigated in five studies, administered orally or intravaginally using *Lactobacillus* or multi-strain formulations [38, 39, 45, 56, 63].

**Table 1:** Summary of included studies investigating reproductive tract and follicular fluid microbiota in relation to IVF/ICSI pregnancy outcomes

Author (Year)	Country	Study design	Sample size	ART	Participants	Specimen type	Microbiota assessment method	Probiotic intervention (if applicable)
Gilboa et al., 2005 [38]	Israel	RCT	117 (50 probiotic, 67 control)	IVF	Women < 38 y, undergoing IVF, normal uterine cavity, no hydrosalpinges, no chronic illness, ≤ 3 previous IVF failures	Vaginal swabs	Culture and Gram staining (*Lactobacillus* prevalence)	Intravaginal probiotics immediately after oocyte retrieval (Probiotic Femina; 2 capsules, each containing 3 × 10⁹ live cells of *Lactobacillus acidophilus*, *Bifidobacterium bifidum*, and *Bifidobacterium longum*); allocation pseudo-randomized by week of oocyte retrieval
Pelzer et al., 2013 [34]	Australia	Prospective cohort study	263	IVF	Women undergoing IVF with idiopathic, endometriosis, or polycystic ovary syndrome	Vaginal swabs, Follicular fluid	Culture-based methods, differential media, colony-forming unit count	None
Haahr et al., 2016 [40]	Denmark	Prospective cohort study	130	IVF	Women undergoing IVF; excluded if antibiotics within 1 month or vaginal sample > 2 months before ET	Vaginal swabs	Gram staining with Nugent scoring; qPCR for *Gardnerella vaginalis*, *Atopobium vaginae*, and *Lactobacillus* spp.	None
Moreno et al., 2016 [41]	Spain	Prospective pilot studies (3 separate)	13 (pilot 1, paired EF and VA), 22 (pilot 2, hormonal regulation), 35 (pilot 3, IVF outcome)	IVF	Fertile women (pilots 1 & 2), infertile women undergoing IVF (pilot 3)	Endometrial fluid, vaginal aspirates	16S rRNA sequencing	None
Moini et al., 2018 [42]	Iran	Prospective cohort study	408	ICSI	Women with male factor infertility undergoing ICSI	Vaginal samples	Gram staining and Nugent scoring for bacterial vaginosis (*Gardnerella vaginalis*, *Mycoplasma*, and other morphotypes)	None
Kyono et al., 2019 [43]	Japan	Prospective pilot study	92 (117 tested)	IVF	Women < 45 y, no symptomatic vaginitis/endometritis	Endometrial fluid	Varinos endometrial flora test (bacterial profiling; percentage *Lactobacilli* reported)	Trial antibiotics + lactoferrin + probiotics for dysbiosis
Hashimoto & Kyono, 2019 [20]	Japan	Prospective observational study	99	IVF (FET)	Women < 40 years old undergoing embryo transfer	Endometrial fluid	16S rRNA sequencing	None
Bernabeu et al., 2019 [24]	Spain	Prospective pilot case–control study	31	IVF/ICSI	Women undergoing ART with cryotransfer of a single euploid blastocyst	Vaginal fluid	16S rRNA sequencing	None
Koedooder et al. 2019 [10]	Netherlands/Germany	Prospective cohort study	192 (50 external validation)	IVF/IVF-ICSI	Subfertile women 20–44 y, first IVF/IVF-ICSI cycle	Vaginal swab taken	16S-23S rRNA interspace length; CST assignment	None
Vergaro et al. 2019 [44]	Spain	Prospective cohort study	150	IVF/ICSI	Caucasian women undergoing IVF/ICSI with donated oocytes	Vaginal fluid	qPCR for *Lactobacillus* spp., *Gardnerella vaginalis*, *Atopobium vaginae*, *Mycoplasma hominis*, *Prevotella* spp.	None
Iwami et al., 2020 [45]	Japan	Prospective cohort study	527	IVF	Women < 42 y with RIF (≥ 3 failed ETs) or RPL (≥ 2 miscarriages)	Endometrial tissue (aspiration, day 15–25 of cycle)	NGS-based EMMA (16S rRNA sequencing, *Lactobacillus* focus)	Mild/ultralow EMMA group: probiotics; Normal/Abnormal: no probiotics reported
Riganelli et al., 2020 [46]	Italy	Pilot prospective cohort study	34	IVF	Infertile women with unsuccessful implantation attempts	Vaginal fluid, endometrial biopsy	16S rRNA sequencing	None
Kong et al., 2020 [61]	China	Retrospective cohort study	555	IVF	Women 20–38 y, tubal or other infertility	Vaginal secretion	16S rRNA sequencing	None
Hao et al., 2021 [69]	China	Case–control study	100	IVF	Women 20–40 y, various infertility causes	Cervical mucus	16S rRNA sequencing	None
Diaz-Martínez et al., 2021 [47]	Spain	Pilot prospective cohort study	48	IVF/ICSI	Women 18–50 y undergoing FET with/without RIF	Vaginal swabs and endometrial sample	16S rRNA sequencing	None
Usman et al., 2021 [48]	Nigeria	Prospective cohort study	86 completed (90 enrolled)	IVF	Women 18–38 y undergoing IVF-ET at a tertiary center; normal cycles and uterine	Follicular fluid	Gram stain + aerobic/yeast cultures with biochemical identification	None
Zeng et al., 2021 [62]	China	Retrospective cohort study	2285	IVF	Women first fresh autologous IVF cycle, no comorbidities or prior ART failure	Vaginal swabs	Gram staining, microscopy, Nugent scoring	None
Wang et al. 2021 [3]	China	Prospective cohort study	150	IVF/ICSI	Women undergoing first fresh IVF-ET	Vaginal and cervical samples	16S rRNA sequencing	None
Karaer et al. 2021 [49]	Turkey	Prospective cohort study	223	IVF/ICSI	Women 23–39 y, BMI 20–29.9, excluded systemic disease, genital infection, recent antibiotics/probiotics	Vaginal swabs	16S rRNA sequencing	None
Kim et al. 2022 [32]	South Korea	Prospective observational study	49	IVF	Women < 40 y, FSH < 10 mIU/mL, BMI < 30 kg/m^2^, various infertility	Vaginal swabs, follicular fluid	Aerobic and anaerobic microbial culture	None
Ji et al., 2022 [50]	China	Prospective cohort study	229 (275 FET cycles)	FET	Women undergoing frozen embryo transfer	Vaginal swabs	Gram staining, Nugent scoring	None
Villani et al., 2022 [51]	Italy	Prospective cohort study	88	IVF	Women undergoing ART protocols, various infertility types	Cervical swabs	16S rRNA sequencing	None
Moreno et al., 2022 [52]	Multi-country (Europe, America, Asia)	Prospective observational study	342	IVF	Infertile women ≤ 40 y (IVF) or ≤ 50 y (ovum donation)	Endometrial fluid and biopsy samples	16S rRNA sequencing	None
Di Pierro et al. 2023 [63]	Italy	Retrospective controlled observational study	160 (80 probiotic, 80 control)	IVF/ICSI	Women 18–45 y, primary infertility, no previous pregnancy	Vaginal swabs	16S rRNA sequencing	Oral *Lactobacillus crispatus* M247
Ge et al., 2023 [64]	China	Retrospective observational study	107	IUI/IVF/ICSI	Women 22–50 y	Vaginal secretions	Multiplex quantitative fluorescent PCR for 22 vaginal microbes (*Lactobacillus crispatus*, *Lactobacillus iners*, *Gardnerella vaginalis*, *Prevotella species*, *Ureaplasma urealyticum*, and others.)	None
Tong et al., 2023 [53]	China	Prospective cohort study	19 (IVF)/6 (Natural)	IVF/Natural	Women from Northern China	Vaginal swabs	16S rRNA sequencing	None
Miyagi et al., 2023 [54]	Japan	Prospective cohort study	35 (vaginal 34, endometrial 33)	IVF/ICSI/FET	Infertile women undergoing good-quality embryo transfer	Vaginal and endometrial secretions	16S rRNA sequencing	None
Cariati et al., 2023 [55]	Italy	Prospective cohort study	93	IVF	Women 29–47 y, good-quality blastocysts	Endometrial fluid	Culturomics-based microbial culture analysis; identification by MALDI-TOF MS	None
Guan et al., 2023 [66]	China	Cross-sectional study	120	IVF/FET	Women aged 20–40 years, undergoing FET	Cervical samples	16S rDNA full-length sequencing (16S-FAST)	None
Kadogami et al., 2023 [65]	Japan	Retrospective observational study	151	IVF	Women with RIF (≥ 3 transfers of good-quality blastocysts)	Endometrial tissue	16S rRNA sequencing	None
Koort et al., 2023 [67]	Estonia	Cross-sectional study	97 ART couples, 12 healthy couples	IVF/ICSI	Infertile couples (≥ 1 year infertility), 12 healthy controls	Vaginal fluid (women), Semen (men)	16S rRNA sequencing; Gram stain and Nugent BV	None
Väinämö et al., 2023 [25]	Finland	Prospective longitudinal cohort study	76	IVF	Subfertile women < 40 y, BMI < 35	Vaginal swabs	16S rRNA sequencing	None
Bui et al., 2023 [60]	Netherlands	Prospective cohort study	141	IVF	Women with unexplained or male factor infertility, failed one IVF/ICSI	Endometrial biopsies	16S rRNA sequencing	None
Faisal et al. 2023 [68]	Iraq	Cross-sectional study	46	ICSI	Infertile women (asymptomatic vaginal infections)	Vaginal samples	Microbiological culture	None
Iwami et al. 2023 [56]	Japan	Prospective cohort study	122 (61 study, 61 control)	IVF/FET	Women < 40 y, RIF (≥ 3 failed ETs), no intrauterine lesions, no recent antibiotics	Endometrial biopsies	16S rRNA sequencing	Antibiotic and/or probiotic (InVag® or Lactoflora®) tailored according to EMMA before FET
Nishio et al., 2024 [57]	Japan	Prospective observational cohort study	23	IVF	Women undergoing IVF-ET; pregnancy classified post-ET	Cervicovaginal mucus	16S rRNA sequencing	None
Wu et al., 2025 [58]	China	Prospective cohort study	131	IVF	Women 20–35 y, first or second IVF/FET, no major risk factors	Cervical mucus	16S rDNA full-length sequencing (16S-FAST)	None
Bar et al., 2025 [11]	USA	Prospective cohort study	30 enrolled, 28 analyzed	IVF	Women with unexplained or male factor infertility	Vaginal swabs	16S rRNA sequencing	None
Huerga López et al., 2025 [39]	Spain	RCT	70 randomized; 57 completed (placebo 30, probiotic 27)	IVF	Couples with unexplained infertility, both partners enrolled	Vaginal swabs, semen	Culture, MALDI-TOF, 16S rRNA sequencing	Oral *Ligilactobacillus salivarius* CECT5713 (3 × 10⁹ CFU/day per partner) vs placebo; 6 months prior to IVF and continued up to 12 weeks pregnancy
Alley et al. 2025 [59]	USA	Prospective observational cohort	87	FET after IVF/ICSI	Adults ≥ 18 y scheduled for FET; diverse cohort	Vaginal swabs	16S rRNA sequencing	None

*Abbreviations*: *ART* Assisted reproductive technology, *BMI* Body mass index, *BV* Bacterial vaginosis, *CFU* Colony forming units, *CST* Community state type, *EMMA* Endometrial microbiome metagenomic analysis, *ET* Embryo transfer, *EF* Endometrial fluid, *FET* Frozen embryo transfer, *IVF* In vitro fertilization, *IVF-ET* In vitro fertilization-embryo transfer, *IUI* Intrauterine insemination, *ICSI* Intracytoplasmic sperm injection, *NGS* Next-generation sequencing, *qPCR* quantitative polymerase chain reaction, *RCT* Randomized controlled trial, *RIF* Recurrent implantation failure, *RPL* Recurrent pregnancy loss, *16S rRNA sequencing* sequencing of the 16S ribosomal RNA gene, 16S rDNA full-length sequencing (16S-FAST), full-length 16S rRNA gene sequencing, *VA* Vaginal aspirate or swab, *IVF* In vitro fertilization, *MALDI-TOF MS* Matrix-assisted laser desorption/ionization time-of-flight mass spectrometry

Participants were women aged 18–50 years undergoing IVF, ICSI, or frozen embryo transfer for various infertility causes, including unexplained infertility, male factor infertility, recurrent implantation failure, recurrent pregnancy loss, endometriosis, polycystic ovary syndrome, and tubal factor infertility (Table 1).

The microbiota was assessed using diverse methodologies (Table 1). The primary approach was 16S rRNA sequencing, targeting specific regions or full-length (16S-FAST) [3, 10, 11, 20, 24, 25, 39, 41, 45–47, 49, 51–54, 56–61, 63, 65–67, 69]. Several studies complemented sequencing with qPCR to detect key microbial species, including *Lactobacillus* spp., *Gardnerella* vaginalis, *Atopobium vaginae*, *Mycoplasma hominis*, and *Prevotella* spp. [40, 44].

Gram staining with Nugent scoring was commonly used to classify bacterial vaginosis (BV) [38, 40, 42, 48, 50, 62, 67], while culture-based methods, sometimes combined with MALDI-TOF MS, were applied for live bacterial identification [32, 34, 38, 39, 48, 55, 68]. Multiplex qPCR allowed simultaneous detection of multiple vaginal microbes [64], and specialized assays, such as the Varinos endometrial flora test [43] and the Endometrial Microbiome Metagenomic Analysis (EMMA) assay [45, 56], focused on *Lactobacillus*-dominated communities.

Samples were collected from multiple reproductive tract sites and follicular fluid (Table 1). Vaginal swabs or aspirates were the most common, followed by cervical mucus, endometrial fluid or tissue obtained via aspiration or biopsy.

### Risk of bias in studies

Risk of bias was independently assessed for all included studies using the RoB 2.0 tool (RCTs), ROBINS-I tool (non-randomized interventional studies), or JBI checklists (observational studies), as appropriate. Overall risk-of-bias judgments are summarized in Table 2. Detailed per-domain assessments for all studies are provided in Supplementary Tables 3.

**Table 2:** Overall risk of bias summary for all included studies

Author (Year)	Study type	Overall risk of bias
Gilboa et al., 2005 [38]	RCT	Low
Huerga López et al., 2025 [39]	RCT	Moderate
Kyono et al., 2019 [43]	Non-randomized interventional	High
Di Pierro et al. 2023 [63]	Non-randomized interventional	High
Iwami et al. 2023 [56]	Non-randomized interventional	Moderate
Bernabeu et al., 2019 [24]	Case–control	Moderate
Hao et al., 2021 [69]	Case–control	Low
Koort et al., 2023 [67]	Cross-sectional	Moderate
Faisal et al. 2023 [68]	Cross-sectional	Moderate
Guan et al., 2023 [66]	Cross-sectional	Moderate
Pelzer et al., 2013 [34]	Cohort	Moderate
Haahr et al., 2016 [40]	Cohort	Moderate
Moreno et al., 2016 [41]	Cohort	Low
Moini et al., 2018 [42]	Cohort	Moderate
Hashimoto & Kyono, 2019 [20]	Cohort	Moderate
Koedooder et al. 2019 [10]	Cohort	Moderate
Vergaro et al. 2019 [44]	Cohort	Moderate
Iwami et al., 2020 [45]	Cohort	High
Riganelli et al., 2020 [46]	Cohort	Moderate
Kong et al., 2020 [61]	Cohort	Moderate
Diaz-Martínez et al., 2021 [47]	Cohort	Moderate
Usman et al., 2021 [48]	Cohort	Low
Zeng et al., 2021 [62]	Cohort	Moderate
Wang et al. 2021 [3]	Cohort	High
Karaer et al. 2021 [49]	Cohort	Moderate
Kim et al. 2022 [32]	Cohort	Low
Ji et al., 2022 [50]	Cohort	Moderate
Villani et al., 2022 [51]	Cohort	Moderate
Moreno et al., 2022 [52]	Cohort	Moderate
Ge et al., 2023 [64]	Cohort	Moderate
Tong et al., 2023 [53]	Cohort	High
Miyagi et al., 2023 [54]	Cohort	Moderate
Cariati et al., 2023 [55]	Cohort	High
Kadogami et al., 2023 [65]	Cohort	Moderate
Väinämö et al., 2023 [25]	Cohort	Moderate
Bui et al., 2023 [60]	Cohort	Moderate
Nishio et al., 2024 [57]	Cohort	Moderate
Wu et al., 2025 [58]	Cohort	Moderate
Bar et al., 2025 [11]	Cohort	Moderate
Alley et al. 2025 [59]	Cohort	Moderate

*RCT* Randomized controlled trial, *Non-randomized interventional* Non-randomized interventional study, *Case–control* Case–control study, *Cross-sectional* Cross-sectional study, *Cohort* Cohort study. Overall risk of bias was rated as Low, Moderate, or High. Risk-of-bias tools were applied according to study type: RoB 2.0 for RCTs, ROBINS-I for non-randomized interventional studies, and JBI checklists for observational studies

No studies were excluded based on the risk of bias (Tables 2). Among the RCTs, Gilboa et al. [38] was rated as having a low overall risk of bias, whereas Huerga López et al. [39] was judged to have a moderate risk, mainly due to selective reporting and missing outcome data.

For non-randomized interventional studies, overall methodological quality was generally moderate or high. One study was rated as moderate risk [56], and two studies were judged as high risk [43, 63].

For case–control studies, one study was rated as low risk [69], and one study was moderate risk [24]. All cross-sectional studies were assessed as having a moderate risk of bias [66–68].

Among cohort studies, the majority were classified as moderate risk [10, 20, 25, 32, 34, 40–54, 58–60, 62, 64, 65], four studies were judged as high risk [3, 45, 53, 55], and one study was rated as low risk [48].

The most frequent sources of bias were confounding, intervention classification, deviations from intended interventions, and outcome measurement. These reflect methodological heterogeneity, variable microbial assessment techniques, and inconsistent definitions of reproductive outcomes. Other limitations included small sample sizes, non-standardized sequencing targets, and differing criteria for implantation, clinical pregnancy, and live birth.

### Overview of contamination controls

Among the 40 studies included in this review, reproductive tract samples included endometrial tissue or fluid, cervical mucus, follicular fluid, and vaginal swabs, several of which represent low-biomass sample types. Contamination safeguards assessed across studies included negative (no-template) controls, extraction blanks, sterile sampling procedures, and computational decontamination pipelines (Supplementary Table 2).

Overall, 11 studies reported at least one negative control, and 6 reported extraction blanks. A total of 28 studies applied computational decontamination pipelines, whereas most studies reported using sterile kits or glove changes during sampling and processing. Only five studies implemented comprehensive safeguards across all four measures, representing the lowest risk of contamination. The frequent absence of negative controls and extraction blanks highlights that contamination remains a major methodological concern in low-biomass reproductive tract microbiota research.

### Methodological characteristics of included microbiome studies

The laboratory and bioinformatic methodologies across included studies are summarized in Supplementary Table 4. Considerable heterogeneity was observed in sample collection and storage, DNA extraction protocols, sequencing strategies, and statistical analysis approaches.

### Reproductive tract microbiota

#### *Lactobacillus*-dominant vs non-dominant communities

**Fig. 2:**
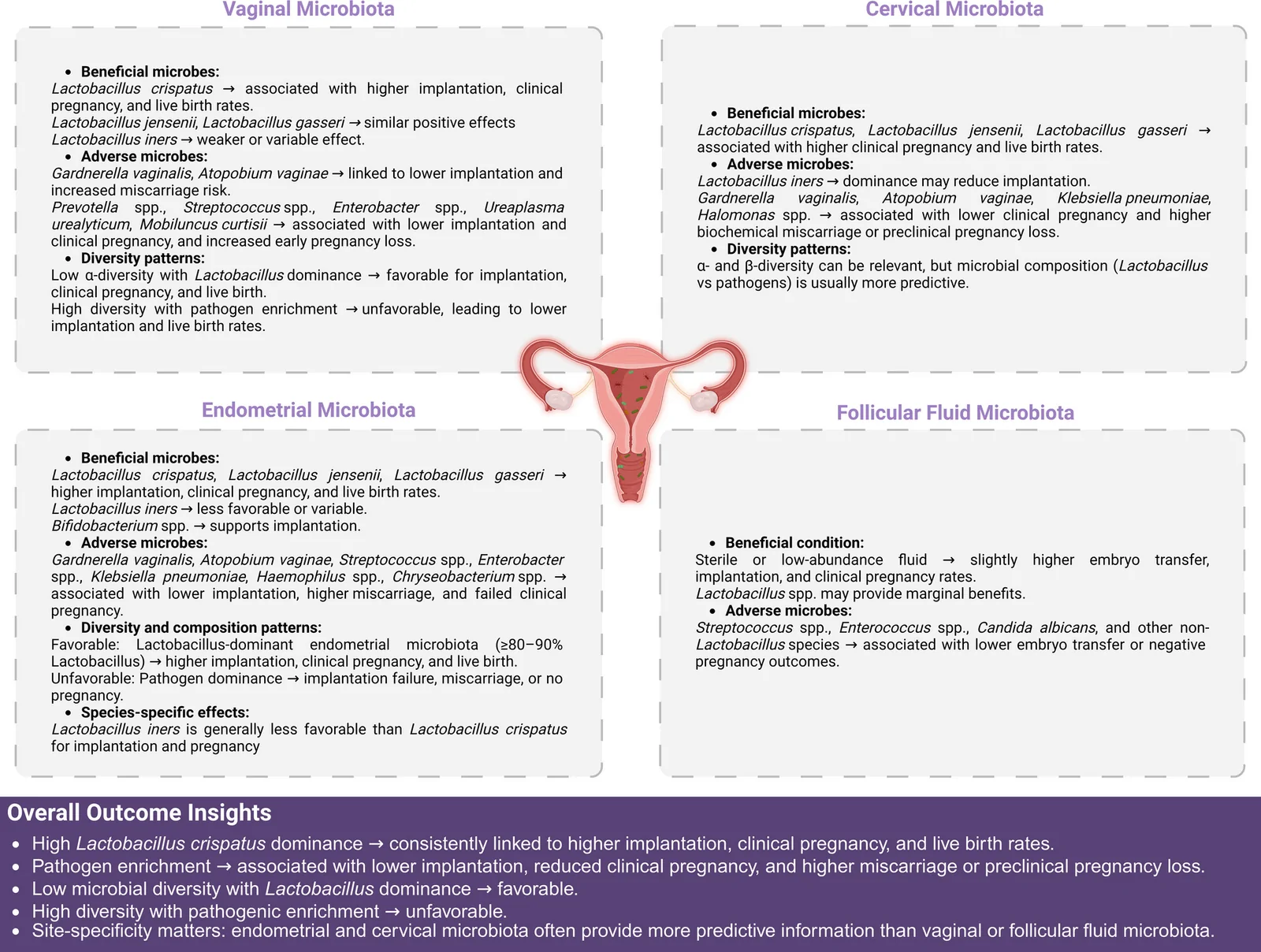
Reproductive tract microbiota and ART outcomes. It should be noted that Vaginal dysbiosis, such as BV, primarily affects early pregnancy, with less consistent effects at later stages. Cervical microbiota profiles are often more predictive of ART outcomes than vaginal profiles alone, as pathogen enrichment can reduce ART success even in the presence of *Lactobacillus*. In contrast, endometrial microbiota α-diversity alone is not strongly predictive; specific microbial compositions appear to play a more decisive role. Endometrial and vaginal microbiota do not always reflect each other, so vaginal profiles cannot fully represent endometrial conditions. Patients with non-*Lactobacillus*-dominant microbiota may still achieve pregnancy, but their success rates are generally lower. Overall, endometrial and cervical microbiota seem more critical for ART success, whereas follicular fluid microbiota shows limited predictive value. This figure was created with BioRender.com

The microbiota of the female reproductive tract, including the vaginal, cervical, and endometrial niches, and to a lesser extent the follicular microbiota within the ovarian microenvironment, has been investigated in relation to IVF/ICSI outcomes (Table 3 and Fig. 2). Across studies, *Lactobacillus*-dominant communities, particularly those enriched with *Lactobacillus crispatus*, were frequently associated with higher implantation, clinical pregnancy, and live birth rates. Larger observational studies report that increased endometrial *Lactobacillus* abundance correlated with live birth, whereas dysbiotic profiles enriched with pathogenic taxa, including *Gardnerella* and other anaerobic bacteria, were more frequently observed in women with implantation failure or miscarriage [52]. Cervical microbiota dominated by *Lactobacillus crispatus* (Community Microbiota Type 1, CMT1) was associated with higher biochemical and clinical pregnancy rates than *Lactobacillus iners*-dominant (CMT2) or diverse communities (CMT3) [66]. Vaginal *Lactobacillus* dominance was also associated with higher clinical pregnancy and live birth rates in several observational studies [11, 24, 25, 59], whereas *Lactobacillus iners* dominance appeared less consistently associated with favorable implantation and live birth outcomes [60, 65]. Early pilot studies suggested similar trends in endometrial microbiota profiles [41]; however, these findings were derived from small exploratory cohorts, including a substudy of only 35 participants, and should therefore be interpreted cautiously. The reported implantation rate difference (60.7% vs 23.1%) may represent hypothesis-generating evidence rather than definitive confirmation of a causal microbial effect.

**Table 3:** Summary of reproductive tract microbiota findings in IVF/ICSI pregnancy outcomes

Author (Year)	Sample type	ART	Pregnancy outcomes	Microbiota change related to pregnancy outcomes	Microbial diversity analysis results
Haahr et al., 2016 [40]	Vaginal swabs	IVF	Biochemical pregnancy 45%; Clinical pregnancy 35%	AVM, defined as the presence of *Gardnerella vaginalis* and/or *Atopobium vaginae* above qPCR thresholds, and bacterial vaginosis (Nugent score 7–10), was associated with lower clinical pregnancy rates. Intermediate flora showed variable associations	N/A
Moreno et al., 2016 [41]	Endometrial fluid, vaginal aspirates	IVF	Implantation rate: 60.7% (LD) vs 23.1% (NLD); Pregnancy rate: 70.6% (LD) vs 33.3% (NLD); Ongoing pregnancy rate: 58.8% (LD) vs 13.3% (NLD); Live birth rate: 58.8% (LD) vs 6.7% (NLD); Miscarriage rate: 16.7% (LD) vs 60% (NLD)	Endometrial fluid microbiota was classified as LD (≥ 90% *Lactobacillus* OTUs) or NLD (< 90% *Lactobacillus* OTUs). NLD microbiota exhibited higher relative abundance of *Gardnerella* and *Streptococcus*, both of which were associated with poorer reproductive outcomes. Vaginal microbiota differed from endometrial fluid microbiota in approximately 20% of subjects	NLD group showed higher Shannon diversity compared to LD. Overall alpha diversity did not correlate directly with IVF outcomes
Moini et al., 2018 [42]	Vaginal samples	ICSI	Implantation rate: BV higher than Normal (*P* = 0.02); Chemical pregnancy rate: BV 60%, Normal 39.1%, Intermediate 41.8%; Early miscarriage: BV 26.7%, Normal 15%, Intermediate 17.4%; Live birth: no significant difference	Vaginal microbiota was classified by Nugent score: BV (7–10), Intermediate (4–6), Normal (0–3); BV prevalence was 7.3%, Normal 75.2%, Intermediate 17.5%. BV was strongly associated with lower implantation rates, higher miscarriage rates, and reduced live birth rates	N/A
Kyono et al., 2019 [43]	Endometrial fluid	IVF	Pregnancy rate per patient: LDM 58.9%, NLDM 47.2%; Miscarriage rate: LDM 24.2%, NLDM 17.6%	LDM was observed in 51.1% of patients; NLDM in 48.9%. Dominant NLDM taxa included *Atopobium*, *Bifidobacterium*, *Gardnerella*, *Megasphaera*, *Sneathia*, *Prevotella*, *Staphylococcus*, and *Streptococcus*	N/A
Hashimoto & Kyono, 2019 [20]	Endometrial fluid	IVF	Clinical pregnancy per transfer: eubiotic 52.9%, dysbiotic 54.8%; Miscarriage: 11.1% vs 5.9% (not significant)	Eubiosis was defined as ≥ 80% *Lactobacillus* + *Bifidobacterium*; dysbiosis was defined as < 80% with increased *Atopobium*, *Gardnerella*, and *Streptococcus*	N/A
Bernabeu et al., 2019 [24]	Vaginal fluid	IVF/ICSI	Pregnancy rate: 54.8% achieved pregnancy, 45.2% did not	Women who achieved pregnancy had a greater presence of *Lactobacillus* spp. *Lactobacillus*-dominated microbiota was associated with successful pregnancy	α-diversity was higher in non-pregnant patients, though not significantly (*P* = 0.088). β-diversity showed no significant difference between pregnancy groups
Koedooder et al. 2019 [10]	Vaginal swabs	IVF/ICSI	Overall pregnancy ≈35%; low-chance subgroup 6%	Women with an unfavorable microbiome profile-characterized by low *Lactobacillus* (< 20%), high *Lactobacillus jensenii* (> 35%), presence of *Gardnerella vaginalis* IS-pro type 1, or high Proteobacteria (> 28%)-were less likely to achieve pregnancy	N/A
Vergaro et al. 2019 [44]	Vaginal swabs	IVF	Clinical pregnancy rate: Normal microbiota 44% vs Abnormal vaginal microbiota 9% (*P* = 0.004)	AVM defined by high concentrations of *Gardnerella vaginalis* and *Atopobium vaginae*; *Lactobacillus*-dominated in 72% of normal microbiota samples	N/A
Riganelli et al., 2020 [46]	Vaginal swabs, Endometrial biopsy	IVF	Implantation failure and lower live birth rates associated with non-*Lactobacillus*-dominant microbiota	*Lactobacillus* spp. predominated in successful pregnancies (*Lactobacillus iners*, *Lactobacillus crispatus*); *Gardnerella vaginalis*, *Escherichia fergusonii*, *Streptococcus dentisani* more abundant in failure cases	PCoA and PLS-DA applied to endometrium showed no significant differences between pregnant and non-pregnant women
Kong et al., 2020 [61]	Vaginal secretion	IVF	Clinical pregnancy: 238/475 (50.1%)	Pregnant patients had higher abundance of *Lactobacillus* and lower abundance of *Gardnerella*, *Atopobium*, and *Prevotella*. At phylum level, *Firmicutes* and *Proteobacteria* were higher, while *Actinobacteria*, *Fusobacteria*, and *Bacteroidetes* were lower in pregnant patients	α-diversity (Shannon and Simpson indices) was higher in non-pregnant patients compared with pregnant patients
Hao et al., 2021 [69]	Cervical mucus	IVF	Clinical pregnancy rate: Fresh IVF-ET 25/51 (49%); Frozen-thawed IVF-ET 27/49 (55%)	In the successful pregnancy group, *Lactobacillus*, *Akkermansia*, *Desulfovibrio*, *Atopobium*, and *Gardnerella* showed higher abundance compared to non-pregnant women. *Lactobacillus* was negatively correlated with the other genera	α-diversity: Higher in the pregnancy group (*P* = 0.0078) in fresh IVF-ET cycles; β-diversity: Significant difference in fresh IVF-ET cycles (R = 0.242, *P* = 0.001) and frozen-thawed cycles (R = 0.062, *P* = 0.045)
Diaz-Martínez et al., 2021 [47]	Vaginal swabs and endometrial sample	IVF/ICSI	β-hCG positive 54.2% (26/48); Clinical pregnancy 43.8% (21/48); Biochemical miscarriage 10.4% (5/48); Clinical miscarriage 14.3% (7/48)	Pregnant women had higher vaginal *Lactobacillus* abundance at embryo-transfer day (97.69% vs 94.63%; *P* = 0.027); non-pregnant women had higher *Streptococcus* and *Prevotella*. No significant taxonomic differences were observed in endometrial samples by pregnancy outcome	α and β diversity of vaginal and endometrial samples were not statistically different between pregnant and non-pregnant women
Zeng et al., 2021 [62]	Vaginal swabs	IVF	Live birth rate significantly lower in NLD group (31.83%) vs LD group (41.22%)	Non-dominance of *Lactobacillus* in vaginal microbiota associated with reduced live birth rates, increased preclinical pregnancy loss, and higher preterm birth rates	N/A
Wang et al. 2021 [3]	Vaginal and cervical samples	IVF/ICSI	89 pregnant, 61 non-pregnant	Microbial community of both vaginal and cervical microbiota was not significantly different between pregnant and non-pregnant groups	Shannon indices of vaginal and cervical microbiota were not significantly different; linear discriminant analysis, random forest analysis, and ROC curve analysis indicated that *Bifidobacterium*, *Prevotella*, and *Lactobacillus iners* in the vagina, as well as *Solanum torvum*, *Fusobacterium*, and *Streptococcus* in the cervix, may be negatively associated with clinical pregnancy after IVF
Karaer et al. 2021 [49]	Vaginal swabs	IVF/ICSI	108 pregnant, 115 non-pregnant	Non-pregnant group showed lower *Lactobacillus* (67.71%) vs pregnant (79.72%, *P* = 0.06) and higher *Streptococcus* (7.81% vs 2.28%, *P* = 0.014) and *Gardnerella* (9.40% vs 5.56%). LEfSe identified *Streptococcus* as the top discriminator (LDA > 4.0)	α diversity (Observed OTUs, Chao1, Shannon) showed no significant differences. β diversity (weighted & unweighted UniFrac PCoA) showed no clear separation
Ji et al., 2022 [50]	Vaginal swabs	FET	Implantation rate: Normal 66.9%, Mild dysbiosis 45.5%, Severe dysbiosis 29.6%; Clinical pregnancy rate: Normal 84.3%, Mild dysbiosis 57.3%, Severe dysbiosis 34.2%; Ongoing pregnancy rate: Normal 83.1%, Mild dysbiosis 49.3%, Severe dysbiosis 27%; Preclinical pregnancy loss: Normal 1.3%, Mild 20.4%, Severe 25.5%; Miscarriage: Normal 1.3%, Mild 14%, Severe 21.1%	*Lactobacillus*-dominant vaginal microbiota in normal group; dysbiosis in severe group	N/A
Villani et al., 2022 [51]	Cervical swabs	IVF	Favorable 39, Unfavorable 49	Increased abundance of *Atopobium vaginae* in the unfavorable group; increased *Lactobacillus* species in the favorable group	PELORA analysis identified bacterial clusters with significant differences between favorable and unfavorable groups. Clusters with *Lactobacillus crispatus* and *Lactobacillus iners* associated with favorable outcomes
Moreno et al., 2022 [52]	Endometrial fluid and biopsy samples	IVF	Live birth 41.2%, Biochemical pregnancy 7.9%, Clinical miscarriage 8.2%, No pregnancy 42.1%	Dysbiotic microbiota: increased *Gardnerella*, *Haemophilus*, *Klebsiella*, *Streptococcus*, *Atopobium*, *Bifidobacterium*, *Chryseobacterium* associated with no pregnancy and clinical miscarriage. Increased *Lactobacillus* associated with live birth	Z-score analysis of *Lactobacillus* abundance: live birth associated with higher *Lactobacillus* abundance; no pregnancy and clinical miscarriage had higher abundance of pathogenic bacteria
Kim et al. 2022 [32]	Follicular fluid and vaginal swabs	IVF/ICSI	Clinical pregnancy rate: Day 3 embryo transfer 12.5% (follicular fluid positive) vs 28.0% (negative), Day 5 embryo transfer 0% vs 62.5%	Common microorganisms: *alpha-streptococcus*, *Kocuria kristinae*, *Enterococcus faecalis*, *Streptococcus agalactiae*, *Enterococcus coli*, *Coagulase-negative staphylococci*, *Candida albicans*	N/A
Ge et al., 2023 [64]	Vaginal secretions	IUI/IVF/ICSI	Clinical pregnancy rate: IUI/IVF/ICSI 53.66% (normal microecology) vs 37.5% (abnormal); IVF/ICSI 66.67% vs 42.86%; IUI 18.18% vs 0%	Normal microecology: *Lactobacillus*-dominant, mainly *Lactobacillus crispatus* 52.34%, *Lactobacillus iners* 51.40%, *Lactobacillus jensenii* 16.82%, *Lactobacillus gasseri* 15.89%. Abnormal microecology: increased *Ureaplasma urealyticum* 73.83%, *Prevotella species* 70.09%, *Gardnerella vaginalis* 53.27%, *Mobiluncus curtisii* 25.23%, *Atopobacteria* 14.95%	N/A
Tong et al., 2023 [53]	Vaginal swabs	IVF	IVF clinical pregnancy rate significantly higher in women with more diverse vaginal microbiota	*Lactobacillus* was the dominant genus in all samples. Lower microbial diversity, particularly in the first trimester, was associated with preterm birth. Specific bacteria such as *Alloscardovia* were more prevalent in successful term deliveries, while *Vibrio* and *Sporosarcina* were linked to preterm delivery risk	α-diversity: Higher diversity in women who delivered at term; lower diversity in women who delivered preterm. β-diversity: Significant differences in bacterial community composition between term and preterm delivery groups
Miyagi et al., 2023 [54]	Vaginal and endometrial secretions	IVF/ICSI/FET	21/34 (61.8%) embryo transfers; pregnancy significantly higher in High *Lactobacillus* + Low pathological bacteria groups (Endometrium: *P* = 0.022; Vagina: *P* = 0.015)	Pregnant women: high *Lactobacillus* spp. and low *Gardnerella* spp./*Prevotella* spp.; Nonpregnant women: low *Lactobacillus* spp. and high *Gardnerella* spp./*Prevotella* spp.	β-diversity (PERMANOVA): pregnancy associated with high *Lactobacillus* and low *Gardnerella* spp. abundance (Endometrium *P* = 0.048; Vagina *P* = 0.041)
Cariati et al., 2023 [55]	Endometrial fluid	IVF	Clinical pregnancy 37.6%, No pregnancy 62.4%	*Lactobacillus* spp. significantly more frequent in women who achieved clinical pregnancy. *Enterobacteriaceae* (including *Klebsiella pneumoniae*) and *Staphylococcaceae* families were significantly more abundant in non-pregnant women. *Actinobacteria* (including *Gardnerella vaginalis*, *Corynebacterium coyleae*, *Bifidobacterium scardovii*, *Microbacterium maritypicum*) detected only in non-pregnant group	N/A
Guan et al., 2023 [66]	Cervical samples	IVF/FET	CMT1 (*Lactobacillus crispatus-*dominated) had significantly higher biochemical (*P* = 0.008) and clinical (*P* = 0.006) pregnancy rates than CMT2 (*Lactobacillus iners-*dominated) and CMT3 (diverse/other bacteria)	CMT1 dominated by *Lactobacillus crispatus*; CMT2 by *Lactobacillus iners*; CMT3 by diverse bacteria including *Gardnerella vaginalis*, *Klebsiella pneumoniae*, *Atopobium vaginae*, *Streptococcus agalactiae*	α and β diversity assessed; CMT1 had highest α diversity, PCoA separated three clusters distinctly
Kadogami et al., 2023 [65]	Endometrial tissue	IVF	Implantation rate was significantly lower in patients with *Lactobacillus iners* dominance compared to other *Lactobacillus* species (*P* = 0.01)	Dominance of *Lactobacillus* species, particularly *Lactobacillus iners*, *Lactobacillus crispatus*, *Lactobacillus gasseri*, and *Lactobacillus jensenii*. *Lactobacillus iners* dominance linked with lower implantation success rates	N/A
Koort et al., 2023 [67]	Women: vaginal swabs; men: semen	IVF/ICSI	Women with *Lactobacillus crispatusdominant* or other lactic-acid-bacteria-predominant microbiomes had higher ART clinical pregnancy rate (41.2% vs 22.2%, *P* < 0.05)	ART women had reduced *Lactobacillus* and increased *Actinobacteria*, *Gardnerella*, *Atopobium*, *Bifidobacterium*, *Clostridium*, *Enterobacteriaceae* compared with healthy controls	BV-type community only in ART women; ART women showed higher bacterial richness/diversity
Väinämö et al., 2023 [25]	Vaginal swabs	IVF	Clinical pregnancy 39.5%; Live birth 34.2%	*Lactobacillus crispatus* relative abundance higher in women achieving clinical pregnancy vs non-pregnancy; *Lactobacillus iners* and BV-associated species more common in non-pregnancy; early pregnancy samples shifted toward *Lactobacillus crispatus* dominance	N/A
Bui et al., 2023 [60]	Endometrial biopsies	IVF	38/92 (41.3%) had a live birth; 54/92 (58.7%) did not	Women with a live birth had significantly higher *Lactobacillus crispatus* relative abundance	α and β diversity not significantly different
Faisal et al. 2023 [68]	Vaginal samples	ICSI	13 pregnant, 33 non-pregnant	Significant differences in *Enterobacter* spp. and *Lactobacillus* spp. between pregnant and non-pregnant groups (*P* = 0.044 for *Enterobacter* spp., *P* = 0.045 for *Lactobacillus* spp.)	N/A
Nishio et al., 2024 [57]	Cervicovaginal mucus	IVF	Pregnant *n =* 10; Non-pregnant *n =* 13	No significant difference in *Lactobacillus* abundance between groups; at embryo transfer, non-pregnant group showed increases in *Escherichia coli*, *Prevotella*, *Pseudomonas*	α-diversity (Chao1, Faith’s PD, Shannon) was not significantly different; beta diversity (weighted/unweighted UniFrac PCoA) showed no clear group separation. UniFrac PCoA) showed no clear group separation
Wu et al., 2025 [58]	Cervical mucus	IVF	Clinical pregnancy 84/131 (64%); Non-pregnancy 47/131 (36%)	*Halomonas*, *Klebsiella*, *Atopobium*, *Ligilactobacillus* higher in non-Pregnancy group; *Lactobacillus* abundance was similar between groups	α-diversity (Shannon & Simpson) no significant difference; β-diversity (PCA & PCoA) no clear clustering
Bar et al., 2025 [11]	Vaginal swabs	IVF	Clinical pregnancy 64.3%; No pregnancy 35.7%	Increased *Lactobacillus crispatus* associated with higher pregnancy outcomes, with lower microbial diversity linked to successful pregnancies	Shannon Diversity Index significantly lower for pregnant participants compared to non-pregnant ones (*P* = 0.041)
Alley et al. 2025 [59]	Vaginal swabs	FET after IVF/ICSI	55 clinical pregnancies; 32 non-pregnancies	Pregnant group showed greater *Lactobacillus* dominance; non-pregnant group had higher abundance of Enterobacteriaceae/opportunistic bacteria	α-diversity: no differences across multiple indices (Observed richness, Chao1, ACE, Shannon, Simpson, InvSimpson, Fisher). β-diversity: NMDS (Bray–Curtis) showed no clustering by outcome

*Abbreviations*: *ART* Assisted reproductive technology, *BV* Bacterial vaginosis, *AVM* Abnormal vaginal microbiota, *LD* *Lactobacillus*-dominated, *NLD* Non-*Lactobacillus*-dominated, *LDM* *Lactobacillus*-dominated microbiota, *NLDM* Non-*Lactobacillus*-dominated microbiota, *ET* Embryo transfer, *FET* Frozen embryo transfer, *IVF* In vitro fertilization, *IVF-ET* In vitro fertilization-embryo transfer, *ICSI* Intracytoplasmic sperm injection, *IUI* Intrauterine insemination, *α-diversity* within-sample microbial diversity, *β-diversity* between-sample microbial diversity, *OTU* Operational taxonomic unit, *qPCR* quantitative polymerase chain reaction, *PCoA* Principal coordinates analysis, *NMDS* Non-metric multidimensional scaling, *PLS-DA* Partial least squares discriminant analysis, *LEfSe* linear discriminant analysis effect size, *Shannon index* Shannon diversity index, *Simpson index* Simpson diversity index, *Chao1* species richness estimator, *Faith’s PD* Faith’s phylogenetic diversity, *CMT* Cervical microbiota type, *PELORA* Penalized logistic regression analysis

In contrast, non-*Lactobacillus*-dominant microbiota or communities enriched with potentially pathogenic taxa, including *Gardnerella vaginalis*, *Atopobium vaginae*, *Streptococcus* species, *Prevotella* species, and *Bifidobacterium* species, are generally reported to be associated with poorer ART outcomes, including lower implantation and clinical pregnancy rates, increased miscarriage, and reduced live birth rates. Abnormal vaginal microbiota, defined by elevated *Gardnerella vaginalis* and/or *Atopobium vaginae*, was associated with lower clinical pregnancy rates [40, 44]. In women with an unfavorable vaginal microbiome profile, characterized by low overall *Lactobacillus* (less than 20%), high *Lactobacillus jensenii* (greater than 35%), presence of *Gardnerella vaginalis* IS-pro type 1, or high *Proteobacteria* (greater than 28%), pregnancy rates were only 6% compared with approximately 35% in the overall cohort [10]. Severe vaginal dysbiosis reduced implantation rates from 66.9% in *Lactobacillus*-dominant women to 29.6% and clinical pregnancy rates from 84.3% to 34.2% [50]. Similarly, higher *Lactobacillus* abundance and lower levels of *Gardnerella*, *Atopobium*, and *Prevotella* were associated with a clinical pregnancy rate of 50.1% [61]. Non-*Lactobacillus*-dominant vaginal microbiota was also associated with lower live birth rates, 31.8% versus 41.2% in *Lactobacillus*-dominant women [62]. Studies of cervical and endometrial microbiota reported that enrichment of pathogenic taxa was associated with poorer ART outcomes [51, 52, 54, 55].

Some non-*Lactobacillus*-dominant communities, such as *Bifidobacterium*-predominant microbiota, were reported in association with successful implantation in certain cases, although implantation and pregnancy rates were generally lower than in *Lactobacillus*-dominant communities [20, 43]. Among *Lactobacillus* species, *Lactobacillus iners* appeared less consistently associated with favorable outcomes than *Lactobacillus crispatus* [65, 67].

Several studies reported that microbial diversity or overall *Lactobacillus* abundance alone did not consistently distinguish pregnancy outcomes [3, 20, 32, 42, 47, 57, 58]. *Lactobacillus*-dominant communities were generally more stable in early pregnancy, whereas non-dominant communities appeared more variable over time, with shifts in early pregnancy often favoring *Lactobacillus crispatus* dominance [25].

#### Microbial diversity and ART outcome

Analyses of microbial diversity yielded inconsistent findings across studies (Table 3 and Fig. 2). Alpha diversity (within-sample diversity) was higher in non-pregnant patients in some studies [24, 61], higher in pregnant women in others [69], and frequently showed no significant differences [47, 57, 58]. One study reported significantly lower alpha diversity in pregnant women compared with non-pregnant participants [11]. Beta diversity (between-sample diversity) occasionally demonstrated clustering differences between pregnant and non-pregnant groups [54, 69], whereas most studies reported no clear separation [58, 59]. Overall, current evidence does not support a consistent relationship between microbial diversity metrics alone and ART outcomes.

#### Sample site and pregnancy outcomes

Sample site appeared to influence microbiota associations with ART outcomes (Table 3 and Fig. 2). In vaginal samples, *Lactobacillus* dominance, particularly *Lactobacillus crispatus*, was generally associated with higher implantation and clinical pregnancy rates, whereas enrichment of *Gardnerella*, *Atopobium*, *Prevotella*, or *Enterobacter* spp. was more frequently observed in women with lower pregnancy rates [25, 49, 50, 64, 68].

In cervical samples, *Lactobacillus crispatus* dominance (CMT1) was associated with higher pregnancy rates, while *Lactobacillus iners* dominance or diverse bacterial communities (CMT2/CMT3) were more frequently associated with lower pregnancy rates; *Atopobium vaginae* was higher in unfavorable profiles [51, 66].

In endometrial samples, *Lactobacillus* dominance was associated with successful implantation and live birth, whereas the presence of pathogenic bacteria including *Gardnerella*, *Atopobium*, *Streptococcus*, and *Klebsiella* was more frequently detected in cases of implantation failure, no pregnancy, or miscarriage [41, 46, 52, 55, 65].

### Follicular fluid microbiota

Three studies investigated associations between follicular fluid microbiota and IVF/ICSI outcomes (Table 4 and Fig. 2) [32, 34, 48]. Pelzer et al. reported that the presence of *Lactobacillus* spp. in follicular fluid was associated with higher embryo transfer rates and improved pregnancy outcomes, whereas colonization with *Propionibacterium*, *Streptococcus*, *Actinomyces*, and *Bifidobacterium* spp. was associated with lower embryo transfer success or negative pregnancy outcomes [34]. Usman et al. detected microorganisms in 17% of follicular fluid samples, including *Staphylococcus aureus*, *Streptococcus* spp., *Enterococcus* spp., *Lactobacillus* spp., and *Candida albicans* [48]. After adjustment for clinical and procedural covariates, no statistically significant associations were identified between microbial detection and fertilization, implantation, or pregnancy outcomes.

**Table 4:** Summary of follicular fluid microbiota findings in IVF/ICSI pregnancy outcomes

Author (Year)	Sample type	ART	Pregnancy outcomes	Microbiota change related to pregnancy outcomes	Microbial diversity analysis	Key findings
Pelzer et al., 2013 [34]	Follicular fluid	IVF	Fertilization, embryo transfer, and pregnancy rates were generally not affected by microbial colonization; however, embryo transfer rates tended to be higher in follicles with *Lactobacillus* spp.	Presence of *Lactobacillus* spp. linked to higher embryo transfer rates; *Propionibacterium*, *Streptococcus*, *Actinomyces*, and *Bifidobacterium* spp. associated with lower embryo transfer or negative pregnancy outcomes	N/A	Follicular fluid contains both colonizing and potential contaminating microorganisms; *Lactobacillus* spp. correlated with improved embryo transfer and pregnancy rates, while certain other bacteria were linked to poorer outcomes
Usman et al., 2021 [48]	Follicular fluid	IVF	Fertilization: 81% (follicular fluid colonized) vs. 64% (non-colonized); Biochemical pregnancy: 33% vs. 21%; Clinical pregnancy: 27% vs. 21%. After adjustment, no significant association with fertilization or pregnancy outcomes was observed	Follicular fluid microorganisms detected in 17% (15/86); 17% contaminants; colonizers included *Staphylococcus aureus* (7/15), *Streptococcus* spp. (4/15), *Enterococcus* spp. (4/15), *Lactobacillus* spp. (2/15), *Candida albicans* (2/15)	N/A	Isolation of microorganisms was not associated with fertilization or pregnancy outcomes
Kim et al. 2022 [32]	Follicular fluid	IVF/ICSI	Clinical outcomes, including fertilization, implantation, and pregnancy rates, tended to be higher in the negative follicular fluid culture group than in the positive group, without statistical significance	Follicular fluid microorganisms were not associated with embryo quality or clinical pregnancy rates during IVF cycles	N/A	No clear impact of follicular fluid microbiota on embryo quality or pregnancy outcomes

*Abbreviations*: *IVF* In vitro fertilization, *ICSI* Intracytoplasmic sperm injection, *IVF-ET* In vitro fertilization-embryo transfer, *N/A* Not assessed

Similarly, Kim et al. reported that although clinical outcomes appeared numerically higher in cycles with culture-negative follicular fluid compared with culture-positive samples, these differences were not statistically significant, and no consistent associations with embryo quality or clinical pregnancy rates were observed [32].

Across all included studies, follicular fluid analyses were based primarily on culture-dependent methods or targeted detection approaches. None of the studies performed formal α- or β-diversity analyses, and sequencing-based characterization of follicular fluid microbiota was limited, restricting evaluation of overall community structure in relation to IVF/ICSI outcomes.

### Probiotic interventions

Five studies, including RCTs and observational cohorts, reported associations between probiotic supplementation and ART outcomes in women undergoing IVF/ICSI (Table 5) [38, 39, 45, 56, 63]. *Lactobacillus*-containing probiotics were administered orally or vaginally, with intervention durations ranging from a single post-oocyte retrieval dose to six months prior to IVF.

**Table 5:** Summary of studies on probiotic interventions in women undergoing IVF/ICSI and pregnancy outcomes

Study (year)	Study design	ART	Probiotic intervention (strain; formulation)	Dose/route	Timing and duration	Main microbiota effects	Microbial diversity analysis	Pregnancy outcomes	Key finding
Gilboa et al., 2005 [38]	RCT (*N =* 117; 50 probiotic, 67 control)	IVF	*Lactobacillus acidophilus*, *Bifidobacterium bifidum*, *Bifidobacterium longum* (intravaginal capsules)	2 capsules, total 6 × 10⁹ CFU; vaginal	Single administration immediately post-oocyte retrieval	No significant changes in vaginal microbiota; *Lactobacilli* presence not enhanced	N/A	No significant between-group differences were observed in fertilization rate, number of embryos transferred, or pregnancy rate	Intravaginal probiotic supplementation immediately after oocyte retrieval had no effect on vaginal colonization or pregnancy rate
Iwami et al., 2020 [45]	Prospective multicenter cohort (*N =* 527)	IVF	Probiotics not fully specified; EMMA-guided (± antimicrobials)	Oral or vaginal (center-specific)	From EMMA result to subsequent ET (duration not specified)	Increased *Lactobacillus*-dominated endometrial microbiota; reduced *Gardnerella* and other pathogens	N/A	Ongoing pregnancy rates after intervention comparable to Normal group; earlier achievement of pregnancy in Abnormal group (*P* = 0.031)	EMMA-guided probiotic intervention corrected endometrial dysbiosis and improved time to pregnancy in patients with recurrent implantation failure or pregnancy loss
Di Pierro et al. 2023 [63]	Retrospective, controlled, observational (*N =* 160; 80 probiotic, 80 control)	IVF/ICSI	*Lactobacillus crispatus* M247 (oral sachet)	≥ 2 × 10^1^⁰ CFU/day; oral	90 days before ART	Intended restoration of vaginal eubiosis (post-negative swab or post-antibiotics)	N/A	Higher clinical pregnancy (OR 1.56) and live birth (OR 1.76); strongest effect observed in blastocyst subgroup	Oral *Lactobacillus crispatus* M247 was well-tolerated and associated with higher clinical pregnancy and live-birth rates, especially in the blastocyst subgroup
Iwami et al. 2023 [56]	Prospective cohort (*N =* 122; 61 study, 61 control)	IVF/FET	*Lactobacillus* spp.* (vaginal suppositories)*	Vaginal	7–17 days (EMMA-guided)	Restoration of *Lactobacillus*-dominated endometrium; reduced non-*Lactobacillus* pathogens	N/A	Cumulative clinical pregnancy: 64.5% vs 33.3%; ongoing pregnancy: 48.9% vs 32.8%	Personalized 16S-guided vaginal probiotic therapy improved clinical and ongoing pregnancy rates in women with RIF
Huerga López et al., 2025 [39]	RCT (*N =* 70 couples; 57 completed)	IVF	*Ligilactobacillus salivarius* CECT5713 (oral capsules; both partners)	Oral; dose per manufacturer	6 months pre-IVF; continued during cycle and 12 weeks if pregnant	Vaginal 16S showed minimal compositional change; qPCR confirmed increased *Ligilactobacillus salivarius*; improved semen immune	α/β diversity unchanged	Total pregnancy: 51.8% vs 26.7%; live birth: 48% vs 20% (*P* = 0.024)	Oral *Ligilactobacillus salivarius* CECT5713 increased live-birth rates without major vaginal compositional shifts, suggesting. immunomodulatory and male-factor

*Abbreviations*: *ART* Assisted reproductive technology, *RCT* Randomized controlled trial, *IVF* In vitro fertilization, *ICSI* Intracytoplasmic sperm injection, *FET* Frozen embryo transfer, *ET* Embryo transfer, *EMMA* Endometrial Microbiome Metagenomic Analysis, *RIF* Recurrent implantation failure, *OR* Odds ratio, *N/A* Not assessed, *CFU* Colony-forming units, *qPCR* quantitative polymerase chain reaction

Gilboa et al. administered intravaginal *Lactobacillus acidophilus* and *Bifidobacterium bifidum/longum* immediately after oocyte retrieval [38]. This single-dose intervention did not significantly alter vaginal microbiota or affect fertilization, embryo transfer, or pregnancy rate.

Iwami et al. implemented personalized oral or vaginal *Lactobacillus*-based interventions guided by EMMA endometrial microbiota analysis [45, 56]. These interventions were associated with increased *Lactobacillus* abundance, reduced pathogenic bacteria including *Gardnerella*, and faster achievement of ongoing pregnancies in women with RIF or pregnancy loss.

Di Pierro et al. administered oral *Lactobacillus crispatus* M247 daily for 90 days prior to ART [63]. Higher clinical pregnancy and live-birth rates were observed, particularly in women aged 30–40 years and in the blastocyst transfer subgroup.

Huerga López et al. evaluated oral *Ligilactobacillus salivarius* CECT5713 to both partners for six months before IVF, continuing for 12 weeks if pregnancy occurred [39]. Despite minimal changes in vaginal microbial diversity, the probiotic group demonstrated higher total pregnancy and live-birth rates compared with placebo.

Microbial diversity data were limited. Gilboa et al. [38] and Di Pierro et al. [63] did not report diversity metrics. Iwami et al. [45, 56] reported shifts toward *Lactobacillus*-dominated endometrium without α- or β-diversity analyses. Huerga López et al. [39] found no significant changes in vaginal α- or β-diversity despite increased *Ligilactobacillus salivarius* and improved pregnancy outcomes. These findings suggest probiotics may alter microbial composition without affecting overall diversity, but causality cannot be inferred.

## Discussion

### Reproductive tract microbiota in IVF/ICSI pregnancy outcomes

Accumulating evidence suggests that reproductive tract microbiota is associated with ART outcomes. A *Lactobacillus*-dominant microbiota, particularly *Lactobacillus crispatus*, has been frequently reported to be associated with higher implantation, clinical pregnancy, and live birth rates [11, 24, 25, 41, 59, 66]. In contrast, non-*Lactobacillus*-dominant or pathogen-enriched communities, including *Gardnerella*, *Atopobium*, and *Prevotella*, are associated with reduced implantation and higher miscarriage rates [10, 40, 44, 50–52, 54, 55, 61, 62]. However, these associations are limited by incomplete and inconsistent adjustment for potential confounders across studies, including hormonal status, BMI, antibiotic exposure, infertility diagnosis, and ovarian stimulation protocols.

At the species level, *Lactobacillus iners* is generally associated with less favorable outcomes compared with *Lactobacillus crispatus*, whereas *Bifidobacterium*-predominant profiles may show limited supportive effects but remain inconsistent [20, 43, 60, 65, 67]. This indicates that reproductive outcomes are influenced more by species-level composition than by overall dominance alone.

Microbial diversity metrics are inconsistent. Alpha diversity has been reported as higher in non-pregnant women [24, 61], higher in pregnant women [69], or not significantly different across studies [11, 47, 57, 58]. Notably, Hao et al. [69] reported significantly higher cervical mucus alpha diversity in women who achieved pregnancy following fresh IVF-ET, contrasting with the broader trend linking lower diversity and *Lactobacillus* dominance to favorable reproductive outcomes. This finding suggests that associations between microbial diversity and ART outcomes may vary according to study context and sampling site. Similarly, beta diversity occasionally reveals clustering between pregnant and non-pregnant groups [54, 69], although most studies do not observe clear separation [58, 59]. Overall, species-level composition appears more informative than diversity indices for distinguishing reproductive outcomes.

Temporal and anatomical variability further complicates interpretation. *Lactobacillus*-dominant communities appear more stable during early pregnancy, whereas non-dominant microbiota may shift over time [25]. In addition, vaginal, cervical, and endometrial microbiota differ in composition, with endometrial dysbiosis more frequently linked to adverse outcomes [32, 41, 46, 52, 55, 65, 66].

Collectively, current evidence indicates an association between reproductive tract microbiota and ART outcomes, although heterogeneity in study design, confounding adjustment, and sampling methods precludes causal inference. Further well-designed longitudinal studies with standardized methodologies are needed to clarify these relationships.

### Vaginal, cervical, and endometrial microbiota: predictive value and limitations

Vaginal microbiota, particularly *Lactobacillus* dominance, has been widely studied as a non-invasive factor associated with ART outcomes. Higher *Lactobacillus* abundance is generally associated with higher implantation, clinical pregnancy, and live birth rates [11, 24, 25, 41, 59, 66]. However, it remains an imperfect surrogate for endometrial microbiota. Approximately 20% of women show discordance between vaginal and endometrial profiles [41], where *Lactobacillus*-dominant vaginal microbiota may coexist with a non-*Lactobacillus*-dominant endometrial profile, and vice versa. These findings indicate that vaginal microbiota should be interpreted cautiously and not assumed to directly reflect endometrial status.

Cervical microbiota may provide an intermediate representation between vaginal and endometrial environments. Studies show that cervical *Lactobacillus* dominance, particularly by *Lactobacillus crispatus*, is associated with higher biochemical and clinical pregnancy rates, whereas *Lactobacillus iners*-dominant or more diverse communities are more often observed in implantation failure [52, 66]. These differences may relate to local microbial niche specificity, hormonal regulation, and immune modulation at the reproductive tract interface.

Overall, vaginal profiling may help identify overt dysbiosis, while cervical microbiota may provide additional insight into implantation-associated microbial patterns. Endometrial microbiota assessment remains the most direct indicator of the uterine environment, although its invasive nature limits routine application. A multi-site assessment strategy may therefore offer a more comprehensive characterization of the reproductive tract microbiome in ART.

### Follicular fluid microbiota in IVF/ICSI pregnancy outcomes

Follicular fluid is not entirely sterile, but its association with IVF/ICSI outcomes remains uncertain, and current evidence is limited. As a low-biomass sample, follicular fluid microbiota studies are highly susceptible to contamination from environmental and reagent-derived DNA, which may confound interpretation of reported microbial signals. Some studies reported that *Lactobacillus* spp. were more frequently detected in cycles with embryo transfer or pregnancy, whereas *Propionibacterium*, *Streptococcus*, and *Actinomyces* were more often observed in unsuccessful cycles [32, 34, 48]. However, these associations are inconsistent and may be influenced by methodological variability, low microbial biomass, and potential contamination.

Low-abundance bacterial signals have been detected in follicular fluid, but most microbial DNA is likely derived from contamination or transient introduction during oocyte retrieval rather than true colonization [34, 70, 71]. Reported taxa often overlap with vaginal, gut, skin, or oral microbiota, including *Lactobacillus*, *Bifidobacterium*, *Staphylococcus*, and *Streptococcus* spp. [33, 34]. Methodologically, limited use of high-throughput sequencing, with reliance on culture-based approaches, restricts comprehensive characterization of follicular fluid microbiota.

Overall, follicular fluid microbiota shows no consistent association with embryo quality or pregnancy outcomes, whereas vaginal dysbiosis is more reproducibly linked to reduced implantation and live birth rates [32]. Current evidence therefore does not support a clinically actionable role for follicular fluid microbiota in IVF/ICSI outcomes, and microbiota-targeted interventions in this compartment remain premature.

### Probiotic interventions

A *Lactobacillus*-dominant reproductive tract microbiota has been associated with uterine receptivity and successful implantation, whereas dysbiosis may impair fertility and ART outcomes [10, 40, 72, 73]. Accordingly, probiotic supplementation has been investigated to enhance IVF/ICSI success, though effects vary by bacterial strain, administration route, duration, and study design [38, 39, 45, 56, 63]. Causality cannot be inferred given the predominantly observational and small-scale nature of available studies, and observed effects may also be influenced by host immune or metabolic factors rather than direct microbial modulation.

Short-term intravaginal administration immediately after oocyte retrieval appears to have limited impact on microbiota composition and has not demonstrated clear improvement in pregnancy outcomes [38]. In contrast, some studies evaluating longer-duration or individualized interventions, including EMMA-guided oral or vaginal *Lactobacillus* [45, 56], 90 days of oral *Lactobacillus crispatus* M247 [63], and six months of oral *Ligilactobacillus salivarius* CECT5713 [39], have reported increased *Lactobacillus* abundance and improved reproductive outcomes in selected cohorts; however, evidence remains limited by the small number of randomized trials and conflicting results.

Some studies reported higher pregnancy and live birth rates despite minimal microbiota changes, suggesting that probiotics may also act through immunomodulatory pathways, although evidence is preliminary [39]. Sequential antibiotic-probiotic regimens have been proposed in selected cases, in which antibiotics suppress potential pathogens and probiotics restore microbial balance [43]. However, optimal timing and protocols remain uncertain, and unnecessary antibiotic use may exacerbate dysbiosis.

Emerging evidence indicates potential benefits for male partners. Probiotic supplementation has been reported to increase GM-CSF levels in seminal plasma [39], a cytokine linked to sperm quality in vitro [74–77] and to improved embryo development in animal models [78]. Effects on the male reproductive tract microbiota remain largely unexplored [67, 79, 80].

No studies have directly evaluated probiotic effects on follicular fluid microbiota, leaving its potential impact on oocyte quality and ART outcomes uncertain. Overall, heterogeneity in study design and limited high-quality RCTs highlight the need for further well-designed studies to clarify efficacy and mechanisms.

### Mechanistic insights into microbiota-IVF/ICSI interactions

Reproductive tract microbiota has been proposed to influence fertilization, implantation, and pregnancy outcomes in IVF/ICSI through metabolic, epithelial, and immune-mediated pathways. *Lactobacillus* species, particularly *Lactobacillus crispatus* and *Lactobacillus jensenii*, ferment glycogen to lactic acid, contributing to a low pH environment that suppresses anaerobic pathogens such as *Gardnerella* and *Prevotella*. Lactic acid and hydrogen peroxide may further exert antimicrobial and immunomodulatory effects that support implantation-favorable conditions [81, 82].

These bacteria are thought to contribute to epithelial barrier stability by competing for adhesion sites, secreting bacteriocins, limiting pathogenic biofilm formation, and reducing inflammatory responses [83, 84]. In contrast, pathogens such as *Gardnerella vaginalis* produce sialidases capable of degrading epithelial glycans and compromising mucosal integrity [85]. Microbiota have also been implicated in modulation of local immune responses through pattern recognition receptor signaling, influencing the balance between pro-inflammatory cytokines (IL-6, IL-8, TNF-α) and anti-inflammatory mediators such as IL-10. *Lactobacillus*-derived metabolites have been proposed to support immune tolerance, whereas dysbiotic communities have been associated with increased inflammatory responses that may adversely affect implantation [86, 87].

**Fig. 3:**
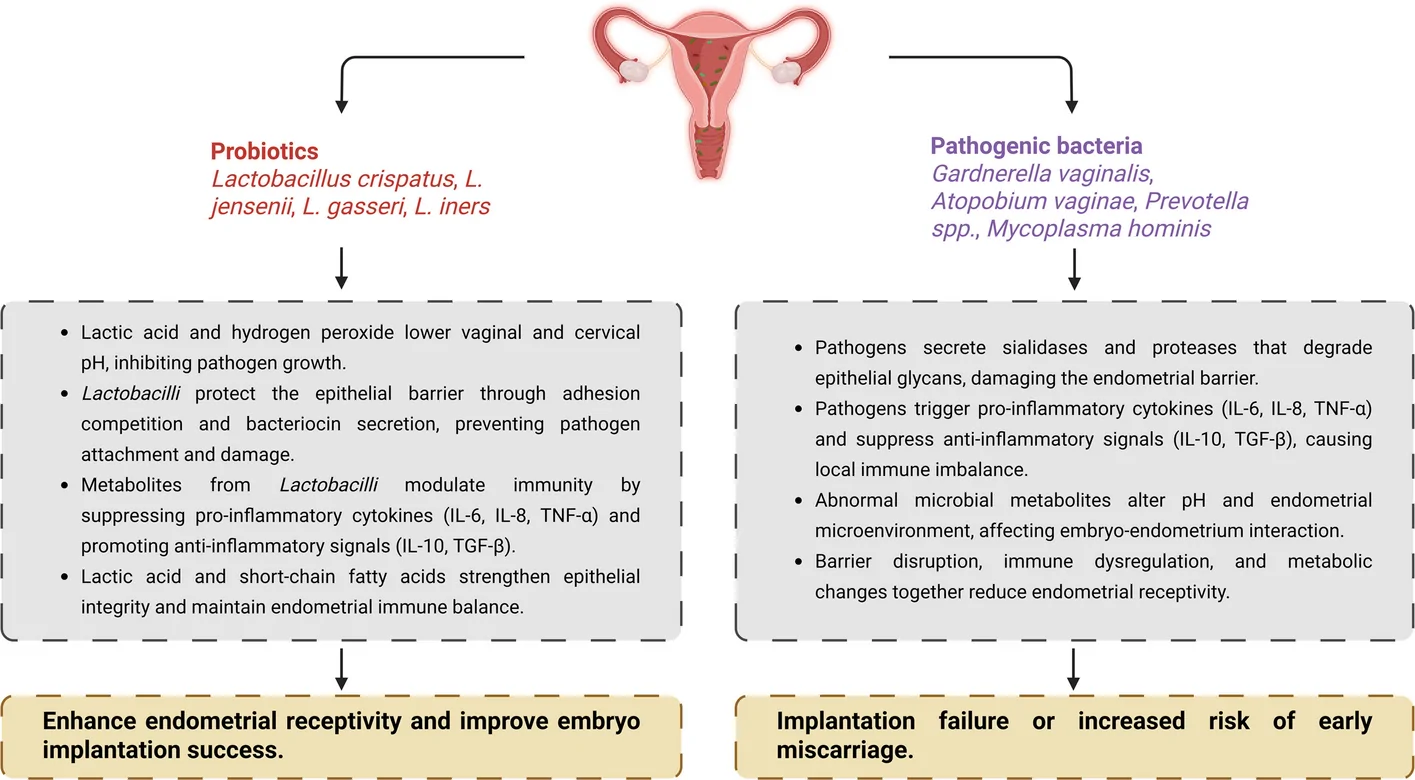
Hypothesized mechanisms linking vaginal and endometrial microbiota to implantation outcomes. This figure was created with BioRender.com

Probiotic interventions have been explored to restore *Lactobacillus* dominance and modulate inflammatory microbial communities; however, evidence for consistent improvements in pregnancy outcomes remains limited and heterogeneous [39, 88–90]. Emerging data further suggest functional interactions or signaling across vaginal, cervical, and uterine compartments, indicating a coordinated reproductive microbial ecosystem [90, 91]. Figure 3 summarizes the hypothesized interactions between reproductive tract microbiota and implantation.

### Comparison with previous literature

Previous systematic reviews have advanced our understanding of reproductive tract microbiota and ART outcomes, but important gaps remain. Most focused on the vaginal, cervical, and endometrial microbiota and their associations with fertility or ART success [92], largely overlooking follicular fluid microbiota, which may affect oocyte quality and fertilization potential, and relying primarily on observational data without systematically evaluating interventional approaches such as probiotics. Subsequent meta-analyses quantified associations between vaginal microbiota and IVF/ICSI outcomes [93], but their scope was limited to the vaginal compartment and the effects of dysbiosis, without addressing other reproductive sites or interventional evidence.

In contrast, this systematic review provides a more comprehensive and up-to-date synthesis by integrating evidence on reproductive tract and follicular fluid microbiota, as well as emerging studies on probiotic interventions in IVF/ICSI outcomes. Covering literature up to August 2025, it offers a broader overview linking microbiota composition, interventional strategies, and ART outcomes.

### New insights and future perspectives: semen microbiota and ART outcomes

Research on reproductive microbiota has traditionally focused on the maternal tract; however, emerging evidence suggests a role for semen microbiota in ART outcomes. This systematic review identified one study showing that semen microbiota composition is associated with ART success [67]. Men with genital tracts enriched in Gram-negative anaerobic or microaerophilic bacteria, including *Prevotella* and related taxa, had lower implantation and pregnancy rates, whereas more favorable microbial profiles were associated with better outcomes [67]. These findings suggest that male reproductive tract dysbiosis may impair sperm function and early embryo development, reducing ART efficacy.

Consistent evidence indicates that *Lactobacillus* and *Prevotella* exert opposing effects on sperm quality and DNA integrity [94, 95]. Reduced *Lactobacillus* is associated with impaired motility and altered seminal characteristics, whereas increased *Prevotella* correlates with poorer motility and reduced semen quality [79, 95–99]. Given the central role of sperm quality in fertilization and embryo development, semen microbiota may indirectly influence ART outcomes.

Future research should investigate mechanisms by which *Lactobacillus* and *Prevotella* affect sperm function, DNA integrity, and embryo development via microbial metabolites, oxidative stress, and immune pathways. Interventional studies are needed to evaluate probiotics and lifestyle or dietary strategies on semen microbiota and ART outcomes. Semen microbiota may also serve as a biomarker for male fertility. Longitudinal multi-omics and experimental models are required to establish causality and support clinical translation.

### Low-biomass sample contamination and methodological considerations

Low-biomass reproductive tract samples, including endometrial tissue or fluid, cervical mucus, and follicular fluid, are highly susceptible to contamination, which may generate artefactual microbial signals and bias associations with IVF/ICSI outcomes. We therefore systematically assessed contamination control measures across studies, including negative (no-template) controls, extraction blanks, sterile sampling procedures, and computational decontamination pipelines.

Only five studies implemented comprehensive contamination control across all four domains. Importantly, these methodologically robust studies were consistent with the overall synthesis, suggesting an association between *Lactobacillus*-dominant endometrial microbiota and improved reproductive outcomes, whereas dysbiotic profiles were more frequently linked to adverse outcomes. In contrast, studies with incomplete contamination control showed greater heterogeneity and should be interpreted with caution.

These findings indicate that, although contamination is a key methodological limitation in low-biomass microbiome research, the observed associations are unlikely to be entirely driven by artefactual signals. However, the small number of rigorously controlled studies underscores the need for standardized, contamination-aware study designs in future research.

### Limitations of the systematic review

This systematic review has several limitations. Methodological heterogeneity is a major issue in microbiome research, including differences in sample collection, contamination control, sequencing platforms, diversity analysis, sampling sites, timing, and outcome definitions [100–102]. Microbiota composition and biological relevance may also differ across vaginal, cervical, endometrial, and follicular compartments, further complicating direct comparison between studies. In addition, key confounders such as hormonal status, BMI, infertility etiology, and ovarian stimulation protocols were not consistently controlled, limiting comparability across studies. Reproductive tract samples are often low-biomass and vulnerable to contamination, which further affects data reliability and may contribute to inconsistent findings, particularly for follicular fluid microbiota.

Most included studies were observational with moderate methodological quality, and only a limited number of RCTs were available, which limits the ability to support causal inferences. Probiotic intervention studies were also heterogeneous in terms of microbial strains, administration routes, and treatment durations. Some studies reported improved ART outcomes without corresponding changes in microbial composition, suggesting that observed associations may also be influenced by indirect pathways, including host-related or immune-mediated factors; however, these mechanisms remain speculative and require further investigation.

Additional limitations include potential sponsorship bias in some probiotic studies, particularly in study design, outcome reporting, or selective publication. Restricting inclusion to English-language publications may also have introduced selection bias. Overall, these limitations highlight the need for standardized, site-specific sampling, harmonized outcome definitions, and more rigorous study designs. In particular, adequately powered RCTs with standardized microbiota assessment are required to validate these associations and further clarify their biological basis. Current evidence should therefore be interpreted with appropriate caution.

### Clinical implications and future directions

These findings suggest a potential role for reproductive tract microbiota profiling in supporting more personalized IVF/ICSI strategies; however, current evidence remains insufficient to justify routine microbiota-targeted interventions in clinical practice. Clinical application of microbiota-based interventions should therefore remain restricted to well-designed research settings.

Mechanistic research is needed to clarify species- and strain-specific effects on implantation, early pregnancy, and host immune-metabolic interactions. Longitudinal studies with standardized sampling and microbiota assessment are required to characterize microbial dynamics across ART cycles and improve comparability across studies. Species- or strain-level resolution, particularly for *Lactobacillus* spp. and relevant pathogens, together with live birth as the primary endpoint, should be prioritized.

Probiotic interventions should clearly specify strain, dose, route, timing, and duration, ideally guided by individualized microbial profiling. Complementary mechanistic substudies integrating immune-metabolic profiling, multi-omics approaches, and semen microbiota analysis may help elucidate causal pathways.

We propose a pragmatic RCT randomizing women with non-dominant or dysbiotic microbiota to oral or vaginal *Lactobacillus*-based probiotics versus placebo or standard care, with sampling at baseline, pre-transfer, and early pregnancy, and follow-up until delivery or early loss. Pre-registration, transparent funding disclosure, and conflict-of-interest reporting are essential to enhance reproducibility and minimize bias.

## Conclusion

*Lactobacillus*-dominant reproductive tract microbiota, particularly *Lactobacillus crispatus*, is frequently reported to be associated with improved IVF/ICSI outcomes, whereas pathogen-enriched or non-*Lactobacillus* communities are associated with poorer reproductive outcomes. Species-level composition and site-specific microbiota profiles appear to provide more informative signals than overall microbial diversity; however, the evidence remains inconsistent and poorly reproducible, and causal inference cannot be established. Some studies suggest that probiotic interventions may increase *Lactobacillus* abundance, but these findings remain preliminary and are supported by limited and discordant randomized controlled evidence. Methodological heterogeneity, including variability in contamination-control practices and the exploratory nature of early pilot studies, further limits the robustness of current evidence. Overall, current evidence is not yet sufficient to support routine clinical application of microbiota assessment or microbiota-targeted interventions in assisted reproductive technology practice.

## Supplementary Information

Supplementary Material 1.

Supplementary Material 2.

Supplementary Material 3.

Supplementary Material 4.

## Data Availability

Data sharing is not applicable to this article as no datasets were generated or analyzed during the current study.
