# Supplementary material for: Reproductive tract and follicular fluid microbiota and probiotic interventions in IVF/ICSI outcomes: a systematic review
Source: BMC Pregnancy Childbirth. 2026 Jul 4;26:1054. doi: 10.1186/s12884-026-09523-1 (PMC13613614; doi:10.1186/s12884-026-09523-1)
Supplement: Supplementary file 1 [file 12884_2026_9523_MOESM1_ESM.docx]

**Supplementary Table 1. Detailed search strategy for the systematic review**

**1. PubMed search strategy (Stepwise format)**

**#1 Reproductive tract & follicular fluid terms**

("Reproductive Tract"[MeSH Terms] OR reproductive tract[tiab] OR "Vagina"[MeSH Terms] OR vagina[tiab] OR "Cervix"[MeSH Terms] OR cervix[tiab] OR cervical[tiab] OR "Uterus"[MeSH Terms] OR uterus[tiab] OR "Endometrium"[MeSH Terms] OR endometrium[tiab] OR endometrial[tiab] OR "Follicular Fluid"[MeSH Terms] OR "follicular fluid"[ tiab] OR "ovarian follicle"[tiab] OR follicle[tiab] OR oocyte[tiab])

**#2 Microbiota & probiotics**

("Microbiota"[MeSH Terms] OR microbiota[tiab] OR microbiome[tiab] OR "Microbial Flora"[tiab] OR flora[tiab] OR bacteri*[tiab] OR "Bacteria"[MeSH Terms] OR dysbiosis[tiab] OR "microbial composition"[tiab] OR "Probiotics"[MeSH Terms] OR probiotic[tiab] OR Lactobacillus[tiab] OR Bifidobacterium[tiab])

**#3** **ART terms**

("In Vitro Fertilization"[MeSH Terms] OR "in vitro fertilization"[tiab] OR IVF[tiab] OR

"Intracytoplasmic Sperm Injection"[MeSH Terms] OR "intracytoplasmic sperm injection"[tiab] OR ICSI[tiab] OR "Assisted Reproductive Techniques"[MeSH Terms] OR "assisted reproductive technology"[tiab] OR ART[tiab] OR "assisted reproduction"[tiab] OR "assisted reproductive techniques"[tiab])

**#4 Pregnancy outcome terms**

("Pregnancy Outcome"[MeSH Terms] OR pregnancy outcome[tiab] OR "pregnancy"[MeSH Terms] OR pregnancy[tiab] OR "pregnancy rate"[tiab] OR implantation[tiab] OR "implantation failure"[tiab] OR "embryo implantation"[tiab] OR "live birth"[tiab] OR livebirth[tiab] OR miscarriage[tiab] OR "early pregnancy loss"[tiab] OR outcome[tiab])

**#5** **Combine four dimensions**

#1 AND #2 AND #3 AND #4

**#6 Apply time range and language limits**

AND ("2000/01/01"[Date - Publication] : "2025/08/31"[Date - Publication]) AND (english[lang])

**#7 Exclude non-original articles**

NOT (Review[pt] OR "systematic review"[tiab] OR Meta-Analysis[pt] OR Case Reports[pt] OR Editorial[pt] OR Letter[pt] OR Comment[pt] OR Conference Abstract[pt])

**#8 Final combined PubMed search formula**

(#1 AND #2 AND #3 AND #4 AND ("2000/01/01"[Date - Publication] : "2025/08/31"[Date - Publication]) AND (english[lang])) NOT (Review[pt] OR "systematic review"[tiab] OR Meta-Analysis[pt] OR Case Reports[pt] OR Editorial[pt] OR Letter[pt] OR Comment[pt] OR Conference Abstract[pt])

**PubMed final search formula**

(("Reproductive Tract"[MeSH Terms] OR reproductive tract[tiab] OR "Vagina"[MeSH Terms] OR vagina[tiab] OR "Cervix"[MeSH Terms] OR cervix[tiab] OR cervical[tiab] OR "Uterus"[MeSH Terms] OR uterus[tiab] OR "Endometrium"[MeSH Terms] OR endometrium[tiab] OR endometrial[tiab] OR "Follicular Fluid"[MeSH Terms] OR "follicular fluid"[tiab] OR "ovarian follicle"[tiab] OR follicle[tiab] OR oocyte[tiab]) AND ("Microbiota"[MeSH Terms] OR microbiota[tiab] OR microbiome[tiab] OR "Microbial Flora"[tiab] OR flora[tiab] OR bacteri*[tiab] OR "Bacteria"[MeSH Terms] OR dysbiosis[tiab] OR "microbial composition"[tiab] OR "Probiotics"[MeSH Terms] OR probiotic[tiab] OR Lactobacillus[tiab] OR Bifidobacterium[tiab]) AND ("In Vitro Fertilization"[MeSH Terms] OR "in vitro fertilization"[tiab] OR IVF[tiab] OR "Intracytoplasmic Sperm Injection"[MeSH Terms] OR "intracytoplasmic sperm injection"[tiab] OR ICSI[tiab] OR "Assisted Reproductive Techniques"[MeSH Terms] OR "assisted reproductive technology"[tiab] OR ART[tiab] OR "assisted reproduction"[tiab] OR "assisted reproductive techniques"[tiab]) AND ("Pregnancy Outcome"[MeSH Terms] OR pregnancy outcome[tiab] OR "pregnancy"[MeSH Terms] OR pregnancy[tiab] OR "pregnancy rate"[tiab] OR implantation[tiab] OR "implantation failure"[tiab] OR "embryo implantation"[tiab] OR "live birth"[tiab] OR livebirth[tiab] OR miscarriage[tiab] OR "early pregnancy loss"[tiab] OR outcome[tiab]) AND ("2000/01/01"[Date - Publication] : "2025/08/31"[Date - Publication]) AND (english[lang])) NOT (Review[pt] OR "systematic review"[tiab] OR Meta-Analysis[pt] OR Case Reports[pt] OR Editorial[pt] OR Letter[pt] OR Comment[pt] OR Conference Abstract[pt])

**2. Ovid--Embase search strategy (Stepwise format)**

**#1 Reproductive tract & follicular fluid terms**

('reproductive tract'/exp OR 'reproductive tract':ti,ab OR 'vagina'/exp OR vagina:ti,ab OR 'cervix'/exp OR cervix:ti,ab OR cervical:ti,ab OR 'uterus'/exp OR uterus:ti,ab OR 'endometrium'/exp OR endometrium:ti,ab OR endometrial:ti,ab OR 'follicular fluid':ti,ab OR 'ovarian follicle':ti,ab OR follicle:ti,ab OR oocyte:ti,ab)

**#2 Microbiota & probiotics**

('microbiota'/exp OR microbiota:ti,ab OR microbiome:ti,ab OR 'microbial flora':ti,ab OR flora:ti,ab OR bacteri*:ti,ab OR 'bacteria'/exp OR dysbiosis:ti,ab OR 'microbial composition':ti,ab OR 'probiotic'/exp OR probiotic:ti,ab OR lactobacillus:ti,ab OR bifidobacterium:ti,ab)

**#3 ART terms**

('in vitro fertilization'/exp OR 'in vitro fertilization':ti,ab OR IVF:ti,ab OR 'intracytoplasmic sperm injection'/exp OR 'intracytoplasmic sperm injection':ti,ab OR ICSI:ti,ab OR 'assisted reproductive technique'/exp OR 'assisted reproductive technology':ti,ab OR ART:ti,ab OR 'assisted reproduction':ti,ab OR 'assisted reproductive techniques':ti,ab)

**#4 Pregnancy outcome terms**

('pregnancy outcome'/exp OR 'pregnancy outcome':ti,ab OR 'pregnancy'/exp OR pregnancy:ti,ab OR 'pregnancy rate':ti,ab OR implantation:ti,ab OR 'implantation failure':ti,ab OR 'embryo implantation':ti,ab OR 'live birth':ti,ab OR livebirth:ti,ab OR miscarriage:ti,ab OR 'early pregnancy loss':ti,ab)

**#5 Combine four dimensions**

#1 AND #2 AND #3 AND #4

**#6 Apply time range and language limits**

Limit to English language

Limit to publication year 2000-2025

**#7 Exclude non-original articles**

NOT ('review'/it OR 'systematic review'/it OR 'meta analysis'/it OR 'case report'/it OR 'editorial'/it OR 'letter'/it OR 'conference abstract'/it)

**3.** **Web of science search strategy (Stepwise format)**

**#1 Reproductive tract & follicular fluid**

TS=("reproductive tract" OR vagina OR cervix OR cervical OR uterus OR endometrium OR endometrial OR "follicular fluid" OR "ovarian follicle" OR follicle OR oocyte)

**#2** **Microbiota & probiotics**

TS=(microbiota OR microbiome OR "microbial flora" OR flora OR bacteri* OR dysbiosis OR "microbial composition" OR probiotic OR Lactobacillus OR Bifidobacterium)

**#3 ART terms**

TS=("in vitro fertilization" OR IVF OR "intracytoplasmic sperm injection" OR ICSI OR "assisted reproductive technology" OR ART OR "assisted reproduction" OR "assisted reproductive techniques")

**#4 Pregnancy outcomes**

TS=("pregnancy outcome" OR pregnancy OR "pregnancy rate" OR implantation OR "implantation failure" OR "embryo implantation" OR "live birth" OR livebirth OR miscarriage OR "early pregnancy loss")

**#5 Combine four dimensions**

#1 AND #2 AND #3 AND #4

**#6** **Web of science final search formula**

TS=("reproductive tract" OR vagina OR cervix OR cervical OR uterus OR endometrium OR endometrial OR "follicular fluid" OR "ovarian follicle" OR follicle OR oocyte) AND TS=(microbiota OR microbiome OR "microbial flora" OR flora OR bacteri* OR dysbiosis OR "microbial composition" OR probiotic OR Lactobacillus OR Bifidobacterium) AND TS=("in vitro fertilization" OR IVF OR "intracytoplasmic sperm injection" OR ICSI OR "assisted reproductive technology" OR ART OR "assisted reproduction" OR "assisted reproductive techniques") AND TS=("pregnancy outcome" OR pregnancy OR "pregnancy rate" OR implantation OR "implantation failure" OR "embryo implantation" OR "live birth" OR livebirth OR miscarriage OR "early pregnancy loss")

Publication Years: 2000-2025

Language: English

Document Types: exclude Review, Editorial, Letter, Book Chapter, Meeting Abstract

**4.** **Cochrane library search strategy (Stepwise format)**

**#1 Reproductive tract & follicular fluid**

TS = ("reproductive tract" OR vagina OR cervix OR cervical OR uterus OR endometrium OR endometrial OR "follicular fluid" OR "ovarian follicle" OR follicle OR oocyte)

**#2 Microbiota & probiotics**

TS = (microbiota OR microbiome OR "microbial flora" OR flora OR bacteri* OR dysbiosis OR "microbial composition" OR probiotic OR Lactobacillus OR Bifidobacterium)

**#3 ART terms**

TS = ("in vitro fertilization" OR IVF OR "intracytoplasmic sperm injection" OR ICSI OR "assisted reproductive technology" OR ART OR "assisted reproduction" OR "assisted reproductive techniques")

**#4 Pregnancy outcomes**

TS = ("pregnancy outcome" OR pregnancy OR "pregnancy rate" OR implantation OR "implantation failure" OR "embryo implantation" OR "live birth" OR livebirth OR miscarriage OR "early pregnancy loss")

**#5 Combine four dimensions**

#1 AND #2 AND #3 AND #4

**#6** **Cochrane library final search formula**

("reproductive tract" OR vagina OR cervix OR cervical OR uterus OR endometrium OR endometrial OR "follicular fluid" OR "ovarian follicle" OR follicle OR oocyte) AND (microbiota OR microbiome OR "microbial flora" OR flora OR bacteri* OR dysbiosis OR "microbial composition" OR probiotic OR Lactobacillus OR Bifidobacterium) AND ("in vitro fertilization" OR IVF OR "intracytoplasmic sperm injection" OR ICSI OR "assisted reproductive technology" OR ART OR "assisted reproduction" OR "assisted reproductive techniques") AND ("pregnancy outcome" OR pregnancy OR "pregnancy rate" OR implantation OR "implantation failure" OR "embryo implantation" OR "live birth" OR livebirth OR miscarriage OR "early pregnancy loss")

Publication Years: 2000-2025

Language: English

PublicationTypes: exclude Review, Editorial, Letter, Book Chapter, Meeting Abstract
