# Supplementary material for: Reproductive tract and follicular fluid microbiota and probiotic interventions in IVF/ICSI outcomes: a systematic review
Source: BMC Pregnancy Childbirth. 2026 Jul 4;26:1054. doi: 10.1186/s12884-026-09523-1 (PMC13613614; doi:10.1186/s12884-026-09523-1)
Supplement: Supplementary file 2 [file 12884_2026_9523_MOESM2_ESM.docx]

**Supplementary Table 2. Contamination controls and methodological safeguards for microbiota in low-biomass reproductive tract and follicular fluid samples**

| **Author (Year)** | **Sample type** | **Negative control** | **Extraction blank** | **Sterile kits/Glove changes** | **Decontamination pipeline** | **Overall risk of bias** |
| --- | --- | --- | --- | --- | --- | --- |
| Gilboa et al., 2005 [1] | Vaginal swabs | Not reported | Not reported | Yes | Not reported | Moderate |
| Huerga López et al., 2025 [2] | Vaginal swabs | Yes | Not reported | Yes | Yes | Low |
| Kyono et al., 2019 [3] | Endometrial fluid | Not reported | Not reported | Yes | Not reported | Moderate |
| Di Pierro et al. 2023 [4] | Vaginal swabs and endometrial biopsies | Not reported | Not reported | Yes | Not reported | Moderate |
| Iwami et al. 2023 [5] | Vaginal swabs and endometrial biopsies | Not reported | Not reported | Yes | Not reported | Moderate |
| Bernabeu et al., 2019 [6] | Vaginal swabs | Yes | Not reported | Yes | Yes | Low |
| Hao et al., 2021 [7] | Cervical swabs | Yes | Not reported | Yes | Yes | Low |
| Koort et al., 2023 [8] | Vaginal swabs | Not reported | Not reported | Yes | Yes | Moderate |
| Faisal et al. 2023 [9] | Vaginal swabs | Not reported | Not reported | Yes | Not reported | Moderate |
| Guan et al., 2023 [10] | Cervical swabs | Not reported | Not reported | Yes | Not reported | Moderate |
| Pelzer et al., 2013 [11] | Follicular fluid and vaginal swab | Not reported | Not reported | Yes | Not reported | Moderate |
| Haahr et al., 2016 [12] | Vaginal swab | Not reported | Not reported | Yes | Not reported | Moderate |
| Moreno et al., 2016 [13] | Endometrial fluid and vaginal aspirate | Not reported | Not reported | Yes | Yes | Moderate |
| Moini et al., 2018 [14] | Vaginal swab | Not reported | Not reported | Yes | Not reported | Moderate |
| Hashimoto & Kyono, 2019 [15] | Endometrial fluid | Yes | Yes | Yes | Yes | Low |
| Koedooder et al. 2019 [16] | Vaginal swabs | Not reported | Not reported | Yes | Not reported | Moderate |
| Vergaro et al. 2019 [17] | Vaginal swabs | Not reported | Not reported | Yes | Not reported | Moderate |
| Iwami et al., 2020 [18] | Endometrial tissue | Not reported | Not reported | Yes | Not reported | Moderate |
| Riganelli et al., 2020 [19] | Vaginal and endometrial samples | Yes | Yes | Yes | Yes | Low |
| Kong et al., 2020 [20] | Vaginal secretions | Not reported | Yes | Yes | Yes | Moderate |
| Diaz-Martínez et al., 2021 [21] | Vaginal swabs and endometrial samples | Yes | Not reported | Yes | Yes | Low |
| Usman et al., 2021 [22] | Vaginal swabs and follicular fluid | Not reported | Not reported | Yes | Yes | Moderate |
| Zeng et al., 2021 [23] | Vaginal swabs | Not reported | Not reported | Yes | Yes | Moderate |
| Wang et al. 2021 [24] | Vaginal and cervical canal swabs | Not reported | Not reported | Yes | Yes | Moderate |
| Karaer et al. 2021 [25] | Vaginal swabs | Not reported | Not reported | Yes | Yes | Moderate |
| Kim et al. 2022 [26] | Vaginal swabs | Not reported | Not reported | Yes | Yes | Moderate |
| Ji et al., 2022 [27] | Vaginal swabs | Not reported | Not reported | Yes | Yes | Moderate |
| Villani et al., 2022 [28] | Cervical swabs | Not reported | Not reported | Yes | Yes | Moderate |
| Moreno et al., 2022 [29] | Endometrial fluid aspirate and endometrial biopsy | Yes | Yes | Yes | Yes | Low |
| Ge et al., 2023 [30] | Vaginal secretion swab | Not reported | Not reported | Yes | Yes | Moderate |
| Tong et al., 2023 [31] | Vaginal swabs | Not reported | Not reported | Yes | Yes | Moderate |
| Miyagi et al., 2023 [32] | Vaginal swabs and endometrial secretions | Yes | Not reported | Yes | Yes | Low |
| Cariati et al., 2023 [33] | Endometrial fluid | Not reported | Not reported | Yes | Yes | Moderate |
| Kadogami et al., 2023 [34] | Endometrial tissue | Yes | Not reported | Yes | Yes | Low |
| Väinämö et al., 2023 [35] | Vaginal swabs | Not reported | Not reported | Yes | Yes | Moderate |
| Bui et al., 2023 [36] | Endometrial tissue | Yes | Yes | Yes | Yes | Low |
| Nishio et al., 2024 [37] | Cervicovaginal mucus | Not reported | Not reported | Yes | Yes | Moderate |
| Wu et al., 2025 [38] | Cervical mucus | Not reported | Not reported | Yes | Yes | Moderate |
| Bar et al., 2025 [39] | Vaginal swabs | Not reported | Not reported | Yes | Yes | Moderate |
| Alley et al. 2025 [40] | Vaginal swabs | Yes | Yes | Yes | Yes | Low |

**References**

1. Gilboa Y, Bar-Hava, I, Fisch, B, Ashkenazi, J, Voliovitch, I, Borkowski, T, Orvieto R. Does intravaginal probiotic supplementation increase the pregnancy rate in IVF-embryo transfer cycles? Reproductive biomedicine online. 2005;11:71‐75. https://doi.org/10.1016/s1472-6483(10)61301-6.

2. Huerga López C, Sánchez Martín, MJ, Herráez Moreta, A, Calvo Urrutia, M, Cristóbal García, I, Díaz Morillo, C, Blanco-Rojo, R, Sáez, ME, Olivares, M, Arroyo, R, Herranz, C, Alba, C, Rodríguez, JM, Fernández L. Ligilactobacillus salivarius CECT5713 Increases Term Pregnancies in Women with Infertility of Unknown Origin: a Randomized, Triple-Blind, Placebo-Controlled Trial. Nutrients. 2025;17. https://doi.org/10.3390/nu17111860.

3. Kyono K, Hashimoto T, Kikuchi S, Nagai Y, Sakuraba Y. A pilot study and case reports on endometrial microbiota and pregnancy outcome: An analysis using 16S rRNA gene sequencing among IVF patients, and trial therapeutic intervention for dysbiotic endometrium. Reprod Med Biol. 2019;18:72–82. https://doi.org/10.1002/rmb2.12250.

4. Di Pierro F, Sinatra F, Cester M, Da Ros L, Pistolato M, Da Parè V, et al. Effect of L. crispatus M247 Administration on Pregnancy Outcomes in Women Undergoing IVF: A Controlled, Retrospective, Observational, and Open-Label Study. MICROORGANISMS. 2023;11. https://doi.org/10.3390/microorganisms11112796.

5. Iwami N, Kawamata M, Ozawa N, Yamamoto T, Watanabe E, Mizuuchi M, et al. Therapeutic intervention based on gene sequencing analysis of microbial 16S ribosomal RNA of the intrauterine microbiome improves pregnancy outcomes in IVF patients: a prospective cohort study. J Assist Reprod Genet. 2023;40:125–35. https://doi.org/10.1007/s10815-022-02688-6.

6. Bernabeu A, Lledo B, Díaz MC, Lozano FM, Ruiz V, Fuentes A, et al. Effect of the vaginal microbiome on the pregnancy rate in women receiving assisted reproductive treatment. J Assist Reprod Genet. 2019;36:2111–9. https://doi.org/10.1007/s10815-019-01564-0.

7. Hao X, Li P, Wu S, Tan J. Association of the Cervical Microbiota With Pregnancy Outcome in a Subfertile Population Undergoing In Vitro Fertilization: A Case-Control Study. Front Cell Infect Microbiol. 2021;11. https://doi.org/10.3389/fcimb.2021.654202.

8. Koort K, Sõsa K, Türk S, Lapp E, Talving E, Karits P, et al. Lactobacillus crispatus-dominated vaginal microbiome and Acinetobacter-dominated seminal microbiome support beneficial ART outcome. Acta Obstet Gynecol Scand. 2023;102:921–34. https://doi.org/10.1111/aogs.14598.

9. Farooq Faisal S, Adnan Abdul Hameed W, Alwasiti E. The Influence of Vaginal Dysbiosis on Intracytoplasmic Sperm Injection Outcome. Arch Razi Inst. 2023;78:227–32. https://doi.org/10.22092/ARI.2022.358610.2260.

10. Guan W, Dong S, Wang Z, Jiao J, Wang X. Impact of a Lactobacillus dominant cervical microbiome, based on 16S-FAST profiling, on the reproductive outcomes of IVF patients. Front Cell Infect Microbiol. 2023;13:1059339. https://doi.org/10.3389/fcimb.2023.1059339.

11. Pelzer ES, Allan JA, Waterhouse MA, Ross T, Beagley KW, Knox CL. Microorganisms within human follicular fluid: effects on IVF. PLoS One. 2013;8:e59062. https://doi.org/10.1371/journal.pone.0059062.

12. Haahr T, Jensen JS, Thomsen L, Duus L, Rygaard K, Humaidan P. Abnormal vaginal microbiota may be associated with poor reproductive outcomes: a prospective study in IVF patients. Hum Reprod. 2016;31:795–803. https://doi.org/10.1093/humrep/dew026.

13. Moreno I, Codoñer FM, Vilella F, Valbuena D, Martinez-Blanch JF, Jimenez-Almazán J, et al. Evidence that the endometrial microbiota has an effect on implantation success or failure. Am J Obstet Gynecol. 2016;215:684–703. https://doi.org/10.1016/j.ajog.2016.09.075.

14. Moini A, Mohammadi Yeganeh L, Shiva M, Ahmadieh M, Salman Yazdi R, Hasani F, et al. Bacterial vaginosis and the risk of early miscarriage in women undergoing intracytoplasmic sperm injection cycles: a prospective cohort study. Hum Fertil (Camb). 2018;21:263–8. https://doi.org/10.1080/14647273.2017.1353709.

15. Hashimoto T, Kyono K. Does dysbiotic endometrium affect blastocyst implantation in IVF patients? J Assist Reprod Genet. 2019;36:2471–9. https://doi.org/10.1007/s10815-019-01630-7.

16. Koedooder R, Singer M, Schoenmakers S, Savelkoul PHM, Morré SA, de Jonge JD, et al. The vaginal microbiome as a predictor for outcome of in vitro fertilization with or without intracytoplasmic sperm injection: a prospective study. Hum Reprod. 2019;34:1042–54. https://doi.org/10.1093/humrep/dez065.

17. Vergaro P, Tiscornia G, Barragán M, García D, Rodriguez A, Santaló J, et al. Vaginal microbiota profile at the time of embryo transfer does not affect live birth rate in IVF cycles with donated oocytes. Reprod Biomed Online. 2019;38:883–91. https://doi.org/10.1016/j.rbmo.2018.12.019.

18. Iwami N, Kawamata, M, Ozawa, N, Yamamoto, T, Watanabe, E, Mizuuchi, M, Moriwaka, O, Kamiya H. Efficacy of endometrial microbiome metagenomic analysis and analysis of infectious chronic endometritis on in vitro fertilization outcome in women with recurrent implantation failure. Human reproduction (Oxford, England). 2020;35 SUPPL 1:i31.

19. Riganelli L, Iebba V, Piccioni M, Illuminati I, Bonfiglio G, Neroni B, et al. Structural Variations of Vaginal and Endometrial Microbiota: Hints on Female Infertility. FRONTIERS IN CELLULAR AND INFECTION MICROBIOLOGY. 2020;10. https://doi.org/10.3389/fcimb.2020.00350.

20. Kong Y, Liu Z, Shang Q, Gao Y, Li X, Zheng C, et al. The Disordered Vaginal Microbiota Is a Potential Indicator for a Higher Failure of in vitro Fertilization. Front Med (Lausanne). 2020;7:217. https://doi.org/10.3389/fmed.2020.00217.

21. Diaz-Martínez MDC, Bernabeu A, Lledó B, Carratalá-Munuera C, Quesada JA, Lozano FM, et al. Impact of the Vaginal and Endometrial Microbiome Pattern on Assisted Reproduction Outcomes. J Clin Med. 2021;10. https://doi.org/10.3390/jcm10184063.

22. Usman SF, Shuaibu IR, Durojaiye K, Medugu N, Iregbu KC. The presence of microorganisms in follicular fluid and its effect on the outcome of in vitro fertilization-embryo transfer (IVF-ET) treatment cycles. PLoS One. 2021;16:e0246644. https://doi.org/10.1371/journal.pone.0246644.

23. Zeng H, He D, Hu L, Abdullah RK, Zhang L, Jiang B, et al. Non-Lactobacillus dominance of the vagina is associated with reduced live birth rate following IVF/ICSI: a propensity score-matched cohort study. Arch Gynecol Obstet. 2022;305:519–28. https://doi.org/10.1007/s00404-021-06171-y.

24. Wang R, Zhou G, Wu L, Huang X, Li Y, Luo B, et al. The Microbial Composition of Lower Genital Tract May Affect the Outcome of in vitro Fertilization-Embryo Transfer. Front Microbiol. 2021;12:729744. https://doi.org/10.3389/fmicb.2021.729744.

25. Karaer A, Doğan B, Günal S, Tuncay G, Arda Düz S, Ünver T, et al. The vaginal microbiota composition of women undergoing assisted reproduction: a prospective cohort study. BJOG. 2021;128:2101–9. https://doi.org/10.1111/1471-0528.16782.

26. Kim SM, Won KH, Hong YH, Kim SK, Lee JR, Jee BC, et al. Microbiology of Human Follicular Fluid and the Vagina and Its Impact on in Vitro Fertilization Outcomes. Yonsei Med J. 2022;63:941–7. https://doi.org/10.3349/ymj.2022.0190.

27. Ji L, Peng C, Bao X. Effect of vaginal flora on clinical outcome of frozen embryo transfer. Front Cell Infect Microbiol. 2022;12:987292. https://doi.org/10.3389/fcimb.2022.987292.

28. Villani A, Fontana A, Barone S, de Stefani S, Primiterra M, Copetti M, et al. Identifying Predictive Bacterial Markers from Cervical Swab Microbiota on Pregnancy Outcome in Woman Undergoing Assisted Reproductive Technologies. J Clin Med. 2022;11. https://doi.org/10.3390/jcm11030680.

29. Moreno I., Garcia-Grau I., Perez-Villaroya D., Gonzalez-Monfort M., Bahceci M., Barrionuevo M.J., et al. Endometrial microbiota composition is associated with reproductive outcome in infertile patients. Microbiome. 2022;10:1. https://doi.org/10.1186/s40168-021-01184-w.

30. Ge Y-M, Lu J-C, Xu Y-H, Tang S-S, Zhi S-S, Liang Y-J. Correlations of joint detection of 22 vaginal microbes with routine examination results of vaginal secretions and assisted reproductive outcomes. Diagn Microbiol Infect Dis. 2023;106:115940. https://doi.org/10.1016/j.diagmicrobio.2023.115940.

31. Tong Y, Sun Q, Shao X, Wang Z. Effect of vaginal microbiota on pregnancy outcomes of women from Northern China who conceived after IVF. Front Endocrinol (Lausanne). 2023;14:1200002. https://doi.org/10.3389/fendo.2023.1200002.

32. Miyagi M, Mekaru K, Tanaka SE, Arai W, Ashikawa K, Sakuraba Y, et al. Endometrial and vaginal microbiomes influence assisted reproductive technology outcomes. JBRA Assist Reprod. 2023;27:267–81. https://doi.org/10.5935/1518-0557.20220040.

33. Cariati F, Carotenuto C, Bagnulo F, Pacella D, Marrone V, Paolillo R, et al. Endometrial microbiota profile in in-vitro fertilization (IVF) patients by culturomics-based analysis. Front Endocrinol (Lausanne). 2023;14:1204729. https://doi.org/10.3389/fendo.2023.1204729.

34. Kadogami D, Kimura F, Hanada T, Tsuji S, Nakaoka Y, Murakami T, et al. Impact of Lactobacillus in the uterine microbiota on in vitro fertilization outcomes. J Reprod Immunol. 2023;160:104138. https://doi.org/10.1016/j.jri.2023.104138.

35. Väinämö S, Saqib S, Kalliala I, Kervinen K, Luiro K, Niinimäki M, et al. Longitudinal analysis of vaginal microbiota during IVF fresh embryo transfer and in early pregnancy. Microbiol Spectr. 2023;11:e0165023. https://doi.org/10.1128/spectrum.01650-23.

36. Bui BN, van Hoogenhuijze N, Viveen M, Mol F, Teklenburg G, de Bruin J-P, et al. The endometrial microbiota of women with or without a live birth within 12 months after a first failed IVF/ICSI cycle. Sci Rep. 2023;13:3444. https://doi.org/10.1038/s41598-023-30591-2.

37. Nishio E, Iwata A, Kawasaki R, Iwao K, Nishizawa H, Fujii T. Metabolomic and microbiome analysis of cervicovaginal mucus in in vitro fertilization-embryo transfer: Toward predicting pregnancy success. Reprod Med Biol. 2024;23:e12568. https://doi.org/10.1002/rmb2.12568.

38. Wu Y, Shi L, Jin Z, Chen W, Wang F, Wu H, et al. A nomogram prediction model for embryo implantation outcomes based on the cervical microbiota of the infertile patients during IVF-FET. Microbiol Spectr. 2025;13:e0146224. https://doi.org/10.1128/spectrum.01462-24.

39. Bar O, Vagios S, Barkai O, Elshirbini J, Souter I, Xu J, et al. Harnessing vaginal inflammation and microbiome: a machine learning model for predicting IVF success. NPJ Biofilms Microbiomes. 2025;11:95. https://doi.org/10.1038/s41522-025-00732-8.

40. Alley AW, Łaniewski P, Bruno GT, Moffitt DV, Arani G, Farland LV, et al. Vaginal Microbiome Dominated by Lactobacillus Positively Impacts Clinical Pregnancy in Patients With Frozen Embryo Transfers. Am J Reprod Immunol. 2025;93:e70108. https://doi.org/10.1111/aji.70108.
