# Supplementary material for: Reproductive tract and follicular fluid microbiota and probiotic interventions in IVF/ICSI outcomes: a systematic review
Source: BMC Pregnancy Childbirth. 2026 Jul 4;26:1054. doi: 10.1186/s12884-026-09523-1 (PMC13613614; doi:10.1186/s12884-026-09523-1)
Supplement: Supplementary file 3 [file 12884_2026_9523_MOESM3_ESM.docx]

**Risk of bias assessment for RCTs (RoB 2.0)**

| **Study (Year)** | **Randomization process** | **Deviations from intended interventions** | **Missing outcome data** | **Measurement of outcome** | **Selection of reported result** | **Overall risk of bias** |
| --- | --- | --- | --- | --- | --- | --- |
| Gilboa et al., 2005 [1] | Low | Low | Low | Low | Moderate | Low |
| Huerga López et al., 2025 [2] | Low | Low | Moderate | Low | Moderate | Moderate |

Risk of bias was independently assessed by two reviewers, with discrepancies resolved through discussion or consultation with a third reviewer. Each domain and the overall risk were rated according to the Cochrane RoB 2.0 tool as Low risk of bias, Some concerns, or High risk of bias. For clarity in presentation, “Some concerns” was reported as Moderate risk of bias in the table.

**Risk of bias assessment of non-randomized interventional studies (ROBINS-I)**

| **Study (Year)** | **Confounding** | **Selection of participants** | **Classification of intervention** | **Deviations from intended interventions** | **Missing data** | **Risk of bias in measurement** | **Selective reporting** | **Overall Risk of bias** |
| --- | --- | --- | --- | --- | --- | --- | --- | --- |
| Kyono et al., 2019 [3] | High | Moderate | Low | Moderate | Low | Low | Low | High |
| Di Pierro et al. 2023 [4] | High | Moderate | Low | Moderate | Low | Low | Low | High |
| Iwami et al. 2023 [5] | Moderate | Moderate | Low | Low | Low | Low | Low | Moderate |

Risk of bias was independently assessed by two reviewers, with discrepancies resolved through discussion or consultation with a third reviewer. Each domain and the overall risk were rated according to the ROBINS-I tool as Low, Moderate, Serious, Critical, or No information. For simplicity and consistency in presentation, “Low” and “Moderate” ratings were retained, while “Serious” or “Critical” ratings were classified as High.

**Risk of bias assessment for** **case-control studies (JBI)**

| **Study (Year)** | **Case definition clearly described** | **Control selection appropriate** | **Matching appropriate** | **Exposure measurement valid** | **Confounding identified** | **Confounding controlled** | **Statistical analysis appropriate** | **Overall risk of bias** |
| --- | --- | --- | --- | --- | --- | --- | --- | --- |
| Bernabeu et al., 2019 [6] | Low | Unclear | High | Low | Moderate | High | Low | Moderate |
| Hao et al., 2021 [7] | Low | Low | Moderate | Low | Low | Low | Low | Low |

Risk of bias was independently assessed by two reviewers, with discrepancies resolved through discussion or consultation with a third reviewer. For case‑control studies, each domain and the overall risk were initially rated as Yes, No, or Unclear according to the JBI Critical Appraisal Checklist for Case‑Control Studies. Subsequently, based on the proportion of “Yes” responses, studies were classified into three risk levels: Low (≥ 70% yes), Moderate (50–69% yes), and High (< 50% yes) for clarity.

**Risk of bias assessment for** **cross-sectional studies (JBI)**

| **Study (Year)** | **Inclusion criteria clear** | **Exposure measurement valid** | **Outcome measurement valid** | **Confounding identified** | **Confounding controlled** | **Statistical analysis appropriate** | **Overall risk of bias** |
| --- | --- | --- | --- | --- | --- | --- | --- |
| Koort et al., 2023 [8] | Low | Low | Low | Low | Moderate | Low | Moderate |
| Faisal et al. 2023 [9] | Low | Low | Low | Moderate | Moderate | Low | Moderate |
| Guan et al., 2023 [10] | Low | Low | Low | Moderate | Moderate | Low | Moderate |

Risk of bias was independently assessed by two reviewers, with discrepancies resolved through discussion or consultation with a third reviewer. For cross-sectional studies, each domain and the overall risk were initially rated as Yes, No, or Unclear according to the JBI Critical Appraisal Checklist for Cross-sectional Studies. Subsequently, based on the proportion of “Yes” responses, studies were classified into three risk levels: Low (≥ 70% yes), Moderate (50–69% yes), and High (< 50% yes) for clarity.

**Risk of bias assessment for** **cohort studies (JBI)**

| **Study (Year)** | **Selection of participants** | **Exposure measurement valid** | **Outcome measurement valid** | **Confounding identified** | **Confounding controlled** | **Follow-up completeness** | **Statistical analysis appropriate** | **Overall Risk of bias** |
| --- | --- | --- | --- | --- | --- | --- | --- | --- |
| Pelzer et al., 2013 [11] | Low | Low | Low | Moderate | Low | Low | Low | Moderate |
| Haahr et al., 2016 [12] | Moderate | Low | Moderate | Low | Moderate | Low | Low | Moderate |
| Moreno et al., 2016 [13] | Moderate | Low | Low | Moderate | Low | Low | Low | Low |
| Moini et al., 2018 [14] | Low | Low | Low | Moderate | Moderate | Low | Low | Moderate |
| Hashimoto & Kyono, 2019 [15] | Low | Low | Low | Moderate | Moderate | Low | Low | Moderate |
| Koedooder et al. 2019 [16] | Low | Low | Low | Moderate | Moderate | Low | Low | Moderate |
| Vergaro et al. 2019 [17] | Low | Low | Low | High | Moderate | Low | Moderate | Moderate |
| Iwami et al., 2020 [18] | Low | Moderate | Moderate | Low | High | Low | Low | High |
| Riganelli et al., 2020 [19] | Low | Low | Low | Moderate | Moderate | Low | Moderate | Moderate |
| Kong et al., 2020 [20] | Low | Low | Low | Moderate | Low | Low | Low | Moderate |
| Diaz-Martínez et al., 2021 [21] | Low | Low | Low | Moderate | Moderate | Low | Low | Moderate |
| Usman et al., 2021 [22] | Low | Low | Low | Low | Low | Low | Low | Low |
| Zeng et al., 2021 [23] | Low | Low | Moderate | Low | Low | Low | Moderate | Moderate |
| Wang et al. 2021 [24] | Low | Low | Low | High | Low | Low | Moderate | High |
| Karaer et al. 2021 [25] | Low | Low | Low | Low | Low | Low | Moderate | Moderate |
| Kim et al. 2022 [26] | Low | Low | Low | Low | Low | Low | Low | Low |
| Ji et al., 2022 [27] | Low | Low | Low | Moderate | Low | Low | Low | Moderate |
| Villani et al., 2022 [28] | Moderate | Low | Low | Low | Moderate | Low | Low | Moderate |
| Moreno et al., 2022 [29] | Low | Low | Low | Moderate | Low | Low | Moderate | Moderate |
| Ge et al., 2023 [30] | Moderate | Moderate | Low | Moderate | High | Moderate | Moderate | Moderate |
| Tong et al., 2023 [31] | Low | Low | Low | High | Low | Low | Low | High |
| Miyagi et al., 2023 [32] | Low | Moderate | Low | Moderate | Low | Low | Low | Moderate |
| Cariati et al., 2023 [33] | Low | Moderate | Low | High | Moderate | High | Moderate | High |
| Kadogami et al., 2023 [34] | Low | Low | Low | Moderate | Moderate | Low | Low | Moderate |
| Väinämö et al., 2023 [35] | Low | Low | Low | Moderate | Moderate | High | Moderate | Moderate |
| Bui et al., 2023 [36] | Low | Low | Low | Moderate | Low | Moderate | Low | Moderate |
| Nishio et al., 2024 [37] | Low | Moderate | Low | Moderate | Moderate | High | Moderate | Moderate |
| Wu et al., 2025 [38] | Low | Low | Low | Moderate | Moderate | High | Moderate | Moderate |
| Bar et al., 2025 [39] | Low | Low | Low | Moderate | Moderate | Low | Low | Moderate |
| Alley et al. 2025 [40] | Low | Low | Low | Moderate | Moderate | Low | Low | Moderate |

Risk of bias was independently assessed by two reviewers, with discrepancies resolved through discussion or consultation with a third reviewer. For cohort studies, each domain and the overall risk were initially rated as Yes, No, or Unclear according to the JBI Critical Appraisal Checklist for Cohort Studies. Subsequently, based on the proportion of “Yes” responses, studies were classified into three risk levels: Low (≥ 70% yes), Moderate (50–69% yes), and High (< 50% yes) for clarity.

**References**

1. Gilboa Y, Bar-Hava, I, Fisch, B, Ashkenazi, J, Voliovitch, I, Borkowski, T, Orvieto R. Does intravaginal probiotic supplementation increase the pregnancy rate in IVF-embryo transfer cycles? Reproductive biomedicine online. 2005;11:71‐75. https://doi.org/10.1016/s1472-6483(10)61301-6.

2. Huerga López C, Sánchez Martín, MJ, Herráez Moreta, A, Calvo Urrutia, M, Cristóbal García, I, Díaz Morillo, C, Blanco-Rojo, R, Sáez, ME, Olivares, M, Arroyo, R, Herranz, C, Alba, C, Rodríguez, JM, Fernández L. Ligilactobacillus salivarius CECT5713 Increases Term Pregnancies in Women with Infertility of Unknown Origin: a Randomized, Triple-Blind, Placebo-Controlled Trial. Nutrients. 2025;17. https://doi.org/10.3390/nu17111860.

3. Kyono K, Hashimoto T, Kikuchi S, Nagai Y, Sakuraba Y. A pilot study and case reports on endometrial microbiota and pregnancy outcome: An analysis using 16S rRNA gene sequencing among IVF patients, and trial therapeutic intervention for dysbiotic endometrium. Reprod Med Biol. 2019;18:72–82. https://doi.org/10.1002/rmb2.12250.

4. Di Pierro F, Sinatra F, Cester M, Da Ros L, Pistolato M, Da Parè V, et al. Effect of L. crispatus M247 Administration on Pregnancy Outcomes in Women Undergoing IVF: A Controlled, Retrospective, Observational, and Open-Label Study. MICROORGANISMS. 2023;11. https://doi.org/10.3390/microorganisms11112796.

5. Iwami N, Kawamata M, Ozawa N, Yamamoto T, Watanabe E, Mizuuchi M, et al. Therapeutic intervention based on gene sequencing analysis of microbial 16S ribosomal RNA of the intrauterine microbiome improves pregnancy outcomes in IVF patients: a prospective cohort study. J Assist Reprod Genet. 2023;40:125–35. https://doi.org/10.1007/s10815-022-02688-6.

6. Bernabeu A, Lledo B, Díaz MC, Lozano FM, Ruiz V, Fuentes A, et al. Effect of the vaginal microbiome on the pregnancy rate in women receiving assisted reproductive treatment. J Assist Reprod Genet. 2019;36:2111–9. https://doi.org/10.1007/s10815-019-01564-0.

7. Hao X, Li P, Wu S, Tan J. Association of the Cervical Microbiota With Pregnancy Outcome in a Subfertile Population Undergoing In Vitro Fertilization: A Case-Control Study. Front Cell Infect Microbiol. 2021;11. https://doi.org/10.3389/fcimb.2021.654202.

8. Koort K, Sõsa K, Türk S, Lapp E, Talving E, Karits P, et al. Lactobacillus crispatus-dominated vaginal microbiome and Acinetobacter-dominated seminal microbiome support beneficial ART outcome. Acta Obstet Gynecol Scand. 2023;102:921–34. https://doi.org/10.1111/aogs.14598.

9. Farooq Faisal S, Adnan Abdul Hameed W, Alwasiti E. The Influence of Vaginal Dysbiosis on Intracytoplasmic Sperm Injection Outcome. Arch Razi Inst. 2023;78:227–32. https://doi.org/10.22092/ARI.2022.358610.2260.

10. Guan W, Dong S, Wang Z, Jiao J, Wang X. Impact of a Lactobacillus dominant cervical microbiome, based on 16S-FAST profiling, on the reproductive outcomes of IVF patients. Front Cell Infect Microbiol. 2023;13:1059339. https://doi.org/10.3389/fcimb.2023.1059339.

11. Pelzer ES, Allan JA, Waterhouse MA, Ross T, Beagley KW, Knox CL. Microorganisms within human follicular fluid: effects on IVF. PLoS One. 2013;8:e59062. https://doi.org/10.1371/journal.pone.0059062.

12. Haahr T, Jensen JS, Thomsen L, Duus L, Rygaard K, Humaidan P. Abnormal vaginal microbiota may be associated with poor reproductive outcomes: a prospective study in IVF patients. Hum Reprod. 2016;31:795–803. https://doi.org/10.1093/humrep/dew026.

13. Moreno I, Codoñer FM, Vilella F, Valbuena D, Martinez-Blanch JF, Jimenez-Almazán J, et al. Evidence that the endometrial microbiota has an effect on implantation success or failure. Am J Obstet Gynecol. 2016;215:684–703. https://doi.org/10.1016/j.ajog.2016.09.075.

14. Moini A, Mohammadi Yeganeh L, Shiva M, Ahmadieh M, Salman Yazdi R, Hasani F, et al. Bacterial vaginosis and the risk of early miscarriage in women undergoing intracytoplasmic sperm injection cycles: a prospective cohort study. Hum Fertil (Camb). 2018;21:263–8. https://doi.org/10.1080/14647273.2017.1353709.

15. Hashimoto T, Kyono K. Does dysbiotic endometrium affect blastocyst implantation in IVF patients? J Assist Reprod Genet. 2019;36:2471–9. https://doi.org/10.1007/s10815-019-01630-7.

16. Koedooder R, Singer M, Schoenmakers S, Savelkoul PHM, Morré SA, de Jonge JD, et al. The vaginal microbiome as a predictor for outcome of in vitro fertilization with or without intracytoplasmic sperm injection: a prospective study. Hum Reprod. 2019;34:1042–54. https://doi.org/10.1093/humrep/dez065.

17. Vergaro P, Tiscornia G, Barragán M, García D, Rodriguez A, Santaló J, et al. Vaginal microbiota profile at the time of embryo transfer does not affect live birth rate in IVF cycles with donated oocytes. Reprod Biomed Online. 2019;38:883–91. https://doi.org/10.1016/j.rbmo.2018.12.019.

18. Iwami N, Kawamata, M, Ozawa, N, Yamamoto, T, Watanabe, E, Mizuuchi, M, Moriwaka, O, Kamiya H. Efficacy of endometrial microbiome metagenomic analysis and analysis of infectious chronic endometritis on in vitro fertilization outcome in women with recurrent implantation failure. Human reproduction (Oxford, England). 2020;35 SUPPL 1:i31.

19. Riganelli L, Iebba V, Piccioni M, Illuminati I, Bonfiglio G, Neroni B, et al. Structural Variations of Vaginal and Endometrial Microbiota: Hints on Female Infertility. FRONTIERS IN CELLULAR AND INFECTION MICROBIOLOGY. 2020;10. https://doi.org/10.3389/fcimb.2020.00350.

20. Kong Y, Liu Z, Shang Q, Gao Y, Li X, Zheng C, et al. The Disordered Vaginal Microbiota Is a Potential Indicator for a Higher Failure of in vitro Fertilization. Front Med (Lausanne). 2020;7:217. https://doi.org/10.3389/fmed.2020.00217.

21. Diaz-Martínez MDC, Bernabeu A, Lledó B, Carratalá-Munuera C, Quesada JA, Lozano FM, et al. Impact of the Vaginal and Endometrial Microbiome Pattern on Assisted Reproduction Outcomes. J Clin Med. 2021;10. https://doi.org/10.3390/jcm10184063.

22. Usman SF, Shuaibu IR, Durojaiye K, Medugu N, Iregbu KC. The presence of microorganisms in follicular fluid and its effect on the outcome of in vitro fertilization-embryo transfer (IVF-ET) treatment cycles. PLoS One. 2021;16:e0246644. https://doi.org/10.1371/journal.pone.0246644.

23. Zeng H, He D, Hu L, Abdullah RK, Zhang L, Jiang B, et al. Non-Lactobacillus dominance of the vagina is associated with reduced live birth rate following IVF/ICSI: a propensity score-matched cohort study. Arch Gynecol Obstet. 2022;305:519–28. https://doi.org/10.1007/s00404-021-06171-y.

24. Wang R, Zhou G, Wu L, Huang X, Li Y, Luo B, et al. The Microbial Composition of Lower Genital Tract May Affect the Outcome of in vitro Fertilization-Embryo Transfer. Front Microbiol. 2021;12:729744. https://doi.org/10.3389/fmicb.2021.729744.

25. Karaer A, Doğan B, Günal S, Tuncay G, Arda Düz S, Ünver T, et al. The vaginal microbiota composition of women undergoing assisted reproduction: a prospective cohort study. BJOG. 2021;128:2101–9. https://doi.org/10.1111/1471-0528.16782.

26. Kim SM, Won KH, Hong YH, Kim SK, Lee JR, Jee BC, et al. Microbiology of Human Follicular Fluid and the Vagina and Its Impact on in Vitro Fertilization Outcomes. Yonsei Med J. 2022;63:941–7. https://doi.org/10.3349/ymj.2022.0190.

27. Ji L, Peng C, Bao X. Effect of vaginal flora on clinical outcome of frozen embryo transfer. Front Cell Infect Microbiol. 2022;12:987292. https://doi.org/10.3389/fcimb.2022.987292.

28. Villani A, Fontana A, Barone S, de Stefani S, Primiterra M, Copetti M, et al. Identifying Predictive Bacterial Markers from Cervical Swab Microbiota on Pregnancy Outcome in Woman Undergoing Assisted Reproductive Technologies. J Clin Med. 2022;11. https://doi.org/10.3390/jcm11030680.

29. Moreno I., Garcia-Grau I., Perez-Villaroya D., Gonzalez-Monfort M., Bahceci M., Barrionuevo M.J., et al. Endometrial microbiota composition is associated with reproductive outcome in infertile patients. Microbiome. 2022;10:1. https://doi.org/10.1186/s40168-021-01184-w.

30. Ge Y-M, Lu J-C, Xu Y-H, Tang S-S, Zhi S-S, Liang Y-J. Correlations of joint detection of 22 vaginal microbes with routine examination results of vaginal secretions and assisted reproductive outcomes. Diagn Microbiol Infect Dis. 2023;106:115940. https://doi.org/10.1016/j.diagmicrobio.2023.115940.

31. Tong Y, Sun Q, Shao X, Wang Z. Effect of vaginal microbiota on pregnancy outcomes of women from Northern China who conceived after IVF. Front Endocrinol (Lausanne). 2023;14:1200002. https://doi.org/10.3389/fendo.2023.1200002.

32. Miyagi M, Mekaru K, Tanaka SE, Arai W, Ashikawa K, Sakuraba Y, et al. Endometrial and vaginal microbiomes influence assisted reproductive technology outcomes. JBRA Assist Reprod. 2023;27:267–81. https://doi.org/10.5935/1518-0557.20220040.

33. Cariati F, Carotenuto C, Bagnulo F, Pacella D, Marrone V, Paolillo R, et al. Endometrial microbiota profile in in-vitro fertilization (IVF) patients by culturomics-based analysis. Front Endocrinol (Lausanne). 2023;14:1204729. https://doi.org/10.3389/fendo.2023.1204729.

34. Kadogami D, Kimura F, Hanada T, Tsuji S, Nakaoka Y, Murakami T, et al. Impact of Lactobacillus in the uterine microbiota on in vitro fertilization outcomes. J Reprod Immunol. 2023;160:104138. https://doi.org/10.1016/j.jri.2023.104138.

35. Väinämö S, Saqib S, Kalliala I, Kervinen K, Luiro K, Niinimäki M, et al. Longitudinal analysis of vaginal microbiota during IVF fresh embryo transfer and in early pregnancy. Microbiol Spectr. 2023;11:e0165023. https://doi.org/10.1128/spectrum.01650-23.

36. Bui BN, van Hoogenhuijze N, Viveen M, Mol F, Teklenburg G, de Bruin J-P, et al. The endometrial microbiota of women with or without a live birth within 12 months after a first failed IVF/ICSI cycle. Sci Rep. 2023;13:3444. https://doi.org/10.1038/s41598-023-30591-2.

37. Nishio E, Iwata A, Kawasaki R, Iwao K, Nishizawa H, Fujii T. Metabolomic and microbiome analysis of cervicovaginal mucus in in vitro fertilization-embryo transfer: Toward predicting pregnancy success. Reprod Med Biol. 2024;23:e12568. https://doi.org/10.1002/rmb2.12568.

38. Wu Y, Shi L, Jin Z, Chen W, Wang F, Wu H, et al. A nomogram prediction model for embryo implantation outcomes based on the cervical microbiota of the infertile patients during IVF-FET. Microbiol Spectr. 2025;13:e0146224. https://doi.org/10.1128/spectrum.01462-24.

39. Bar O, Vagios S, Barkai O, Elshirbini J, Souter I, Xu J, et al. Harnessing vaginal inflammation and microbiome: a machine learning model for predicting IVF success. NPJ Biofilms Microbiomes. 2025;11:95. https://doi.org/10.1038/s41522-025-00732-8.

40. Alley AW, Łaniewski P, Bruno GT, Moffitt DV, Arani G, Farland LV, et al. Vaginal Microbiome Dominated by Lactobacillus Positively Impacts Clinical Pregnancy in Patients With Frozen Embryo Transfers. Am J Reprod Immunol. 2025;93:e70108. https://doi.org/10.1111/aji.70108.
