# Supplementary material for: Reproductive tract and follicular fluid microbiota and probiotic interventions in IVF/ICSI outcomes: a systematic review
Source: BMC Pregnancy Childbirth. 2026 Jul 4;26:1054. doi: 10.1186/s12884-026-09523-1 (PMC13613614; doi:10.1186/s12884-026-09523-1)
Supplement: Supplementary file 4 [file 12884_2026_9523_MOESM4_ESM.docx]

**Supplementary Table 4. Comparison of laboratory and bioinformatic methods across included studies**

| **Study (Year)** | **Sample site & timing** | **Storage & transport** | **DNA extraction kit** | **16S region** | **OTU/ASV** | **Sequencing depth (reads/sample)** | **Rarefaction/filtering** | **Taxonomy database** | **Negative controls** | **qPCR validation** | **Normalization & statistics** | **Operational definition of *Lactobacillus* dominance** | **Direction of association with primary outcome** |
| --- | --- | --- | --- | --- | --- | --- | --- | --- | --- | --- | --- | --- | --- |
| Gilboa et al., 2005 [1] | Vaginal swabs: before oocyte retrieval and at embryo transfer | Swabs transferred for culture or rolled on slide for Gram staining; vaginal washing before retrieval | NR | NR | NR | NR | NR | NR | NR | NR | Student’s t-test and Fisher’s exact test; results as mean ± SD; P<0.05 considered significant | Not formally defined; study evaluated vaginal flora by culture and Gram stain rather than sequencing-based *Lactobacillus* dominance classification. | No significant association between *Lactobacillus* enrichment and pregnancy outcome. |
| Huerga López et al., 2025 [2] | Vaginal swabs and semen samples: time 1 (before intervention) and time 2 (before IVF or upon pregnancy) | Samples stored at -20 °C at hospital, transported under temperature-controlled conditions to lab, then stored at −80 °C until analysis | QIAamp DNA Stool Mini Kit (Qiagen) with mechanical lysis, RNAse A and proteinase K treatment | V-V4 | OTUs (DADA2) | NR (Illumina MiSeq, paired-end) | Quality filtering and merging in DADA2; post-sequencing QC in QIIME 2022.2 | SILVA SSU 138.1 | Included in all PCR reactions | qPCR for L. salivarius (16S-23S intergenic spacer) | Alpha diversity: Shannon & Simpson; Beta diversity: PERMANOVA on Bray-Curtis & Jaccard; ANOVA, t-test, Wilcoxon tests, Chi-square/Fisher; R 4.1.0 & StatGraphics Centurion 19 | Not formally defined by a specific *Lactobacillus* abundance threshold; microbiota composition assessed using metataxonomic profiling and targeted qPCR for *Ligilactobacillus salivarius* | Positive association (oral *Ligilactobacillus salivarius* supplementation associated with significantly higher live birth rate and improved pregnancy outcomes compared with placebo) |
| Kyono et al., 2019 [3] | Endometrial fluid (EF) aspirated using IUI catheter; collected once per IVF cycle (some patients had multiple cycles) | Placed in 1 mL MMB collection tube (DNA Genotek) and sent to Varinos Inc, Tokyo, Japan for microbiome analysis | NR | NR | NR | NR | NR | NR | NR | NR | t-test, Mann-Whitney U test, chi-square, or Fisher’s exact test; P < 0.05 considered significant | *Lactobacillus*-dominated microbiota (LDM) defined as endometrial microbiota dominated by *Lactobacillus* species (relative abundance-based classification); non-*Lactobacillus*-dominated microbiota (NLDM) defined by dominance of non-*Lactobacillus* taxa. | Positive association (LDM associated with higher clinical pregnancy rate and higher implantation success compared with NLDM, despite heterogeneity in miscarriage rates). |
| Di Pierro et al. 2023 [4] | Conegliano Hospital, Italy, 2020–2021 (Retrospective) | Vaginal-rectal swabs; endometrial therapy timing according to IVF cycle and swab results | NR | NR | NR | NR | NR | NR | NR | NR | Descriptive statistics; SAS categorical data analysis (JMP14.3); multiple logistic regression; CHAID segmentation model; Pearson/Fisher’s exact test; odds ratios; continuous variables: descriptive & multivariate analysis; nominal/ordinal: chi-square; p < 0.05 | *Lactobacillus* dominance not explicitly defined as sequencing-based threshold; inferred indirectly via restoration of vaginal eubiosis following probiotic administration (clinical microbiology-based categorization rather than OTU/ASV cut-off). | Positive association (oral *Lactobacillus* *crispatus* M247 supplementation associated with higher clinical pregnancy rate and higher live birth rate; strongest effect observed in blastocyst transfer subgroup). |
| Iwami et al. 2023 [5] | Endometrial biopsy (EB) between days 15–25 of natural cycle or days 5–7 of medicated cycle; vaginal swabs collected prior to EB | EB: RNAlater tubes, stored 4 °C for 4–72 h, shipped at room temp to Igenomix (Valencia, Spain) | QIAamp cador Pathogen Mini Kit (Qiagen) | 16S rRNA gene (full protocol per EMMA, V1–V9 coverage NR) | NR | Ion Torrent S5 XL Sequencer; reads/sample NR | NR | NR | NR | NR | Descriptive statistics; chi-square, unpaired t-test, Mann-Whitney U, Kruskal-Wallis, Fisher’s exact test with Bonferroni correction; p < 0.05 | *Lactobacillus*-dominated microbiota (LDM) defined as ≥90% *Lactobacillus* abundance in endometrial microbiota; non-LDM defined as ≤90% *Lactobacillus* or dominance of non-*Lactobacillus* taxa (EMMA classification: normal vs abnormal/mild dysbiosis/ultralow biomass). | Positive association (restoration of *Lactobacillus*-dominated endometrial microbiota associated with higher cumulative clinical pregnancy and ongoing pregnancy rates compared with control group receiving standard or no microbiota-directed treatment). |
| Bernabeu et al., 2019 [6] | Vaginal swabs taken during embryo transfer procedure, just before ET | Preserved at −80 °C until analysis | PureLink Microbiome DNA Purification Kit (ThermoFisher) | V3–V4 | OTUs clustered at 97% similarity | NR | Rarefaction: 1000 sequences/sample; alpha diversity indices (Shannon, Chao1) | Greengenes 99_otus, V3–V4 region | Positive and negative PCR controls included | NR | Descriptive statistics; Mann–Whitney U, Student t test; Shapiro–Wilk test; Pearson correlation; chi-squared test; p < 0.05 | *Lactobacillus* dominance defined as higher relative abundance of *Lactobacillus* spp. in vaginal microbiota (sequencing-based relative abundance pattern; no fixed cutoff such as ≥90% reported). | Positive association (*Lactobacillus*-dominated vaginal microbiota was significantly associated with higher clinical pregnancy rate; non-pregnant patients showed reduced *Lactobacillus* abundance and higher alpha diversity). |
| Hao et al., 2021 [7] | Cervical swabs collected immediately before embryo transfer | Stored at −80 °C until DNA extraction | PureLink Microbiome DNA Purification Kit (ThermoFisher) | V3–V4 | OTUs clustered at 97% similarity (UPARSE) | NR | Rarefaction and quality filtering: tags 220–500 bp, Q20, ≤3 ambiguous Ns, frequency >1 | RDP database v11.4, taxonomic assignment via RDP Classifier v2.12 | PCR positive and negative controls included | NR | Alpha diversity: Chao1, Shannon, Simpson; Beta diversity: unweighted UniFrac; Mann-Whitney U, Wilcox test, Kruskal-Wallis H, ANOVA, chi-square; LEfSe, Spearman correlation; logistic regression; p<0.05 | *Lactobacillus* dominance defined based on relative abundance comparisons between pregnancy and non-pregnancy groups using LEfSe and diversity analyses; no fixed cutoff threshold was applied (pattern-based definition). | Positive association (higher abundance of *Lactobacillus* spp. was significantly associated with clinical pregnancy in both fresh and frozen-thawed IVF cycles; *Lactobacillus* enrichment correlated with successful implantation outcomes). |
| Koort et al., 2023 [8] | Nova Vita Fertility Clinic / Tartu University, Estonia, 2010–2012 | Vaginal swabs from upper third posterior fornix collected on follicle puncture day; semen samples collected after 2–7 days abstinence | Vaginal swabs: one frozen for molecular analysis, the second for Gram-stained smear; semen: short incubation 37°C and liquefaction at room temp | QIAamp® DNA Blood Mini Kit (Qiagen) | V6 hypervariable region | NR | NR | NR | NR | NR | Microbial community profiling based on 16S rDNA sequencing on Illumina HiSeq2000; Nugent score for vaginal BV; Gram-stained smear; phylogenetic analysis using geodesic distances; detailed bioinformatics/statistics in Table S3 of the original paper | *Lactobacillus* dominance was defined implicitly through two approaches: (1) *Lactobacillus* *crispatus*-dominant or lactic-acid-bacteria-predominant vaginal microbiome identified by 16S rDNA sequencing; and (2) Nugent score–based classification of vaginal microbiota (0–3 normal *Lactobacillus*-dominant, ≥7 bacterial vaginosis-associated dysbiosis). | Positive association (*Lactobacillus* *crispatus*-dominant or lactic-acid-bacteria–predominant microbiota was associated with higher clinical pregnancy rate in ART patients, whereas BV-type dysbiosis was associated with lower pregnancy rate). |
| Faisal et al. 2023 [9] | Vaginal swabs collected on the day of oocyte retrieval (fresh ET cycles) | Transferred in microbiological transport media for culture | Not applicable – culture-based study | Not applicable | Not applicable | NR | NR | NR | NR | NR | Vaginal pH compared between pregnant and non-pregnant women (Shapiro-Wilk test for normality, unpaired t-test); ICSI outcomes compared using Fisher’s exact test; frequencies and percentages reported; SPSS 23 | *Lactobacillus* dominance was not defined using sequencing-based thresholds; instead, *Lactobacillus* spp. presence was determined by culture-based identification on Gram-stained smears and selective agar media, and compared qualitatively between pregnant and non-pregnant groups. | Positive association (higher presence of *Lactobacillus* spp. was observed in the pregnant group, while non-pregnant women showed higher abundance of *Enterobacter* spp.; *Lactobacillus* spp. presence was significantly associated with ICSI pregnancy outcome). |
| Guan et al., 2023 [10] | Cervical swabs collected before endometrium transformation in HRT-FET cycles | Stored at room temperature in DNA storage buffer (100 mM Tris–HCl pH9, 40 mM EDTA, 4 M guanidine thiocyanate, 0.001% bromothymol); sent to lab within 2 h | Qiagen Fecal DNA Extraction Kit (Qiagen, Hilden, Germany) | Full-length 16S rRNA | OTUs clustered at 99% similarity | NR | NR | SILVA_132_SSURef_Nr99 | NR | NR | α diversity: QIIME1 v1.8.0; group differences: Kruskal–Wallis sum-rank test; PCoA and Adonis in R; logistic regression for pregnancy outcomes; ROC curve via GraphPad Prism 6; SPSS 23 | *Lactobacillus* dominance was defined using community state types (CMTs) based on 16S rRNA full-length sequencing: CMT1 was dominated by *Lactobacillus* *crispatus* (*Lactobacillus*-dominant), CMT2 was dominated by *Lactobacillus* *iners*, and CMT3 represented a diverse/non-*Lactobacillus* bacterial community. | Positive association (CMT1 (*Lactobacillus* *crispatus*-dominated microbiota) was significantly associated with higher biochemical and clinical pregnancy rates compared with CMT2 and CMT3). |
| Pelzer et al., 2013 [11] | Follicular fluid (left & right ovary) and vaginal swabs; collected before oocyte retrieval | Follicular fluid stored at −80°C; vaginal swabs collected after vaginal prep with sterile water | Microbial culture: selective and differential media (Horse blood agar, anaerobic/aerobic incubation); semi-quantification by CFU/mL | NR | NR | NR | NR | NR | NR | NR | Continuous variables: Wilcoxon rank-sum or Kruskal-Wallis; proportions: Fisher’s exact test; multinomial logistic regression to associate microbial species with IVF outcomes; significance p<0.05 | *Lactobacillus* dominance was not explicitly defined; instead, presence of *Lactobacillus* spp. in follicular fluid was determined by culture-based microbiological identification (CFU/mL), and evaluated as a colonizing bacterial species compared with other detected genera. | Positive association (presence of *Lactobacillus* spp. in follicular fluid was associated with higher embryo transfer and pregnancy rates, whereas other bacterial species such as *Streptococcus* and *Bifidobacterium* were associated with poorer reproductive outcomes). |
| Haahr et al., 2016 [12] | Vaginal swabs from posterior fornix; collected at first consultation (2–4 weeks prior to IVF) | One swab stored at −80°C (Copan Eswab™) | FastDNA™ SPIN kit for Soil (MP Biomedicals) | qPCR for G. vaginalis, A. vaginae, L. crispatus, L. jensenii, L. gasseri, L. iners | NR | NR | ROC curve used to define abnormal vaginal microbiota | Nugent score as reference standard | NR | Yes; threshold determined by ROC curve using Nugent BV as gold standard | Mann-Whitney U-test, Kruskal-Wallis test, Fisher's exact test, chi-square, logistic regression (adjusted for confounders), ROC curve; inter-rater Kappa for Nugent score; significance p<0.05 | Normal vaginal microbiota was defined by qPCR profiles dominated by Lactobacillus spp. (L. crispatus, L. jensenii, L. gasseri, L. iners) with low concentrations of Gardnerella vaginalis and/or Atopobium vaginae; abnormal microbiota was defined by elevated G. vaginalis and/or A. vaginae based on ROC-derived thresholds. | Positive association (*Lactobacillus*-dominated/normal vaginal microbiota was associated with higher clinical pregnancy rates after IVF, whereas qPCR-defined abnormal microbiota was associated with markedly reduced clinical pregnancy rates (9%; P = 0.004)). |
| Moreno et al., 2016 [13] | Endometrial fluid (EF) and vaginal aspirates (VA); EF collected at LH+2 and LH+7, VA before EF aspiration | Frozen EF/VA | MagNa Pure Compact Nucleic Acid Isolation Kit I (Roche) | V3–V5 region of 16S rRNA | OTUs clustered at 97% sequence similarity using UCLUST | NR | Low-quality OTU filtering: sequences with ≤2 reads per genus or mean RDP score <0.9 removed | Ribosomal Database Project (RDP) Classifier v2.2 | NR | NR | Alpha diversity: Shannon & Simpson via QIIME; PCA & PCoA using Bray-Curtis; hierarchical clustering (R vegan); Student t-test, ANOVA, χ², Adonis, logistic regression, CART; supervised ML (ROCR, rpart) | *Lactobacillus*-dominated (LD) endometrial microbiota defined as ≥90% *Lactobacillus* OTUs in endometrial fluid; non-*Lactobacillus*-dominated (NLD) defined as <90% *Lactobacillus* OTUs with >10% other bacteria. | Positive association (*Lactobacillus*-dominated endometrial microbiota was associated with significantly higher implantation, clinical pregnancy, ongoing pregnancy, and live birth rates, whereas non-*Lactobacillus*-dominated microbiota was associated with poorer reproductive outcomes and higher miscarriage rates). |
| Moini et al., 2018 [14] | Vaginal samples obtained before oocyte retrieval | Vaginal swabs | NR | NR | Culture-independent scoring (Nugent) | Gram-stain based morphotypes (BV-associated bacteria) | NR | NR | NR | NR | Chi-square, ANOVA, relative risk, odds ratio; prevalence and outcome comparisons (implantation, pregnancy, miscarriage, preterm delivery, live birth) | Vaginal microbiota classified using Nugent score: Normal (0-3), Intermediate (4-6), and BV (7-10). *Lactobacillus* dominance was inferred from normal Nugent flora (low BV-associated bacteria and predominance of *Lactobacillus* morphotypes). | No significant association (BV/intermediate vaginal microbiota was not significantly associated with implantation, clinical pregnancy, early miscarriage, preterm delivery, or live birth outcomes in ICSI cycles for male-factor infertility). |
| Hashimoto & Kyono, 2019 [15] | Endometrial fluid collected during mock transfer before vitrified-warmed blastocyst transfer | Endometrial fluid | Stored in 1 mL MMB collection tube (DNA Genotek, Canada), sent to Varinos Inc., Japan | Genomic DNA extracted using Agencourt Genfind v2 Blood & Serum DNA Isolation Kit (Beckman Coulter, USA); treated with proteinase K and lysozyme | 16S rRNA gene amplicon sequencing, Illumina MiSeq platform, paired-end 2 × 200 or 2 × 300 bp | V4 hypervariable region of 16S rRNA | OTUs clustered at 97% identity using open-reference OTU picking; taxonomy assigned with RDP classifier against Greengenes 13_8 | ZymoBIOMICS microbial community standard used to assess primer performance; blank control (UltraPure DNase/RNase-free water) included | Background/blank-characteristic OTUs subtracted (9 taxa: Acinetobacter, Escherichia, Flavobacterium, Janthinobacterium, Methylobacterium, Pseudomonas, Rhodococcus, Sphingomonas, Stenotrophomonas); low-abundance taxa <0.01% filtered | QIIME 1.9.1 used for quality filtering, chimera checking, OTU clustering; rarefied to 5000 sequences/sample; EA-Utils fastq-join for merging; USEARCH for QC | t-test, Mann–Whitney U, chi-square, Fisher’s exact test; P < 0.05 considered significant | Endometrial eubiosis defined as ≥80% *Lactobacillus* + *Bifidobacterium* in endometrial fluid; dysbiosis defined as <80% with increased *Atopobium*, *Gardnerella*, and *Streptococcus*. | No significant association (clinical pregnancy and miscarriage rates did not differ significantly between eubiotic and dysbiotic endometrial microbiota groups in IVF patients undergoing vitrified-warmed blastocyst transfer). |
| Koedooder et al. 2019 [16] | Vaginal samples collected before fresh Day 3 ET | Vaginal swabs | NR | NR | Molecular IS-pro technique (amplicon length profiling of 16S–23S interspace regions) | 16S–23S interspace | OTU-like units defined by IS-pro fragment lengths; CST assignment based on dominant species (Ravel et al., 2011) | NR | NR | Vaginal microbiota profiles assigned to 5 CSTs; prediction model built using selected species identified by IS-pro | Multivariate model for IVF outcome; external validation in independent cohort; prediction accuracy assessed | Favorable vaginal microbiome characterized by predominance of *Lactobacillus* spp.; unfavorable profile defined by low *Lactobacillus* abundance (<20%), high *Lactobacillus* *jensenii* (>35%), presence of Gardnerella vaginalis IS-pro type 1, and/or high *Proteobacteria* abundance (>28%). | Positive association (*Lactobacillus*-dominated/favorable vaginal microbiota was associated with higher pregnancy likelihood after IVF/ICSI, whereas unfavorable microbiome profiles were associated with markedly reduced pregnancy rates, including a low-chance subgroup with ~6% pregnancy rate). |
| Vergaro et al. 2019 [17] | Vaginal sample on day of embryo transfer | Vaginal mucus | Stored at −20°C in sterile vials until processed | Whole gDNA extracted using QIAamp DNA Mini Kit (Qiagen); concentration measured by Nanodrop | Not culture-based | Not NGS; targeted qPCR for selected bacterial species | Not OTU/ASV-based; absolute quantification using standard curves from reference bacterial gDNA (DSMZ) | Negative controls included in qPCR; melting curve analysis to confirm specificity | Low-biomass samples quantified using human RPLP0 gene normalization | qPCR quantification used to generate relative abundance and absolute genome copies; vaginal microbiota profiles defined as Lactobacillus-dominated (≥90%) or BVB-dominated (>10%) | Univariate chi-square test for microbiota profile differences between live birth vs non-live birth, and pregnancy vs non-pregnancy; significance P<0.05 | *Lactobacillus*-dominated vaginal microbiota was defined by predominance of *Lactobacillus* spp. (particularly *Lactobacillus crispatus*, *Lactobacillus gasseri*, *Lactobacillus jensenii*, or *Lactobacillus iners*); abnormal vaginal microbiota (AVM) was defined by dominance of bacterial vaginosis-associated bacteria, including *Gardnerella vaginalis*, *Atopobium vaginae*, *Mycoplasma hominis*, and *Prevotella* spp. | Positive association (*Lactobacillus*-dominated vaginal microbiota, especially *Lactobacillus crispatus*-dominant profiles, was associated with significantly higher biochemical pregnancy, clinical pregnancy, and live birth rates, whereas BV-like/abnormal vaginal microbiota was not directly associated with reduced live birth rates overall). |
| Iwami et al., 2020 [18] | Endometrial tissue (day 15–25 of menstrual cycle) | Endometrial biopsy | NR | NR | Not culture-based | NGS for EMMA (likely 16S rRNA) | NR | NR | NR | NGS sequences analyzed for endometrial microbiome patterns; specific pipeline not reported | Multivariate generalized linear model for ongoing pregnancy; Kaplan-Meier survival analysis for time-to-pregnancy | *Lactobacillus*-dominated endometrial microbiota defined as ≥90% *Lactobacillus* spp. (LDM); non-LDM defined as <90% *Lactobacillus* spp. with increased presence of *Gardnerella* and other pathogenic taxa (EMMA-based classification). | Positive association (EMMA-guided restoration toward *Lactobacillus*-dominated microbiota was associated with improved reproductive performance, including comparable or improved ongoing pregnancy rates and significantly earlier achievement of pregnancy after intervention in women with recurrent implantation failure). |
| Riganelli et al., 2020 [19] | Vaginal fluid (day 21 of cycle) and endometrial biopsies (day 21, before ART stimulation) | Vaginal secretions & endometrial tissue | Endometrial biopsies stored in Allprotect Tissue Reagent (Qiagen) at −80°C; vaginal samples also stored at −80°C | DNeasy Blood and Tissue kit (Qiagen) with additional lysozyme step for Gram-positive bacteria; DNA quantified with Nanodrop; integrity checked by agarose gel | Not culture-based | 16S rRNA V3-V4 region | OTUs clustered at 97% similarity using Mothur v1.39.5; SILVA rDNA Database v.1.19; sample coverage >99% | Negative controls (blanks) and mock microbial DNA community standard (ZymoBIOMICS D6305) | Pipelle catheter protected by Artacath to avoid contamination from lower tract; rare OTUs <5 reads removed | Mothur for OTU assignment; SciKit-Bio v0.4.1 for α- and β-diversity (observed_OTUs, Shannon, Bray-Curtis, PCoA); PLS-DA and VIP; network analysis with Pearson correlations and Gephi v0.9.2; LEfSe for differential taxa | Mann-Whitney U and Kruskal-Wallis tests; Fisher’s test (Freeman-Halton); FDR correction at 10%; multivariate network analysis; significance P≤0.05 | *Lactobacillus*-dominated microbiota was defined based on the relative abundance of *Lactobacillus* spp. as the predominant taxa within the microbial community (with dominance inferred from OTU-based relative abundance profiles; no fixed percentage threshold explicitly stated in *this* study). | Positive association (*Lactobacillus*-dominated endometrial/vaginal microbiota was associated with higher implantation and live birth rates, while non-*Lactobacillus*-dominant microbiota was associated with implantation failure and lower live birth rates). |
| Kong et al., 2020 [20] | Vaginal swabs collected before endometrial preparation | Vaginal secretions | Added 20% glycerol, immediately frozen at −80°C | Genomic DNA extracted using Tiangen Biotech kit with bead-beating; concentration and quality measured with NanoDrop | Not culture-based | V4 region of 16S rRNA | OTUs clustered at ≥97% similarity using UCLUST; accession PRJNA554010 | NR | NR | QIIME v1.8.0 and USEARCH v5.2.236 for sequence processing; α-diversity: observed-OTUs, Chao1, Shannon, Simpson, ACE, goods-coverage; β-diversity: PCA, PCoA, NMDS; weighted UniFrac; PICRUSt for metabolic function prediction | SPSS 23.0, GraphPad Prism 8, Excel; Student’s t-test, one-way or two-way ANOVA; p<0.05 considered significant | *Lactobacillus*-dominated vaginal microbiota was defined based on relative abundance patterns derived from 16S rRNA sequencing, where higher proportion of *Lactobacillus* spp. constituted the dominant community state, without a fixed numerical cutoff explicitly stated in the *study*. | Positive association (*Lactobacillus*-enriched vaginal microbiota was associated with higher clinical pregnancy rates after IVF, whereas increased abundance of *Gardnerella*, *Atopobium*, and *Prevotella* was associated with lower pregnancy rates). |
| Diaz-Martínez et al., 2021 [21] | Vaginal swabs: (1) secretory phase previous to FET, (2) day of ET, (3) day of pregnancy test; Endometrial sample: secretory phase previous to FET | Vaginal swabs, endometrial samples | Stored at −80°C until analysis | PureLink Microbiome DNA Purification Kit (Thermo Fisher); quantified with Qubit 2.0 | Not culture-based | V3–V4 region of 16S rRNA | OTUs clustered at 97% similarity; taxonomic assignment using Greengenes 99_otus filtered to V3–V4 | Positive and negative controls included in PCR and library preparation | Rarefaction analysis for alpha diversity (1000 sequences/sample); sequences filtered and denoised with Deblur | QIIME2 for alpha & beta diversity; MicrobiomeAnalyst for further analysis; PCoA for beta diversity; PERMANOVA for group differences | Descriptive statistics (frequencies, mean±SD); Student’s t-test or Mann–Whitney U for continuous variables; chi-square for categorical variables; linear mixed-effects model for longitudinal vaginal microbiome and clinical outcomes; p<0.05 | *Lactobacillus* dominance was defined based on relative abundance of *Lactobacillus* spp. in the vaginal microbiota (high dominance observed when *Lactobacillus* constituted the vast majority of the community, ~>95% in pregnant group; no strict cutoff threshold explicitly defined in the methods). | Positive association (higher *Lactobacillus* abundance in vaginal samples at embryo transfer was associated with higher β-hCG positivity and clinical pregnancy rates, while increased Streptococcus and *Prevotella* abundance was associated with non-pregnancy; no significant association was observed in endometrial samples). |
| Usman et al., 2021 [22] | High vaginal swabs (HVS) and follicular fluid collected on the day of oocyte retrieval before IVF | Vaginal swabs, follicular fluid | Swabs and follicular fluid collected in sterile tubes → transported to microbiology lab within 15 min | Culture-based identification; gram staining for initial morphology; sub-culturing on selective media (MRS Lactobacillus agar, Yeast extract agar, Chromagar Orientation); physiobiochemical tests (motility, catalase, indole, urease, germ tube, oxidase, sugar fermentation) | Culture on Blood, Chocolate, MacConkey, Sabouraud agar; incubated 24–72 h under aerobic/CO2 conditions | NR | NR | Standard aseptic procedures; laminar hood during follicular fluid handling | Follicular fluid and swabs processed immediately; care to avoid contamination; differentiating colonizers vs. contaminants by comparing vaginal swab and follicular fluid results | NR | Continuous variables: mean ± SD or median (IQR); categorical: n (%); chi-square or Fisher exact for associations; logistic regression (univariable and multivariable) to evaluate follicular fluid colonization effect on fertilization and pregnancy; p<0.05 | Presence of *Lactobacillus* spp. among follicular fluid or vaginal isolates; not explicitly quantified as a dominance threshold (culture-based identification rather than sequencing-derived relative abundance cutoff). | No significant association (after adjustment, follicular fluid microbial colonization was not associated with fertilization rate or pregnancy outcomes; unadjusted differences in fertilization and pregnancy rates were not statistically significant). |
| Zeng et al., 2021 [23] | Vaginal and cervical canal swabs, collected on the day of embryo transfer before operation | Vaginal and cervical swabs | Swabs placed in 2 mL Falcon tubes → liquid nitrogen within 30 min → stored at −80°C | DNeasy PowerSoil kit (Qiagen); DNA concentration measured by NanoDrop2000 | 16S rRNA gene sequencing, Illumina NovaSeq 6000 | V3–V4 regions | OTUs clustered at 97% similarity using Vsearch; representative sequences assigned via Greengenes using RDP classifier | NR | Low-quality reads, ambiguous sequences, and reads <200 bp removed; chimeras removed using UCHIME | Trimmomatic for trimming, FLASH for merging reads, QIIME for quality control and OTU picking, α-diversity (Shannon–Wiener), β-diversity (weighted UniFrac, PCoA), PERMANOVA, Sorenson index, Metastat, LDA, RandomForest for genus importance | Continuous variables: mean±SD or median (IQR); categorical: n (%); normality: Kolmogorov–Smirnov; t-test/Mann–Whitney U for continuous, χ2 test for categorical; ROC curves for predictive genera; SPSS v25; p<0.05 | Based on Gram-stained vaginal smear morphology using the Nugent scoring system; *Lactobacillus*-dominant defined as Nugent score 0-3 (predominance of large Gram-positive rods), and non-*Lactobacillus*-dominant defined as Nugent score 4-10. | Positive association (*Lactobacillus*-dominant vaginal microbiota was associated with higher live birth rates, while non-*Lactobacillus* dominance was associated with significantly lower live birth rates, higher preclinical pregnancy loss, and higher preterm birth rates). |
| Wang et al. 2021 [24] | Vaginal and cervical canal swabs, collected on the day of embryo transfer before operation | Vaginal and cervical swabs | Swabs placed in 2 mL Falcon tubes → liquid nitrogen within 30 min → stored at −80°C | DNeasy PowerSoil kit (Qiagen); DNA concentration measured by NanoDrop2000 | 16S rRNA gene sequencing, Illumina NovaSeq 6000 | V3–V4 regions | OTUs clustered at 97% similarity using Vsearch; representative sequences assigned via Greengenes using RDP classifier | NR | Low-quality reads, ambiguous sequences, and reads <200 bp removed; chimeras removed using UCHIME | Trimmomatic for trimming, FLASH for merging reads, QIIME for quality control and OTU picking, α-diversity (Shannon–Wiener), β-diversity (weighted UniFrac, PCoA), PERMANOVA, Sorenson index, Metastat, LDA, RandomForest for genus importance | Continuous variables: mean±SD or median (IQR); categorical: n (%); normality: Kolmogorov–Smirnov; t-test/Mann–Whitney U for continuous, χ2 test for categorical; ROC curves for predictive genera; SPSS v25; p<0.05 | Not explicitly defined as a fixed threshold. *Lactobacillus* abundance was assessed at the genus/species level (16S rRNA sequencing), and associations were evaluated based on relative abundance patterns (particularly *Lactobacillus* iners), without a predefined cutoff for “dominance” (e.g., no ≥80% or ≥90% *Lactobacillus* criterion reported). | Negative association (higher abundance of specific vaginal *Lactobacillus* species, particularly *Lactobacillus* *iners*, along with other taxa, was associated with reduced likelihood of clinical pregnancy after IVF; overall microbial community structure was not significantly different between pregnant and non-pregnant groups). |
| Karaer et al. 2021 [25] | Vaginal posterior fornix swabs, collected on day of embryo transfer | Vaginal swab | Stored at −80°C until analysis | QiAamp DNA Microbiome Kit (Qiagen), DNA quantity & purity assessed by NanoDrop | 16S rRNA sequencing | V3–V4 hypervariable regions | NR | Laboratory blinded to ART outcomes | NR | Details in Supporting Information; likely OTU/ASV assignment and taxonomic classification standard for 16S V3–V4 | Sample size calculation via shinyMB; Kolmogorov–Smirnov test for normality; Student t-test / Mann-Whitney U-test for continuous variables; Pearson χ2 / Fisher exact for categorical; SPSS v11; significance P≤0.05 | Based on relative abundance from 16S rRNA gene sequencing of vaginal swabs at embryo transfer; *Lactobacillus* dominance was not defined using a fixed threshold (e.g., ≥80% or ≥90%), but inferred from overall proportional abundance of *Lactobacillus* spp. compared with other taxa (notably *Streptococcus* and *Gardnerella*). | Positive association (higher relative abundance of vaginal *Lactobacillus* spp. was observed in the pregnant group, while reduced *Lactobacillus* and increased *Streptococcus* and *Gardnerella* were associated with non-pregnancy; *Streptococcus* was identified as the strongest discriminator of IVF failure). |
| Kim et al. 2022 [26] | Vaginal swabs: posterior fornix, before oocyte retrieval; Follicular fluid: aspirated from largest follicles during oocyte retrieval | Vaginal swab, Follicular fluid | Swabs: Amies agar gel transport media; Follicular fluid: sterile test tubes → sterile Falcon tube | NR | Culture-based | Aerobic & anaerobic bacteria | NR | Sterile collection, irrigation of vaginal wall, sterile needles, condom-covered ultrasound probe | NR | NR | Chi-square / Fisher’s exact test for proportions; Mann-Whitney U test for continuous variables; SPSS v22 | Not defined in this study. Microbiological assessment was culture-based (aerobic/anaerobic culture of follicular fluid and vaginal swabs), identifying specific organisms rather than quantifying *Lactobacillus* abundance or using a dominance threshold (no *Lactobacillus*-dominant vs non-dominant classification reported). | Negative association (presence of microorganisms in follicular fluid was associated with lower clinical pregnancy rates, particularly for day 3 embryo transfer; microbial positivity in follicular fluid was linked to reduced IVF success compared with negative cultures). |
| Ji et al., 2022 [27] | Vaginal swabs collected from upper and middle 1/3 of vagina on the day of endometrial transformation (before FET) | Vaginal swab | Applied to sterile slides, heat-fixed and Gram-stained; no storage for sequencing (microscopy-based evaluation) | NR | Microscopy (Gram-stained smears) | Morphotypes scored by Nugent criteria | Morphotypes scored 0–10; categorized as normal, mild dysbiosis, severe dysbiosis | NR | NR | NR | One-way ANOVA, Welch’s test, Chi-square / Fisher exact test; SPSS v25 | *Lactobacillus* dominance was defined by the Nugent scoring system, where a score of 0-3 indicated *Lactobacillus*-dominant vaginal microbiota (predominant *Lactobacillus* morphotypes), 4-6 mild dysbiosis, and 7-10 severe dysbiosis with reduced Lactobacillus abundance. | Positive association (*Lactobacillus*-dominant vaginal microbiota was associated with higher implantation, clinical pregnancy, and ongoing pregnancy rates, whereas increasing dysbiosis was associated with poorer reproductive outcomes). |
| Villani et al., 2022 [28] | Immediately before oocyte pickup, prior to cervical disinfection | Cervical swab | DNA stabilisation buffer; centrifugation; −80 °C | QIAamp DNA Blood & Tissue Kit | Illumina MiSeq (2 × 300 bp) | 16S rRNA V3–V4 | OTU-based; GAIA v2.0; NCBI-based custom database | Quality controls via FastQC and BBDuk; no explicit blank controls reported | NR | GAIA v2.0; FastQC; BBDuk; BWA-MEM mapping | t-test; χ²/Fisher; PELORA penalized logistic regression; bootstrap resampling | *Lactobacillus* dominance was defined based on 16S rRNA gene sequencing of cervical swabs, where relative abundance of *Lactobacillus* spp. (including key species such as *Lactobacillus crispatus* and *Lactobacillus iners*) was used to characterize the cervical microbiota profile. Higher dominance of *Lactobacillus* was considered a *Lactobacillus*-dominant state, while reduced Lactobacillus abundance with increased anaerobic taxa (e.g., *Atopobium vaginae*) was considered non-dominant. | Positive association (higher abundance of *Lactobacillus* spp. was associated with favorable pregnancy outcomes, whereas reduced *Lactobacillus* dominance and increased anaerobic bacteria such as *Atopobium vaginae* were associated with unfavorable pregnancy outcomes). |
| Moreno et al., 2022 [29] | HRT cycle after 120 h progesterone; prior to frozen blastocyst transfer; ERA diagnostic cycle | Endometrial fluid (EF) and endometrial biopsy (EB) | RNAlater; 4 °C for 4 h → shipped RT → −80 °C | EF: enzymatic pre-digestion + QIAamp DNA Blood Mini Kit; EB: proteinase K + mechanical lysis + QIAsymphony tissue protocol | Ion S5 XL (Ion 530 Chip) | 7 hypervariable regions (V2–4–8, V3–6, V7–9) | OTU-based; RDP classifier v2.2; Greengenes v13.8 | Blank preservation buffer controls; PCR negative (water) and positive (E. coli DNA) controls per run; decontam (prevalence-based) | Biomass detectability threshold (filtered/mapped reads ratio: >0.65 EF; >0.7 EB); removal of kitome taxa and known contaminants | QIIME 2; RDP; decontam; clr transformation; taxa prevalence and abundance filtering | ANOVA; Mann–Whitney U; χ²/Fisher; multinomial logistic regression; Bayesian inference (PyMC3) | *Lactobacillus* dominance was defined as an endometrial microbiota profile in which *Lactobacillus* spp. constituted the predominant taxa based on 16S rRNA gene sequencing (relative abundance-based assessment after quality filtering and log-ratio transformation), with higher *Lactobacillus* Z-scores indicating dominance compared with other bacterial taxa. | Positive association (higher *Lactobacillus* abundance associated with increased live birth and clinical pregnancy rates, while dysbiotic microbiota enriched in non-*Lactobacillus* taxa was associated with reduced reproductive outcomes and higher miscarriage/no pregnancy rates). |
| Ge et al., 2023 [30] | Vaginal secretions collected 3 days before ART (IUI / IVF-ET / ICSI); posterior vaginal fornix; pregnancy outcome (clinical pregnancy) followed | Mixed with sterile saline; analyzed immediately or stored at −20 °C ≤3 days | Multiplex quantitative fluorescent PCR | 22 predefined vaginal microorganisms (Lactobacillus spp., BV-associated bacteria, Candida, GBS, STIs, HSV) | NR | NR | Positive defined as Cq ≤38; retesting for Cq 38–40; detection limit 5.0×10² copies/mL; intra-/inter-assay CV ≤5%; no reported cross-reactivity | Manufacturer-defined pathogen panel | Kit internal controls; external negative controls not reported | Analytical validation provided by manufacturer (100% concordance for reference materials) | χ² test for categorical variables; SPSS v22.0; P ≤0.05 | *Lactobacillus* dominance was defined as a vaginal microbiota profile in which *Lactobacillus* spp. accounted for ≥95% of total detected microorganisms by multiplex quantitative fluorescent PCR, with absence of detected pathogenic microorganisms. | Positive association (*Lactobacillus*-dominant vaginal microecology was associated with higher clinical pregnancy rates across ART modalities, whereas non-dominant/dysbiotic microbiota was associated with lower clinical pregnancy rates). |
| Tong et al., 2023 [31] | Vaginal swabs collected longitudinally during pregnancy (1st: 10–13⁺⁶ w, 2nd: 20–27⁺⁶ w, 3rd: 28–33⁺⁶ w); IVF vs natural conception; term vs preterm delivery | Sterile tubes; stored at −80 °C until DNA extraction | CTAB-based extraction (in-house protocol) | V3–V4 | OTUs (97% similarity) | NR | FLASH merging; QIIME v1.7.0 quality filtering; chimera removal with UCHIME (Gold database); OTU clustering with UPARSE; no rarefaction threshold specified | SILVA database with RDP classifier v2.2 | NR | NR | Alpha diversity (Observed species, Chao1, Shannon, Simpson, ACE, Good’s coverage); beta diversity via QIIME; Student’s t-test for group comparisons; R v2.15.3 | *Lactobacillus* dominance was defined as a vaginal microbial community in which *Lactobacillus* spp. constituted the dominant genus based on 16S rRNA gene sequencing (OTU-based taxonomic assignment with ≥97% similarity), as determined from relative abundance profiles and taxonomic annotation. | Positive association (higher *Lactobacillus*-dominant vaginal microbiota and favorable microbial community structure were associated with higher likelihood of term delivery and better pregnancy outcomes, whereas reduced *Lactobacillus* dominance and altered microbial diversity were associated with increased risk of preterm birth). |
| Miyagi et al., 2023 [32] | Vaginal and endometrial secretions collected simultaneously on cycle days 8–10 prior to frozen embryo transfer | Not explicitly reported; samples processed for DNA extraction; contamination screening performed | Previously reported protocol (Kyono et al., 2018b; Mariya et al., 2021); commercial kit not specified | V1–V2 | OTUs | NR | Quality filtering of paired-end reads; OTU clustering; removal of taxa frequently detected in negative controls; exclusion of samples clustering with negative controls based on weighted UniFrac | NR | UltraPure™ DNase/RNase-Free water; samples clustering with negative controls excluded | NR | Weighted UniFrac, Bray–Curtis, PCoA, PERMANOVA; ROC curve–derived cutoffs for Lactobacillus and pathological bacteria; t-test, Welch’s t-test, Wilcoxon rank-sum, Fisher’s exact test; R v3.6.2 | *Lactobacillus* dominance was defined as a vaginal or endometrial microbiome composition classified by 16S rRNA gene sequencing in which *Lactobacillus*-dominated community state types (CST I-III per Ravel et al., 2011) and/or high *Lactobacillus* spp. abundance (above study-defined threshold) were present, particularly when accompanied by low abundance of pathological taxa such as *Gardnerella* spp. and *Prevotella* spp. | Positive association (higher *Lactobacillus*-dominant and low-pathogenic bacterial microbiota in both vaginal and endometrial compartments was significantly associated with increased pregnancy rates after embryo transfer, while dysbiotic profiles enriched in Gardnerella/Prevotella spp. were associated with lower pregnancy outcomes). |
| Cariati et al., 2023 [33] | Endometrial fluid aspirated via double-lumen transfer catheter at embryo transfer | Aspirated fluid added to 8 mL BHI medium; transported to lab within 10–15 min; immediate culture | NA (culture-based study; no DNA extraction for sequencing) | NR | NR | NR | NR | MALDI-TOF MS reference spectra database (Bruker Daltonics) | NR | NR | Categorical variables as frequency (%); χ² or Fisher’s exact test; R v4.0.3; α = 0.05 | *Lactobacillus* dominance was defined as the presence and predominance of *Lactobacillus* spp. detected by culture (culturomics), without a quantitative cutoff threshold; samples in which *Lactobacillus* was the main isolated genus were considered *Lactobacillus*-dominant. | Positive association (clinical pregnancy); higher frequency of *Lactobacillus* spp. in pregnant women, while non-pregnancy was associated with higher abundance of *Enterobacteriaceae* and other non-*Lactobacillus* taxa. |
| Kadogami et al., 2023 [34] | Endometrial tissue collected during implantation window | OMNIgene-VAGINAL tube with stabilizing solution; mixed immediately; storage temperature not explicitly stated | MagNA Pure 24 Pathogen 1000 hp 3.1 protocol (Roche); pretreatment with MagNAPure Bacteria Lysis Buffer, Proteinase K and Lysozyme | V1–V2 region; primers 27Fmod / 338R | OTUs (QIIME 1.9.1; de novo uclust, 99.5% similarity) | Not reported; Illumina MiSeq paired-end | Adapter trimming (Trimmomatic); read joining (fastq-join); quality filtering (Q <25, length <250 bp or ≥400 bp removed); chimera/low-identity sequences excluded | Species-level assignment using custom database based on SILVA132 + STIRRUPS; BLAST alignment ≥99% identity and ≥95% coverage | ZymoBIOMICS mock community; DNase/RNase-free water blanks | NR | LD vs NLD classification (Lactobacillus ≥80%); continuous variables by t-test or Mann–Whitney; categorical variables by χ² or McNemar test; ANOVA with Tukey–Kramer for multiple groups; EZR software; p <0.05 | *Lactobacillus* dominance (LD) was defined as *Lactobacillus* spp. comprising ≥80% of the uterine microbiome based on 16S rRNA gene sequencing, while non-*Lactobacillus* dominance (NLD) was defined as *Lactobacillus* spp. comprising <80% of the total microbial community. | Positive association (implantation outcome varies by species); *Lactobacillus* *iners* dominance showed a negative association with implantation rate (lower implantation compared with other *Lactobacillus*-dominant profiles), whereas other *Lactobacillus*-dominant states were comparatively more favorable. |
| Väinämö et al., 2023 [35] | Vaginal swabs collected at fresh embryo transfer; additional swabs at 8th gestational week in pregnant women | Swabs frozen at −20 °C immediately; transferred to −80 °C within 4 weeks | Bead-beating–based DNA extraction (custom protocol, as previously described) | 16S rRNA V3–V4 region; primers 341F / 785R | ASVs via DADA2 | Not explicitly reported; Illumina MiSeq paired-end | Standard DADA2 quality filtering; ASV inference; no rarefaction reported | Species-level annotation via NCBI BLAST (taxminer workflow); CST classification using VALENCIA | NR | NR | Univariate comparisons with t-test or Mann–Whitney U; χ² or Fisher’s exact test for categorical variables; differential abundance via GroupTest (negative binomial GLM, mare R package) with FDR correction; presence >5%, dominance >50%, prevalence >1%; richness (specnumber), alpha diversity (Simpson), PERMANOVA (adonis2); analyses in SPSS 27 and R 4.2.2 | *Lactobacillus* dominance was defined as a relative abundance of *Lactobacillus* spp. >50% in the vaginal microbiota, based on 16S rRNA gene sequencing data and CST classification (VALENCIA tool). Additional operational categories included presence (>5%) and prevalence (>1% across samples). | Positive association (clinical pregnancy and live birth); higher *Lactobacillus* *crispatus* abundance and dominance were associated with clinical pregnancy, while *Lactobacillus* *iners* and BV-associated taxa were associated with non-pregnancy. |
| Bui et al., 2023 [36] | Endometrial tissue collected in mid-luteal phase of natural cycle prior to second IVF/ICSI cycle (5–8 days after LH surge) | Snap-frozen in liquid nitrogen within 3 min after biopsy; stored at −80°C | Bead-beating + chemical lysis (custom protocol) | 16S rRNA V1–V2 region | ASVs via DADA2 | NR | Samples with <100 bacterial reads discarded; human, mitochondrial, and unassigned reads removed | SILVA 138 | Positive (mock community) and negative (blank) controls included | NR | Wilcoxon rank sum for continuous data; Fisher’s exact test for categorical; alpha diversity: inverse Simpson & Chao1; beta diversity: PCA on clr-transformed data; ANCOM-BC for differential abundance with FDR correction; targeted bacterial group comparisons with Wilcoxon rank sum; no multivariable modeling due to small sample size; analyses in R 4.1.0 (phyloseq, microbiome) and SPSS 26.0 | *Lactobacillus* dominance was operationally defined by categorizing relative abundance of total *Lactobacillus* spp. in the endometrial microbiota as ≥90% (high dominance), 11-89% (intermediate), and ≤10% (low), with additional subgroup classification for *Lactobacillus* *crispatus*, *Lactobacillus* *iners*, and other lactobacilli. | Positive association (live birth); higher *Lactobacillus* crispatus relative abundance was associated with live birth, whereas overall α- and β-diversity were not significantly associated with pregnancy outcomes. |
| Nishio et al., 2024 [37] | Cervicovaginal mucus collected twice per patient: at oocyte retrieval and at embryo transfer | Immediately stored at −20°C, then transferred to −80°C deep freezer | ChargeSwitch Forensic DNA Purification Kit (Thermo Fisher) | 16S rRNA V3/V4 region, PCR with Bakt_341F/Bakt_805R primers | ASVs / OTUs via DADA2 | Not explicitly stated; paired-end 2×300 bp MiSeq | Reads <150 bp discarded; primers trimmed; quality score ≥20; merged reads 420 bp ± 10 bp overlap | Greengenes 13_8 clustered at 97%; SpeciateIT custom Lactobacillus database for species-level discrimination | NR | NR | Mann–Whitney U test for pregnant vs nonpregnant; Wilcoxon signed-rank for paired samples; Spearman correlation for bacteria-metabolite associations; volcano plots and pathway analysis using MetaboAnalyst 5.0; alpha diversity (observed OTUs/Sobs) via QIIME2 | Not explicitly defined as a categorical “dominance” threshold in the study; *Lactobacillus* was assessed as relative abundance (genus-level proportion based on 16S rRNA sequencing), analyzed mainly as a continuous variable. | No significant association between *Lactobacillus* abundance and clinical pregnancy outcome (pregnant vs non-pregnant groups showed no significant difference in *Lactobacillus* levels). |
| Wu et al., 2025 [38] | Cervical mucus collected on FET day; swab rotated in cervical canal after wiping secretions | Immediately immersed in liquid nitrogen; transported to lab | Qiagen DNA extraction kit | Full-length 16S rDNA (16S-FLAST) | OTUs clustered at 99% similarity via vSearch in QIIME2 | 2,014,560 unique full-length 16S sequences from 136 samples (>1,200 bp) | Low-quality sequences (<1,000 bp) excluded | SILVA_132_SSURef_Nr99 via Mothur v1.42.0 | NR | qPCR performed for cervical microbiota tracing | Categorical variables: χ² or Fisher’s exact; Continuous variables: t-test; Mann–Whitney U for relative abundance; alpha/beta diversity: vegan; Random forest model (R, randomForest) for NP vs CP; Multivariate binary logistic regression (SPSS 26) with backward stepwise; ROC curves: pROC; Visualization: ggplot2 | Not explicitly defined as a categorical “dominance” threshold in this study. *Lactobacillus* was analyzed mainly as a relative abundance variable (genus-level from 16S rDNA full-length sequencing), without a stated cutoff (e.g., ≥80% or ≥90%) to define dominance. | No significant association between *Lactobacillus* abundance and clinical pregnancy outcome. *Lactobacillus* levels were reported as similar between the clinical pregnancy and non-pregnancy groups, indicating no clear predictive or protective effect in this cohort. |
| Bar et al., 2025 [39] | Vaginal swabs collected at multiple timepoints: stimulation day 3, egg retrieval, embryo transfer (fresh and frozen cycles) | Swabs stored at -80°C until processing; eluted in sterile saline prior to DNA extraction | QIAamp 96 DNA QIAcube HT Kit (Qiagen); plate-based with bead beating and phenol-chloroform | V4 region of 16S rRNA; primers 515F/806R | Amplicon Sequence Variants (ASVs) via DADA2 | Not explicitly stated; typical MiSeq 300-cycle | Quality filtering, denoising, chimera removal using DADA2 | GTDB, genus-level aggregation | NR | NR | Alpha diversity (Shannon index, vegan), mixed-effects linear regression for repeated measures; associations with outcomes; machine learning: SVM with linear kernel, SMOTE for class imbalance, LOO-CV, F1-score, accuracy, SHAP for feature importance; R and Python (scikit-learn, imblearn, pandas, matplotlib) | Not defined by a fixed abundance cutoff (e.g., ≥80% or ≥90%). Instead, *Lactobacillus* was evaluated based on (1) genus-level relative abundance and (2) Community State Type (CST) classification using VALENCIA, where CST I (*Lactobacillus crispatus*-dominant), CST II (*Lactobacillus gasseri*-dominant), CST III (*Lactobacillus iners*-dominant), and CST V (*Lactobacillus jensenii*-dominant) represent *Lactobacillus*-dominant states, whereas CST IV represents non–*Lactobacillus*-dominant microbiota. | Positive association (*Lactobacillus*-dominant CSTs, especially CST I: *Lactobacillus* *crispatus*–dominant, associated with improved reproductive outcomes; non–*Lactobacillus*-dominant CST IV associated with poorer outcomes). |
| Alley et al. 2025 [40] | Vaginal swabs collected at time of FET, prior to embryo transfer | Swabs placed on ice, frozen at -20°C within 1 h, then transferred to -80°C | DNeasy PowerSoil Kit (Qiagen) | V4 region of 16S rRNA; primers 515F/806R | Amplicon Sequence Variants (ASVs) via DADA2 | Rarefied to 7996 reads per sample | Quality filtering, denoising, chimera removal using DADA2 | RDP classifier trained on GTDB release 202 | Extraction and PCR negative controls included | NR | Alpha diversity (Shannon, Simpson), beta diversity (PERMANOVA/adonis, vegan), DESeq2 for differential abundance, t-test, Fisher’s exact, Chi-square; Benjamini–Hochberg correction for multiple comparisons, significance p<0.05 | Not defined by a fixed abundance cutoff (e.g., ≥80% or ≥90%). *Lactobacillus* dominance was assessed as relative abundance derived from 16S rRNA ASV profiling, and interpreted at the community level (i.e., higher *Lactobacillus* proportion and *Lactobacillus*-dominant community structure vs. non-dominant microbiota). | Positive association (greater *Lactobacillus* dominance in vaginal microbiota was observed in the pregnant group, while non-pregnancy was associated with higher abundance of *Enterobacteriaceae* and other opportunistic bacteria). |

**Abbreviations:** ART, assisted reproductive technology; ASV, amplicon sequence variant; ANOVA, analysis of variance; AFC, antral follicle count; AVM, abnormal vaginal microbiota; BV, bacterial vaginosis; CMT, community state type; CFU, colony-forming unit; CST, community state type; DADA2, Divisive Amplicon Denoising Algorithm 2; DOR, diminished ovarian reserve; EB, endometrial biopsy; EF, endometrial fluid; EMMA, Endometrial Microbiome Metagenomic Analysis; ET, embryo transfer; FDR, false discovery rate; FET, frozen embryo transfer; FSH, follicle-stimulating hormone; GTDB, Genome Taxonomy Database; hCG, human chorionic gonadotropin; HRT, hormone replacement therapy; ICSI, intracytoplasmic sperm injection; IVF, in vitro fertilization; LDM, Lactobacillus-dominated microbiota; LH, luteinizing hormone; LEfSe, linear discriminant analysis effect size; NLDM, non–Lactobacillus-dominated microbiota; NGS, next-generation sequencing; NMDS, non-metric multidimensional scaling; NR, not reported; OCP, oral contraceptive pill; OTU, operational taxonomic unit; PCoA, principal coordinates analysis; PCR, polymerase chain reaction; PERMANOVA, permutational multivariate analysis of variance; POI, premature ovarian insufficiency; PRP, platelet-rich plasma; PROMIS, Patient-Reported Outcomes Measurement Information System; qPCR, quantitative polymerase chain reaction; RDP, Ribosomal Database Project; ROC, receiver operating characteristic curve; SILVA, ribosomal RNA gene database; SCFA, short-chain fatty acids; SPSS, Statistical Package for the Social Sciences; VA, vaginal aspirate.

**References**

1. Gilboa Y, Bar-Hava, I, Fisch, B, Ashkenazi, J, Voliovitch, I, Borkowski, T, Orvieto R. Does intravaginal probiotic supplementation increase the pregnancy rate in IVF-embryo transfer cycles? Reproductive biomedicine online. 2005;11:71‐75. https://doi.org/10.1016/s1472-6483(10)61301-6.

2. Huerga López C, Sánchez Martín, MJ, Herráez Moreta, A, Calvo Urrutia, M, Cristóbal García, I, Díaz Morillo, C, Blanco-Rojo, R, Sáez, ME, Olivares, M, Arroyo, R, Herranz, C, Alba, C, Rodríguez, JM, Fernández L. Ligilactobacillus salivarius CECT5713 Increases Term Pregnancies in Women with Infertility of Unknown Origin: a Randomized, Triple-Blind, Placebo-Controlled Trial. Nutrients. 2025;17. https://doi.org/10.3390/nu17111860.

3. Kyono K, Hashimoto T, Kikuchi S, Nagai Y, Sakuraba Y. A pilot study and case reports on endometrial microbiota and pregnancy outcome: An analysis using 16S rRNA gene sequencing among IVF patients, and trial therapeutic intervention for dysbiotic endometrium. Reprod Med Biol. 2019;18:72–82. https://doi.org/10.1002/rmb2.12250.

4. Di Pierro F, Sinatra F, Cester M, Da Ros L, Pistolato M, Da Parè V, et al. Effect of L. crispatus M247 Administration on Pregnancy Outcomes in Women Undergoing IVF: A Controlled, Retrospective, Observational, and Open-Label Study. MICROORGANISMS. 2023;11. https://doi.org/10.3390/microorganisms11112796.

5. Iwami N, Kawamata M, Ozawa N, Yamamoto T, Watanabe E, Mizuuchi M, et al. Therapeutic intervention based on gene sequencing analysis of microbial 16S ribosomal RNA of the intrauterine microbiome improves pregnancy outcomes in IVF patients: a prospective cohort study. J Assist Reprod Genet. 2023;40:125–35. https://doi.org/10.1007/s10815-022-02688-6.

6. Bernabeu A, Lledo B, Díaz MC, Lozano FM, Ruiz V, Fuentes A, et al. Effect of the vaginal microbiome on the pregnancy rate in women receiving assisted reproductive treatment. J Assist Reprod Genet. 2019;36:2111–9. https://doi.org/10.1007/s10815-019-01564-0.

7. Hao X, Li P, Wu S, Tan J. Association of the Cervical Microbiota With Pregnancy Outcome in a Subfertile Population Undergoing In Vitro Fertilization: A Case-Control Study. Front Cell Infect Microbiol. 2021;11. https://doi.org/10.3389/fcimb.2021.654202.

8. Koort K, Sõsa K, Türk S, Lapp E, Talving E, Karits P, et al. Lactobacillus crispatus-dominated vaginal microbiome and Acinetobacter-dominated seminal microbiome support beneficial ART outcome. Acta Obstet Gynecol Scand. 2023;102:921–34. https://doi.org/10.1111/aogs.14598.

9. Farooq Faisal S, Adnan Abdul Hameed W, Alwasiti E. The Influence of Vaginal Dysbiosis on Intracytoplasmic Sperm Injection Outcome. Arch Razi Inst. 2023;78:227–32. https://doi.org/10.22092/ARI.2022.358610.2260.

10. Guan W, Dong S, Wang Z, Jiao J, Wang X. Impact of a Lactobacillus dominant cervical microbiome, based on 16S-FAST profiling, on the reproductive outcomes of IVF patients. Front Cell Infect Microbiol. 2023;13:1059339. https://doi.org/10.3389/fcimb.2023.1059339.

11. Pelzer ES, Allan JA, Waterhouse MA, Ross T, Beagley KW, Knox CL. Microorganisms within human follicular fluid: effects on IVF. PLoS One. 2013;8:e59062. https://doi.org/10.1371/journal.pone.0059062.

12. Haahr T, Jensen JS, Thomsen L, Duus L, Rygaard K, Humaidan P. Abnormal vaginal microbiota may be associated with poor reproductive outcomes: a prospective study in IVF patients. Hum Reprod. 2016;31:795–803. https://doi.org/10.1093/humrep/dew026.

13. Moreno I, Codoñer FM, Vilella F, Valbuena D, Martinez-Blanch JF, Jimenez-Almazán J, et al. Evidence that the endometrial microbiota has an effect on implantation success or failure. Am J Obstet Gynecol. 2016;215:684–703. https://doi.org/10.1016/j.ajog.2016.09.075.

14. Moini A, Mohammadi Yeganeh L, Shiva M, Ahmadieh M, Salman Yazdi R, Hasani F, et al. Bacterial vaginosis and the risk of early miscarriage in women undergoing intracytoplasmic sperm injection cycles: a prospective cohort study. Hum Fertil (Camb). 2018;21:263–8. https://doi.org/10.1080/14647273.2017.1353709.

15. Hashimoto T, Kyono K. Does dysbiotic endometrium affect blastocyst implantation in IVF patients? J Assist Reprod Genet. 2019;36:2471–9. https://doi.org/10.1007/s10815-019-01630-7.

16. Koedooder R, Singer M, Schoenmakers S, Savelkoul PHM, Morré SA, de Jonge JD, et al. The vaginal microbiome as a predictor for outcome of in vitro fertilization with or without intracytoplasmic sperm injection: a prospective study. Hum Reprod. 2019;34:1042–54. https://doi.org/10.1093/humrep/dez065.

17. Vergaro P, Tiscornia G, Barragán M, García D, Rodriguez A, Santaló J, et al. Vaginal microbiota profile at the time of embryo transfer does not affect live birth rate in IVF cycles with donated oocytes. Reprod Biomed Online. 2019;38:883–91. https://doi.org/10.1016/j.rbmo.2018.12.019.

18. Iwami N, Kawamata, M, Ozawa, N, Yamamoto, T, Watanabe, E, Mizuuchi, M, Moriwaka, O, Kamiya H. Efficacy of endometrial microbiome metagenomic analysis and analysis of infectious chronic endometritis on in vitro fertilization outcome in women with recurrent implantation failure. Human reproduction (Oxford, England). 2020;35 SUPPL 1:i31.

19. Riganelli L, Iebba V, Piccioni M, Illuminati I, Bonfiglio G, Neroni B, et al. Structural Variations of Vaginal and Endometrial Microbiota: Hints on Female Infertility. FRONTIERS IN CELLULAR AND INFECTION MICROBIOLOGY. 2020;10. https://doi.org/10.3389/fcimb.2020.00350.

20. Kong Y, Liu Z, Shang Q, Gao Y, Li X, Zheng C, et al. The Disordered Vaginal Microbiota Is a Potential Indicator for a Higher Failure of in vitro Fertilization. Front Med (Lausanne). 2020;7:217. https://doi.org/10.3389/fmed.2020.00217.

21. Diaz-Martínez MDC, Bernabeu A, Lledó B, Carratalá-Munuera C, Quesada JA, Lozano FM, et al. Impact of the Vaginal and Endometrial Microbiome Pattern on Assisted Reproduction Outcomes. J Clin Med. 2021;10. https://doi.org/10.3390/jcm10184063.

22. Usman SF, Shuaibu IR, Durojaiye K, Medugu N, Iregbu KC. The presence of microorganisms in follicular fluid and its effect on the outcome of in vitro fertilization-embryo transfer (IVF-ET) treatment cycles. PLoS One. 2021;16:e0246644. https://doi.org/10.1371/journal.pone.0246644.

23. Zeng H, He D, Hu L, Abdullah RK, Zhang L, Jiang B, et al. Non-Lactobacillus dominance of the vagina is associated with reduced live birth rate following IVF/ICSI: a propensity score-matched cohort study. Arch Gynecol Obstet. 2022;305:519–28. https://doi.org/10.1007/s00404-021-06171-y.

24. Wang R, Zhou G, Wu L, Huang X, Li Y, Luo B, et al. The Microbial Composition of Lower Genital Tract May Affect the Outcome of in vitro Fertilization-Embryo Transfer. Front Microbiol. 2021;12:729744. https://doi.org/10.3389/fmicb.2021.729744.

25. Karaer A, Doğan B, Günal S, Tuncay G, Arda Düz S, Ünver T, et al. The vaginal microbiota composition of women undergoing assisted reproduction: a prospective cohort study. BJOG. 2021;128:2101–9. https://doi.org/10.1111/1471-0528.16782.

26. Kim SM, Won KH, Hong YH, Kim SK, Lee JR, Jee BC, et al. Microbiology of Human Follicular Fluid and the Vagina and Its Impact on in Vitro Fertilization Outcomes. Yonsei Med J. 2022;63:941–7. https://doi.org/10.3349/ymj.2022.0190.

27. Ji L, Peng C, Bao X. Effect of vaginal flora on clinical outcome of frozen embryo transfer. Front Cell Infect Microbiol. 2022;12:987292. https://doi.org/10.3389/fcimb.2022.987292.

28. Villani A, Fontana A, Barone S, de Stefani S, Primiterra M, Copetti M, et al. Identifying Predictive Bacterial Markers from Cervical Swab Microbiota on Pregnancy Outcome in Woman Undergoing Assisted Reproductive Technologies. J Clin Med. 2022;11. https://doi.org/10.3390/jcm11030680.

29. Moreno I., Garcia-Grau I., Perez-Villaroya D., Gonzalez-Monfort M., Bahceci M., Barrionuevo M.J., et al. Endometrial microbiota composition is associated with reproductive outcome in infertile patients. Microbiome. 2022;10:1. https://doi.org/10.1186/s40168-021-01184-w.

30. Ge Y-M, Lu J-C, Xu Y-H, Tang S-S, Zhi S-S, Liang Y-J. Correlations of joint detection of 22 vaginal microbes with routine examination results of vaginal secretions and assisted reproductive outcomes. Diagn Microbiol Infect Dis. 2023;106:115940. https://doi.org/10.1016/j.diagmicrobio.2023.115940.

31. Tong Y, Sun Q, Shao X, Wang Z. Effect of vaginal microbiota on pregnancy outcomes of women from Northern China who conceived after IVF. Front Endocrinol (Lausanne). 2023;14:1200002. https://doi.org/10.3389/fendo.2023.1200002.

32. Miyagi M, Mekaru K, Tanaka SE, Arai W, Ashikawa K, Sakuraba Y, et al. Endometrial and vaginal microbiomes influence assisted reproductive technology outcomes. JBRA Assist Reprod. 2023;27:267–81. https://doi.org/10.5935/1518-0557.20220040.

33. Cariati F, Carotenuto C, Bagnulo F, Pacella D, Marrone V, Paolillo R, et al. Endometrial microbiota profile in in-vitro fertilization (IVF) patients by culturomics-based analysis. Front Endocrinol (Lausanne). 2023;14:1204729. https://doi.org/10.3389/fendo.2023.1204729.

34. Kadogami D, Kimura F, Hanada T, Tsuji S, Nakaoka Y, Murakami T, et al. Impact of Lactobacillus in the uterine microbiota on in vitro fertilization outcomes. J Reprod Immunol. 2023;160:104138. https://doi.org/10.1016/j.jri.2023.104138.

35. Väinämö S, Saqib S, Kalliala I, Kervinen K, Luiro K, Niinimäki M, et al. Longitudinal analysis of vaginal microbiota during IVF fresh embryo transfer and in early pregnancy. Microbiol Spectr. 2023;11:e0165023. https://doi.org/10.1128/spectrum.01650-23.

36. Bui BN, van Hoogenhuijze N, Viveen M, Mol F, Teklenburg G, de Bruin J-P, et al. The endometrial microbiota of women with or without a live birth within 12 months after a first failed IVF/ICSI cycle. Sci Rep. 2023;13:3444. https://doi.org/10.1038/s41598-023-30591-2.

37. Nishio E, Iwata A, Kawasaki R, Iwao K, Nishizawa H, Fujii T. Metabolomic and microbiome analysis of cervicovaginal mucus in in vitro fertilization-embryo transfer: Toward predicting pregnancy success. Reprod Med Biol. 2024;23:e12568. https://doi.org/10.1002/rmb2.12568.

38. Wu Y, Shi L, Jin Z, Chen W, Wang F, Wu H, et al. A nomogram prediction model for embryo implantation outcomes based on the cervical microbiota of the infertile patients during IVF-FET. Microbiol Spectr. 2025;13:e0146224. https://doi.org/10.1128/spectrum.01462-24.

39. Bar O, Vagios S, Barkai O, Elshirbini J, Souter I, Xu J, et al. Harnessing vaginal inflammation and microbiome: a machine learning model for predicting IVF success. NPJ Biofilms Microbiomes. 2025;11:95. https://doi.org/10.1038/s41522-025-00732-8.

40. Alley AW, Łaniewski P, Bruno GT, Moffitt DV, Arani G, Farland LV, et al. Vaginal Microbiome Dominated by Lactobacillus Positively Impacts Clinical Pregnancy in Patients With Frozen Embryo Transfers. Am J Reprod Immunol. 2025;93:e70108. https://doi.org/10.1111/aji.70108.
